# Current status of next-generation vaccines against mpox virus: a scoping review

**DOI:** 10.3389/fphar.2025.1533533

**Published:** 2025-04-28

**Authors:** Luis Alberto Bravo-Vázquez, Daniela Bernal-Vázquez, Asim K. Duttaroy, Sujay Paul

**Affiliations:** ^1^ School of Engineering and Sciences, Tecnologico de Monterrey, Campus Querétaro, Querétaro, Mexico; ^2^ Department of Nutrition, Institute of Basic Medical Sciences, Faculty of Medicine, University of Oslo, Oslo, Norway

**Keywords:** mpox virus, vaccine, immunotherapy, antibody, mRNA therapeutics, recombinant antigen

## Abstract

**Introduction:**

The mpox disease, caused by the mpox virus (MPXV), has become a rising public health issue due to its potential to cause outbreaks. Consistently, this investigation aims to evaluate the current advances in the development of novel immunotherapeutic approaches against MPXV, which are crucial for preventing and controlling mpox spread.

**Methods:**

This scoping review was performed by analyzing the content of English-language articles published between 2018 and 2024, which reported the development of next-generation vaccines against MPXV and their assessment in animal models. Patents within the scope of this research were also included. Contrarywise, studies based solely on immunoinformatic methods, reviews, book chapters, news, and others were excluded. The literature search was executed in 11 databases, such as Scopus, MEDLINE, and PubMed.

**Results:**

A total of 36 records (32 studies and 4 patents) were included in this review. All 32 articles contain preclinical studies with varied group sizes (4–16) in which the main animal models were BALB/c mice. Less commonly used models included CAST/Ei mice and cynomolgus macaques. Moreover, most vaccines targeted one or more MPXV antigens, such as A29L, A35R, B6R, and M1R, through active immunization (via mRNAs or recombinant antigens) or passive immunization (antibody delivery).

**Conclusion:**

Overall, new generation vaccines might represent prospective candidates to combat the mpox health concern. Nonetheless, several of the analyzed studies possess drawbacks, including animal models with limited similarity to humans, small group sizes, and brief follow-up durations. Consequently, additional research is required to ascertain the long-term protection, efficacy, and safety of these immunotherapeutic approaches.

## 1 Introduction

Mpox (formerly known as monkeypox) is a zoonotic disease caused by the mpox virus (MPXV), which is a double-stranded DNA virus with an average genome size of around 170–250 kb. It is classified within the Poxviridae family and in the *Orthopoxvirus* genus, which also encompasses the variola virus responsible for smallpox (Branda et al., 2024; Zinnah et al., 2024). Since 2022, mpox has arisen as a significant priority for scientists, as the World Health Organization (WHO) proclaimed it as an urgent health crisis of worldwide concern due to the alarming global dissemination of the disease (Srivastava et al., 2023). Since January 1, 2024, 24,872 cases of clade I and clade II MPXV have been officially reported in 81 different locations (as of January 16, 2025) (Centers for Disease Control and Prevention, 2022). Individuals infected with MPXV tend to present a variety of symptoms that include, but are not limited to, fever, cough, rash, headache, confusion, abdominal pain, nausea, vomiting, and conjunctivitis (Niu et al., 2023). On the other hand, even though the mental health impact of mpox is still unclear, patients may suffer from anxiety, social isolation, stigma, panic, fear, and anger, among other psychological signs (Curtis et al., 2023).

The MPXV can spread through several mechanisms, including animal-to-animal, animal-to-human, or human-to-human, and the predominant mode of infection from animal to human is through close contact with the infected animal or its bodily fluids (Karagoz et al., 2023). Although the mortality rate of mpox has been reported to be around 1%–11% (Beer and Bhargavi Rao, 2019), this fatality rate is significantly influenced by the medical care supplied to the patients, the viral strain, and the immunological and health attributes of the infected persons (Hatami et al., 2023). Moreover, the hospitalization rate of mpox has been estimated to be approximately 6.34%, with data from nine studies considering 2,275 patients from the 2022 mpox outbreak (Li P. et al., 2023). Despite this, the recent COVID-19 pandemic has demonstrated that viruses can mutate quickly, acquiring more virulent properties that may set the world’s population at risk (Han et al., 2023; Williams et al., 2023). Hence, international authorities, along with stakeholders and scientists, must collaborate to ensure affordable access to mpox vaccinations and to encourage the international response to the mpox epidemic (Gruber, 2022; Lee et al., 2024).

Two principal forms of MPXV have been noticed during its pathogenic process, an extracellular enveloped virus (EEV) and an intracellular mature virus (IMV) (Lu et al., 2023; Sagdat et al., 2024). Therefore, the proteins displayed by these infectious viral entities have been identified as potential targets for the development of novel treatments and vaccines against MPXV due to their immunogenic activities. For instance, A29L, E8L, H3L, and M1R are proteins that belong to the IMV form, while A35R, B6R, and C19L are present in the EEV (Wang et al., 2023; Sagdat et al., 2024). Particularly, A29L and M1R participate in the cellular entrance stage of the mature virus, while A35R and B6R are recognized as molecular components involved in the transmission process of the enveloped virus (Sagdat et al., 2024). In spite of the advances in the discovery of potential MPXV drug targets, there is currently no specialized vaccine or drug approved for this virus (Gong et al., 2022; Haruna et al., 2022).

As an alternative, vaccines and medications previously approved for smallpox and other orthopoxviruses are being considered nowadays to fight MPXV infection due to the genomic similarity between these viruses (Yashavarddhan et al., 2023). The key vaccines authorized in some countries for this purpose are JYNNEOS (live, non-replicating, Modified Vaccinia Ankara-Bavarian Nordic vaccine, also known as MVA-BN, Imvamune, or Imvanex) and ACAM2000 (live, replicating vaccinia virus, VACV), while some of the most important anti-poxvirus drugs applied against MPXV are tecovirimat, cidofovir, brincidofovir, and vaccinia immune globulin (VIG) (Islam et al., 2022; Mohanto et al., 2023; Shamim et al., 2023; Yashavarddhan et al., 2023). Nonetheless, the efficacy of these vaccines and drugs against MPXV remains ambiguous; as well as they are not readily accessible worldwide (Webb et al., 2022; Jiang et al., 2024; Ganesan et al., 2025).

Mpox vaccine candidates have evolved over different generations that, as mentioned above, were mainly dependent on vaccines developed against smallpox. For instance, Dryvax, was a first-generation vaccine that used a live, replication-competent VACV obtained from calf lymph and was approved in 1931 by the FDA to protect from smallpox (Bryer et al., 2022; Chakraborty et al., 2022); however, this vaccine is no longer manufactured (Kajal et al., 2024). Indeed, ACAM2000 vaccine was licensed by the FDA in 2007 and replaced the Dryvax vaccine (Bryer et al., 2022). ACAM2000 is a second-generation vaccine for smallpox derived from a single-clonal isolate from the Dryvax vaccine that retains its replication competency but is produced with modern cell culture techniques to increase its safety (Frey, 2014; Poland et al., 2022). Even so, ACAM2000 poses a risk of severe side effects, such as myopericarditis, particularly in individuals without prior smallpox immunity, making it unsuitable for subjects with severe immunosuppression (Gruber, 2022). Third-generation vaccines include the live, attenuated, non-replicating JYNNEOS vaccine approved in 2019 by the FDA for the prevention of smallpox and mpox (Chakraborty et al., 2022; Gruber, 2022; Poland et al., 2022) and the highly attenuated vaccine LC16m8 (not yet approved by the FDA, but licensed in Japan) obtained in 1970 from the VACV Lister strain of the first-generation smallpox vaccines (Poland et al., 2022; Sudarmaji et al., 2022; Grabenstein and Hacker, 2024). Finally, OrthopoxVac is a fourth-generation live vaccine against smallpox, mpox, and other orthopoxviruses based on the VACdelta6 strain that received approval from the Russian Federation in 2022 (Shchelkunova and Shchelkunov, 2023; Liu, 2024; Kumar et al., 2025).

Notwithstanding the above, in light of the incidence of new outbreaks (Cevik et al., 2024; Sah et al., 2024) and the possible occurrence of mutations in the MPXV genome (Jahankir et al., 2024; Shen-Gunther et al., 2025), the development of next-generation vaccines designed exclusively for MPXV is mandatory (Fantin and Coelho, 2024; Liu et al., 2024). Next-generation vaccines can be defined as those that are addressed on targeting specific immunodominant antigens (e.g., via the delivery of mRNAs or recombinant proteins) to promote immune responses instead of relying on a whole inactivated or live attenuated infectious agent (Dormitzer et al., 2008; van Riel and de Wit, 2020; Rezaei and Nazari, 2022; Khalid and Poh, 2023). Some of the pioneering reports in this field were focused on developing new generation vaccines against MPXV based on VACV antigens.

**FIGURE 1:**
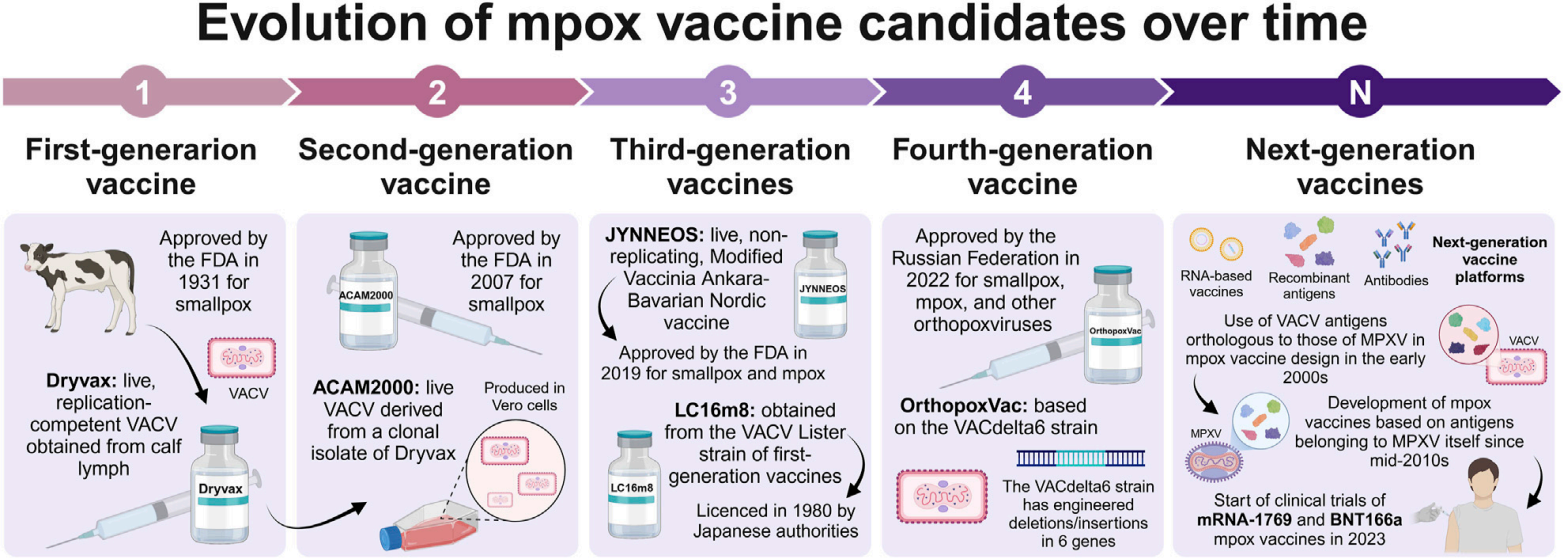
Pictorial representation of the evolution of mpox vaccine candidates across time (created with a licensed version of BioRender.com).

In this context, Hooper et al. (2003) identified that the antibodies produced by rhesus macaques (*Macaca mulatta*) after immunization with a plasmid DNA vaccine containing the A27L, A33R, B5R, and L1R genes from VACV cross-reacted with MPXV orthologous proteins, demonstrating the forthcoming importance of these antigens in the design of mpox vaccines. Afterward, Heraud et al. (2006) generated both plasmid DNA and recombinant protein vaccines based on VACV, A27L, A33R, B5R, and L1R proteins; however, only recombinant proteins adjuvanted with CpG or a DNA prime/protein-CpG boost regimen protected rhesus macaques from the lethal MPXV challenge. Buchman et al. (2010) showed that the recombinant VACV proteins A27, A33, B5, and L1 containing CpG/alum were effective against MPXV lethal infection in cynomolgus macaques (*Macaca fascicularis*). Hirao et al. (2011) tested a plasmid DNA vaccine encoding VACV A4L, A27L, A33R, A56R, B5R, F9L, H3L, and L1R proteins that protected cynomolgus macaques from the lethal MPXV challenge. On the other hand, one of the first vaccine candidates centered on specific MPXV antigens was reported by Franceschi et al. (2015). These researchers designed vaccines based on recombinant bovine herpesvirus 4 (BoHV-4) vectors carrying MPXV antigens (A29L, B6R, or M1R). Particularly, M1R alone or in combination with A29L and B6R resulted effective against the MPXV lethal challenge in STAT1^(−/−)^ mice. However, current research on new-generation vaccines using antigens belonging to MPXV itself is still in progress and there is uncertainty regarding the efficacy and safety of these emerging immunotherapies. The timeline of the evolution of mpox vaccine candidates is illustrated in Figure 1.

Although a handful of published reviews (Abdelaal et al., 2022; Papukashvili et al., 2022; Lee et al., 2023; Saadh et al., 2023; Garcia-Atutxa et al., 2024) have focused on the development of novel mpox vaccines, relevant reviews that follow strict Preferred Reporting Items for Systematic Reviews and Meta-Analyses (PRISMA) guidelines (Tricco et al., 2018; Page et al., 2021) are limited. Moreover, several existing literature reviews, systematic reviews, and meta-analyses are centered mainly on the application of smallpox vaccines against MPXV (Ghazy et al., 2023; Kang et al., 2023; Malik et al., 2023; Eslami et al., 2024; Grabenstein and Hacker, 2024; Pischel et al., 2024) rather than analyzing the advances in next-generation vaccines designed exclusively for MPXV. Furthermore, despite the fact that a couple of next-generation mpox vaccines are currently in ongoing clinical trials, the data from preclinical trials in animal models can be of high relevance to guide virologists and policymakers in the development of new immunotherapeutic platforms that may avoid the emergence of a mpox pandemic. Consistently, the current scoping review critically evaluated the latest advancements in mpox vaccine development, as well as highlighted the knowledge gaps in this research field. Moreover, it is worth mentioning that this scoping review is not an update or a repetition of previous works; rather, it aims to discuss the current advances and future directions in the development of next-generation vaccines against MPXV.

## 2 Methods

The elaboration of this scoping review was based on the PRISMA 2020 guidelines (Page et al., 2021) and on the PRISMA Extension for Scoping Reviews (PRISMA-ScR) (Tricco et al., 2018). All the methodology was performed independently by two reviewers (L.A.B.-V. and D.B.-V.) without automation tools, and any kind of uncertainty was discussed with a third reviewer (S.P.). No additional protocol was prepared or registered apart from the methodology presented herein.

### 2.1 Eligibility criteria

For this scoping review, we considered studies published from 2018 to 2024 and written in the English language. The selection process was based on the PICO framework as follows: animal models (Population) immunized with next-generation vaccines against MPXV (Intervention), in which antibody elicitation, cell immune response, and/or reduction in disease severity after viral challenge (i.e., recovery of weight loss and survival) were evaluated (Outcome). The Comparison term of the PICO framework was not applied at all in this case since the studies by themselves already compared the effect of the mpox vaccines between vaccinated and control groups. Hence, studies that reported the immunological effects of next-generation mpox vaccines in animal models were the main target of this scoping review. Published patents were also included if they were centered on new generation vaccine designs exclusively against MPXV. Conversely, we did not consider reports that were based solely on bioinformatic tests. Articles were also not selected if they were centered on the development of vaccines against exclusively other viruses (e.g., SARS-CoV-2, smallpox, and camelpox). Finally, review articles, book chapters, comments, conference abstracts, retracted articles, and news, among other types of scientific communications, were also excluded.

### 2.2 Search strategy, information sources, and study selection

All the literature searches were carried out on August 22, 2024. In order to maximize the reach of the searches, we consulted 11 databases during the search strategy. These databases were Scopus, MEDLINE (via ProQuest), Web of Science, PubMed, Academic Search Ultimate (EBSCOhost), SpringerLink Journals, Gale Academic OneFile, Taylor & Francis Journals, Wiley Online Library, Directory of Open Access Journals (DOAJ), and PATENTSCOPE. We restricted the searches to abstracts (abstract and title in the case of PubMed) in order to avoid gathering studies that were outside the focus of this research. These searches were limited to 6 years ago. Moreover, the combination of keywords was meticulously designed and evaluated with preliminary searches to ensure its effectiveness based on the inclusion/exclusion criteria (no entries were gathered in these initial explorations). Important information regarding the searches in these databases, such as the keywords used, dates of coverage, and search strings are presented in Supplementary Table S1.

We performed every database search in triplicate using the aforementioned strings and dates to validate their reproducibility. After eliminating duplicated records, the titles and abstracts of the remaining entries were reviewed following the previously defined inclusion/exclusion criteria in order to include or exclude them. Subsequently, the full texts of the records potentially meeting the inclusion criteria were sought for retrieval. At this stage, all non-open access articles were obtained with the credentials of our institute or via the interlibrary loan services of our institution (Tecnologico de Monterrey). Finally, the full-text articles were reviewed and either included or excluded according to the eligibility criteria.

### 2.3 Data extraction and data items

The process of data extraction was carried out by examining the full-texts of the included articles. The data items obtained from these articles included the first author and publication year, the animal model used in the study, type of vaccine administered, MPXV clade and/or isolate addressed by the vaccine design, targeted antigens, immunization scheme, placebo type, viral strain and viral dose used in challenge, and survival rates. To avoid misinterpretations of data that are usually reported in a heterogeneous manner across studies or are only illustrated in graphs or figures, such as antibody titers and levels of cellular responses generated by vaccines, these data were addressed based on the interpretation provided by the authors of each study. Finally, the data items extracted from the patents consisted of title, first author, publication year, application country and company or institution, type of vaccine, targeted antigens, country or office, and patent number.

### 2.4 Data synthesis and analysis

The data synthesis and analysis were carried out by categorizing and discussing the studies based on the type of vaccine. These categories included mRNA-based vaccines, recombinant antigens of the MPXV, antibodies targeting MPXV antigens, and circular RNAs (circRNAs) encoding MPXV antigens. The content of each article was reviewed within the text, with a summary of the data presented in one table. On the other hand, the information on the identified patents was summarized in another table.

### 2.5 Risk of bias assessment

Although the risk of bias assessment is not mandatory for scoping reviews, we decided to include such evaluation to give added value to this work and so that readers who consult the information analyzed herein are aware of the possible risks of bias in the included studies. In this regard, we assessed the risk of bias based on some of the key recommendations for evaluating preclinical studies stated by Landis et al. (2012) and Macleod et al. (2015), which are adapted to evaluate factors relevant to laboratory studies in animal models. The levels of risk were set as low, unclear, or high. The domains of this assessment considered random assignation of animal models to experimental groups, complete descriptions of the methodology for the evaluation of outcomes, the inclusion of a clear justification of sample size to ensure that the studies were appropriately powered, and blinded measurement of outcomes. Additionally, we evaluated whether the studies reported an ethical statement and approval for animal experimentation. Finally, potential investigator conflicts of interest were also considered in the assessment.

## 3 Results

### 3.1 Selection of sources of evidence

**FIGURE 2:**
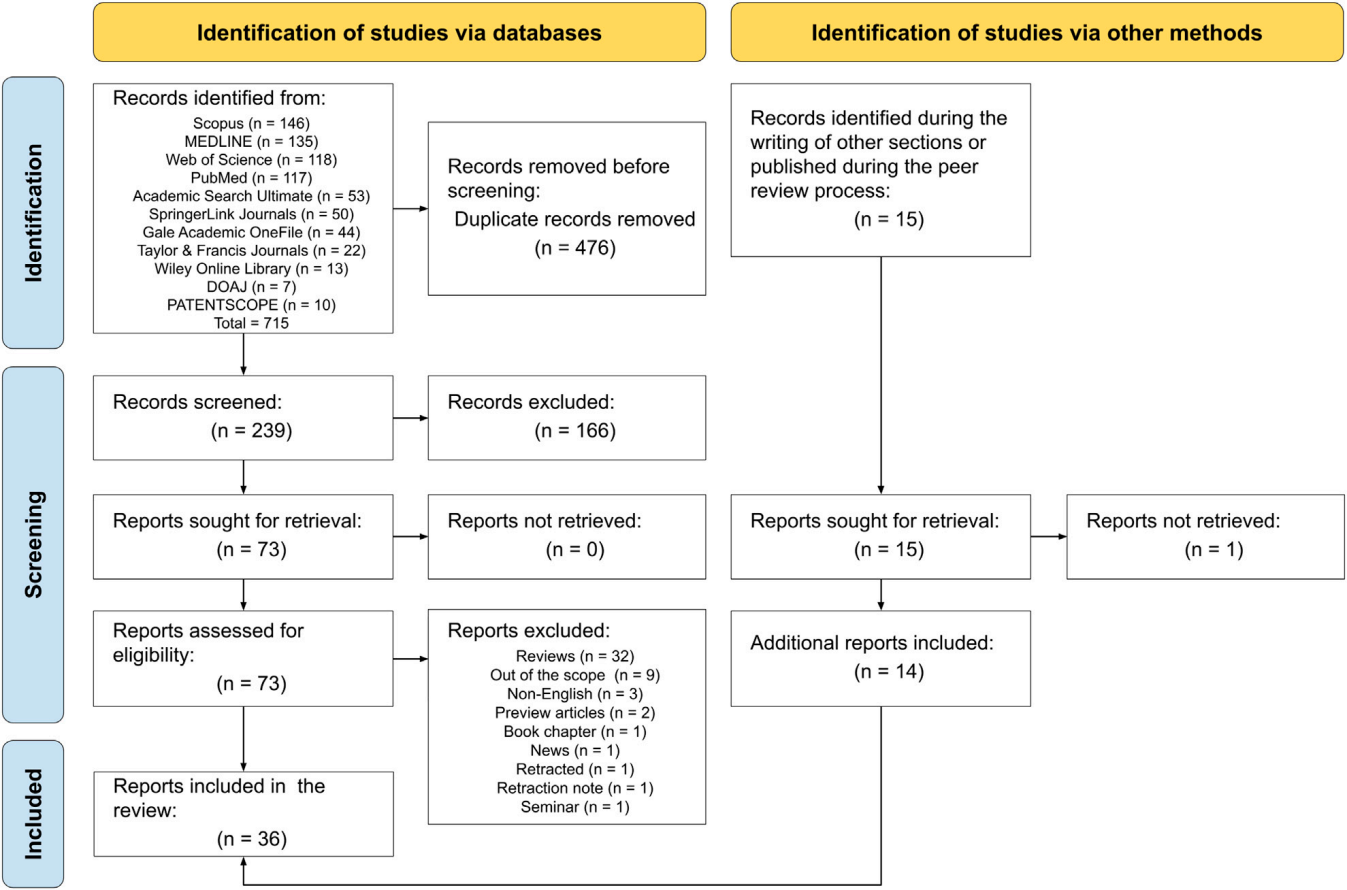
Flow diagram of the search methodology followed in this scoping review. The methodology was conducted following the PRISMA guidelines.

In this study, initially, 715 records were retrieved from the consulted databases, and then 476 duplicates were removed. Subsequently, the abstracts and titles of the remaining 239 records were screened and 166 were excluded due to non-relevance. The full-texts of the 73 remaining records were successfully retrieved and consequently, 51 entries were eliminated following the inclusion/exclusion criteria stated in the methodology. Throughout the writing of other sections of the manuscript and during the peer review process, 15 additional records were found, 14 of them were successfully included, and 1 could not be retrieved (i.e., Chuai et al., 2025). In the end, 36 records were included, comprising 32 studies and 4 patents. This selection process is depicted in Figure 2.

### 3.2 General overview of the included studies

The majority of the proposed vaccine designs against MPXV are mRNA vaccines loaded into lipid nanoparticles (mRNA-LNP) to facilitate the delivery of the coding sequences of MPXV antigens (19 studies out of 32). Other vaccine types reported were recombinant antigens of MPXV (8 studies out of 32), antibodies or mRNAs encoding antibodies against MPXV antigens (4 studies out of 32), and circRNAs encoding antigens of MPXV (1 study out of 32). The most common animal model used in the reports for evaluating the vaccines corresponded to BALB/c mice (present in at least 26 studies). Other less recurrent animal models were CAST/Ei mice, C57BL/6 mice, cynomolgus macaques, and dormice. Moreover, almost all the vaccine approaches were intended to target one or multiple antigens belonging to MPXV (e.g., A29L, A35R, B6R, E8L, H3L, and/or M1R), either via active immunization (delivery of the antigens through mRNAs or recombinant proteins) or via passive immunization (delivery of antibodies targeting the MPXV antigen). After immunization, the animal models were challenged with the VACV strains Tian Tan (TT) or Wester Reserve (WR) in most of the studies in order to evaluate the protection conferred by the vaccines. However, six studies did not perform this assessment. Besides, only eight articles provided information about the protective effect of a vaccine in animal models infected with MPXV after vaccination. All the included studies are cited chronologically in Supplementary Table S2, along with the relevant data extracted from them.

### 3.3 mRNA-based vaccines against MPXV

The emergence of mRNA vaccines gained the attention of the scientific community during the COVID-19 pandemic, demonstrating their rapid development potential and effectiveness. These vaccines work by encoding proteins from infectious agents, such as viruses, which serve as antigens capable of triggering immune responses (Heinz and Stiasny, 2021). This technology can also be applied to deliver mRNAs that encode antibodies targeting specific antigens (Deal et al., 2021). In both cases, the most common method for delivering mRNAs is through LNP formulations. LNPs are used because they protect the mRNA from degradation, improve cellular uptake, and enable the release of the mRNA into the cytoplasm for protein translation (Heinz and Stiasny, 2021).

In this matter, Fang et al. (2023) created two candidate vaccines for MPXV, MPXVac-097, which encoded the A29L, A35R, B6R, E8L, and M1R antigens, and a mixture of the five individual mRNA-LNPs termed Mix-5. The MPXVac-097 multi-antigen mRNA-LNP vaccine exhibited neutralizing efficacy against cowpox, VACV, and MPXV, with antibody titers rising in a dose number-dependent manner. A comparison of MPXVac-097 with Mix-5 demonstrated that both vaccines induced similar binding antibody responses, especially against antigens A35R and E8L, across different dosage regimens. Furthermore, both MPXVac-097 and Mix-5 conferred protection in mice challenged with VACV, as vaccinated animals sustained steady body weight and displayed only minor symptoms, in contrast to the substantial weight loss and clinical manifestations observed in unvaccinated controls. Safety assessments verified that MPXVac-097 was well-tolerated, exhibiting no notable pathological signs in immunized mice. The lack of toxicity-related alterations and the strong immunogenicity indicate that MPXVac-097 is a suitable option for mpox vaccination.

Zhang R. R. et al. (2023) created mRNA-LNP vaccines that were formulated using different combinations of viral antigens from MPXV. The vaccines, designed with equal amounts of mRNA-LNPs, were identified as AR-MPXV5 (A29L, A35R, B6R, E8L, and M1R), AR-MPXV4a (A29L, B6R, E8L, and M1R), AR-MPXV4b (A29L, A35R, B6R, and E8L) and AR-MPXV3 (A29L, B6R, and E8L). BALB/c mice were immunized with each mRNA-LNP formulation, followed by a booster dose after 3 weeks, and were challenged with VACV TT. AR-MPXV5 and AR-MPXV4a vaccinated groups experienced minor weight loss, while the placebo group exhibited a significant decrease in weight. Nonetheless, all animals in the control and vaccinated groups survived the viral challenge. The viral load in the vaccinated groups was barely detectable, with AR-MPXV4b showing no presence of infectious particles or viral DNA. AR-MPXV5 and AR-MPXV4a produced high levels of neutralizing antibodies, while no neutralizing antibodies were detected in AR-MPXV4b and AR-MPXV3 after the first immunization. However, following the second immunization, neutralizing antibodies were detected in both AR-MPXV4b and AR-MPXV3 groups, though at lower levels compared to AR-MPXV5 and AR-MPXV4a. This preclinical analysis demonstrated that all the vaccines protected mice from VACV TT. Nevertheless, AR-MPXV5 and AR-MPXV4a triggered the strongest immune response, as they generated significantly higher levels of neutralizing antibodies, offering effective protection against VACV exposure (Zhang R. R. et al., 2023). This research highlights the critical role of specific antigen combinations in enhancing vaccine efficacy. In fact, further exploration of the efficacy of the AR-MPXV5 vaccine unveiled that the administration of two doses in cynomolgus macaques successfully induces both antibody and cellular immune responses. In a challenge model using a contemporary MPXV clade II strain, the AR-MPXV5 vaccine effectively prevented skin lesions, cleared viremia, and reduced viral loads across multiple tissues in naive male non-human primates. Outstandingly, the vaccine was well-tolerated in rhesus macaques with chronic simian immunodeficiency virus infection, generating MPXV-specific humoral and cellular responses comparable to those observed in healthy animals (Ye Q. et al., 2024).

In another effort to develop effective immunogenic platforms against MPXV, Sang et al. (2023) developed two mRNA-based vaccines targeting antigens derived from two IMV-specific (i.e., A29L and M1R) and two EEV-specific proteins (i.e., A35R and B6R). These vaccines were named as mRNA-A-LNP and mRNA-B-LNP. Among the tested antigens, M1R and A35R produced high antibody titers. Moreover, the second dose of the mRNA-A-LNP vaccine was more effective in generating neutralizing antibodies. To assess the protective capability of the vaccines, immunized BALB/c were challenged with VACV TT. Remarkably, both vaccines prompted a rapid recovery, as viral load measurements indicated that, within 24 h post-vaccination, VACV was nearly undetectable in the immunized mice, while the control group displayed evident viral presence. Besides, a safety assessment indicated that the mice did not lose weight and showed no signs of inflammation or local skin reactions (Sang et al., 2023). These findings suggest that the vaccines developed are effective and safe, making them promising mpox vaccine candidates.

Two other mRNA-LNP vaccines were designed by Zeng et al. (2023) to encode four MPXV antigens (i.e., A29, A35, B6, and M1) designated as Rmix4 and Lmix4, or six MPXV antigens (i.e., A29, A35, B6, E8, H3 and M1) labeled as Rmix6 and Lmix6. In the case of Lmix vaccines, mRNAs were directly encapsulated into LNPs using the same antigens as the Rmix. In contrast, Rmix formulations were prepared by mixing linearized plasmids encoding the antigens (four or six, respectively) and then transcribing the mixture into mRNA before encapsulation into LNPs. Notably, Rmix6 had a higher T-cell response compared to Rmix4. All mRNA-LNP vaccine candidates incorporating multiple antigens generated comparably strong cross-protective immune responses against VACV. After immunization and viral challenge in BALB/c mice, L/Rmix4 and L/Rmix6 provided significant protection against VACV WR. In the control group, almost all mice died and exhibited significant weight loss, while in the vaccinated groups, all mice survived and maintained constant weight. Furthermore, the antigen M1 promoted the production of high levels of neutralizing antibodies, which indicates its importance in vaccine design (Zeng et al., 2023). Overall, the proposed multi-antigen mRNA-LNP vaccine could be a favorable approach for producing substantial immune responses against MPXV.

Zhang N. et al. (2023) tailored four mRNA-LNP vaccines, harnessing different combinations of surface antigens belonging to MPXV. Accordingly, four different combinations were generated: MPV-E2, including A35R and B6R from the enveloped virion (EV); MPV-M2, composed of H3L and M1R from the mature virion (MV); MPV-M4 combining A29L, E8L, H3L, and M1R; and MPV-EM6 combining A29L, A35R, B6R, E8L, H3L, and M1R. The vaccines were tested on BALB/c mice, and the results indicated that the MPV-EM6 vaccine provided a higher protection against the VACV TT challenge. In this context, A35, E8L and M1R were the antigens that produced more antibodies, which continued to increase with the booster dose. In terms of neutralizing antibodies, MPV-EM6 also induced the highest antibody levels. Across all groups that were immunized, the mice maintained stable body weight, displaying complete protection with MPV-EM6 and MPV-M4 vaccines. While the control group lost significant body weight and mice were sacrificed after 6 days (Zhang N. et al., 2023). These outcomes highlight the potential of these multi-antigen vaccines as promising candidates against MPXV.

Other mRNA-LNP vaccine candidates were created by Xia et al. (2023) using antigens from MPXV (A29L, A35R, B6R, and M1R) that are homologous to those of VACV (A27, A33, B5, and L1), and was tested in C5BL/6 mice by administering the antigens individually or as a cocktail. Afterward, mice were challenged with the VACV TT strain to assess the efficacy of the vaccine. The study revealed that, at high doses, the vaccines significantly reduced the viral load of mice, and the mixed cocktail promoted the survival rate in all mice. Intriguingly, all the vaccines stimulated strong antibody responses (especially the mRNA-LNP vaccines at 5 μg), suggesting that they were the most effective ones. Notably, mice that were vaccinated quickly recovered their body weight and reduced their viral load, being the 5 μg dose the one showing the strongest effect. In contrast, the control group, which was injected with PBS, experienced a decrease in their body weight and could not recover from the challenge. Overall, the cocktail containing 0.5 μg from each mRNA-based antigen provided full protection against VACV, while the 5 μg dose of individual antigens offered full protection against VACV except for the A33, which did not protect entirely against VACV (Xia et al., 2023). These findings show once again the putative importance of mRNA-LNP against mpox outbreaks, ensuring a rapid recovery with minimal side effects.

Hou et al. (2023) constructed a set of mRNA-LNP vaccines against MPXV, namely, VGPox 1 and VGPox 2, which encoded the extracellular domain of A35R, a signal peptide, and an M1R antigen. The other vaccine was VGPox 3, which contained a mixture of the mRNAs encoding A35R and M1R encapsulated in LNPs. In a single-dose experiment, BALB/c mice were immunized and challenged with VACV WR, which provided rapid protection, as immunized mice showed no significant weight loss, while the control group lost body weight significantly. Neutralizing antibodies were also evaluated, and it was found that VGPox 1 and VGPox 2 had higher neutralization activity. Nonetheless, all three vaccine types protected mice from VACV. In a further experiment, at 162 days post-vaccination, mice were exposed intranasally to a lethal dose of VACV WR, and all three mRNA vaccine formulations conferred full protection (Hou et al., 2023). These findings suggest that the three mRNA-LNP vaccines could provide sustained protection against MPXV.

Freyn et al. (2023) developed mRNA-LNP vaccines encoding four surface proteins (A29, A35, B6 and M1) from MPXV. They investigated different concentrations of individual antigens, as well as different combinations of the antigens. Following the immunization of BALB/c mice, single MPXV antigens provided partial protection against VACV WR, with M1 producing the highest neutralizing antibody response at a concentration of 0.5 µg or 2 µg. Mice that received a 2 μg dose of the MPXV mRNA vaccine exhibited full survival, experiencing only minimal and temporary weight reduction following the challenge. Likewise, the 8 μg mRNA multi-antigen vaccines allowed complete survival and no significant weight loss after exposure to VACV (Freyn et al., 2023). Consistently, these observations indicate that multi-antigen combinations reported in this investigation deserve further exploration.

Subsequently, two mRNA-LNP vaccines encoding the surface proteins A29L, M1R, A35R and B6R antigens from MPXV were designed by Yang et al. (2023a). The vaccines were named MPXfus and MPXmix. MPXfus is a system in which the four antigens are combined together and encoded by a single mRNA as a fusion protein, and MPXmix is a multicomponent formulation that contains four individual mRNA-LNPs, each one containing the antigens in equal amounts. BALB/c mice were immunized, and after the first dose, anti-M1R and anti-A35R antibody titers induced by the MPXfus vaccine were higher than those induced by MPXmix. Both groups of vaccinated mice developed neutralizing antibodies against VACV, as confirmed through a virus neutralization assay. This implied that the vaccines can induce an immune response against poxviruses (Yang et al., 2023a). Despite these observations, this study did not assess the effectiveness of disease reduction with a viral challenge, limiting its conclusions about the protective potential of the vaccines MPXmix and MPXfus.

Zuiani et al. (2024) created two mRNA-LNP vaccines against MPXV, namely, BTN166a (encoding A35, B6, H3, and M1) and BTI166c (encoding A35, B6 and M1). The BTN166 vaccines were tested in four biological models, which included Wistar rats, BALB/c, CAST/Ei mice, and cynomolgus macaques. Nevertheless, the viral challenge was just performed in the last three animal models. Each model animal received two doses of BTN166 vaccines or individual antigens (BALB/c mice), and was challenged with either VACV WR (BALB/c mice) or Clade I MPXV (CAST/Ei mice and cynomolgus macaques). Particularly, the antigens A35, B6 and M1 provided complete protection against VACV WR in BALB/c mice. However, A35 combined with B6 were less effective in the Clade I MPXV challenge model (CAST/Ei mice). Remarkably, the BNT166a vaccine, which combines multiple antigens, resulted in better immune responses (Zuiani et al., 2024). Mice immunized with BTN166 vaccines did not experience a significant percentage of weight loss, while the control group showed considerable weight loss. Moreover, BTN166 vaccines demonstrated substantial survival rates, especially BTN166a showed the best results in the two mice models. In the case of cynomolgus macaques, which were challenged with Clade I MPXV, BNT166a vaccination provided considerable protection. After the challenge, initially, the vaccinated macaques showed minimal weight loss that quickly resolved, and all of the BNT116a immunized macaques survived, which highlights the vaccine’s robust effects across different species (Zuiani et al., 2024). This report broadly describes the effects of vaccines on various organisms, making BNT166a one of the most compelling vaccine proposals for mpox management.

(Chi et al., 2024) engineered six mRNA combinations that encode antibodies aimed at targeting EEV and IMV surface proteins in order to neutralize orthopoxviruses. Each antibody was designed to target a specific antigen from VACV or MPXV, being mRNA-mab301-LNP for antigen A27 (VACV), mRNA-mab22-LNP for antigen A33 (VACV), mRNA-mab283-LNP for antigen B5 (VACV), and mRNA-mab26-LNP for antigen M1 (MPXV). Additionally, two combinations of these mRNAs, Mix2a (mRNA-mab22-LNP and mRNA-mab26-LNP) and Mix2b, were formulated; however, the content of Mix2b formulation is not clearly stated within the report. BALB/c mice were immunized with individual mRNA-LNPs or cocktails and challenged with VACV WR. Rapid antibody production after a single dose of each vaccine was noticed, especially for mRNA-mab26, which showed the higher level. In addition, the results indicated that Mix2a and Mix2b provided superior protection, as mice immunized with Mix2a and Mix2b also showed negligible weight loss. Mix2a and Mix2b effectively cleared infectious virus from lung tissue and prevented VACV-induced lung pathology. Besides, from the individual mRNA-LNP vaccines, mab22 provided complete protection against weight loss and mortality, with no detectable levels of VACV virus particles in the lungs. Importantly, all monoclonal antibodies and Mix vaccines guaranteed survival of the challenged mice. Overall, these mRNA-encoded antibodies are promising candidates for the prevention of orthopoxvirus outbreaks, particularly of MPXV.

More recently, Su et al. (2024) created a vaccine termed ALAB-LNP to express four VACV antigens (A27, L1, A33 and B5) arranged in tandem within a single molecule. This vaccine was compared to another formulation called 4Sin-LNP, which expressed a mixture of the same antigens as individual mRNA-LNPs. In the experiment, both BALB/c mice and Sprague Dawley rats received two doses of the vaccines. The results indicated that mice immunized with varying doses of ALAB-LNP or 4Sin-LNP generated strong antibody responses against L1, A33, and B5 after the first dose, and the levels of the antibodies against the four antigens increased after the boost immunization. Additionally, all the vaccine formulations produced neutralizing antibodies after the second dose, being ALAB-LNP (particularly at 20 µg), the one that had the highest production. The vaccines also showed cross-neutralizing activity against MPXV. However, the study did not evaluate survival rates of immunized animal models challenged with VACV or MPXV, leaving gaps in understanding the protective effects of the vaccines. Thus, additional testing is urged, particularly focusing on the vaccines’ efficacy and their possible adverse effects.

Four mRNA-LNP vaccines, designated as LBA (A29L and B6R), LAM (A35R and M1R), a cocktail LBAAM (A29L, A35R, B6R, and M1R), and LBA&LAM, which combined LBA and LAM vaccines, were developed by Ye T. et al. (2024). To evaluate their efficacy, BALB/c mice were immunized, followed by a challenge with VACV TT in order to assess the vaccines’ efficacy. The results showed that LAM and LBA&LAM vaccines produced high levels of neutralizing antibodies against MPXV. In relation to the efficacy of the vaccines, mice immunized with the different formulations showed better protection against VACV than the control group. Mice in the control group experienced substantial weight loss after being exposed to VACV TT and all the animals in this group were euthanized, while the vaccinated groups did not experience a significant decrease in body weight, being LBA&LAM and LBAAM the ones that maintained a stable body weight throughout the challenge and promoted quick recovery from the viral challenge. Importantly, all vaccinated groups survived the challenge, demonstrating the effectiveness of these mRNA-LNP vaccines.

A multi-antigen mRNA-LNP vaccine, called MPXV-1103, was tailored by Li E. et al. (2024) containing A35, A29, B6, and M1 surface proteins from the MPXV in a single sequence linked together by three (G_4_S_1_)_3_ flexible linkers. Animal studies were performed to test MPXV-1103, the individual mRNA-LNPs of the antigens, as well as a vaccine called Mix-4-LNP (a mixture of the mRNA-LNPs of the individual antigens). The vaccines were evaluated in BALB/c mice that received two doses of the same and were later challenged with VACV TT. Post-immunization results revealed that MPXV-1103 induced high amounts of antibodies at all doses, which displayed a significant neutralizing activity. In terms of efficacy, all the vaccinated mice recovered weight at 4 days post-infection. Successfully, all mice immunized with any of the vaccines survived, while LNP and PBS controls did not survive. Additionally, viral load analysis revealed that the MPXV-1103, A35, and M1 vaccines led to low or undetectable levels of viral DNA and particles. Subsequent research on the MPXV-1103 vaccine demonstrated its ability to induce sustained humoral and cellular immune responses in BALB/c mice, generating IgG antibodies specific to MPXV antigens A29, A35, B6, and M1, while also activating cytotoxic CD8^+^ T cells. These immune mechanisms conferred complete protection against a lethal VACV TT challenge, even at 280 days post-vaccination (Li et al., 2025). These results imply the remarkable potential of MPXV-1103 for combating mpox.

More recently, in October of 2024, Mucker et al. (2024) reported the outcomes of an investigation in which the protective efficacy of the mRNA-LNP vaccine, mRNA-1769, which encodes optimized versions of four key MPXV antigens (A29L, A35R, B6R, and M1R) was tested in a non-human primate model of cynomolgus macaques challenged with clade I MPXV Zaire 1979. The vaccine conferred complete protection against the lethal MPXV. Moreover, animals vaccinated with mRNA-1769 exhibited a tenfold reduction in lesion count, a shorter disease duration, and significantly lower circulating and mucosal viremia levels compared to those immunized with MVA. Detailed immunological profiling revealed that mRNA-1769 induced stronger MPXV-specific neutralizing responses, broader heterologous neutralizing titers, and more robust humoral immune functions against the four MPXV antigens than MVA immunization. Later, the same group of scientists demonstrated that the mRNA-1769 vaccine effectively protects mice against intranasal and intraperitoneal MPXV challenges. Besides, a single dose of the vaccine conferred substantial protection, which was further enhanced by a booster, maintaining efficacy for at least 4 months. Also, immunodeficient C57Bl/6 Rag2 KO mice that could not produce mature B and T cells exhibited protection when administered serum from mRNA-1769-immunized macaques before the VACV WRvFire challenge (Cotter et al., 2024). These findings indicate that the mRNA-1769 vaccine not only provides robust protection against the lethal MPXV challenge but also offers enhanced disease mitigation, highlighting potential advancements in mpox vaccine development.

In another research, Kong et al. (2024) developed trivalent mRNA-LNP vaccines that encode single-chain immunogens containing soluble regions of the MPXV antigens A35R, B6R, and M1R; these vaccines were called AMAB-wt, AMAB-C140S, and AMB-C140S. Strong neutralizing antibodies against VACV and MPXV were noticed with the three vaccines. As well, these vaccines also provided remarkable protection in BALB/c mice lethally challenged with VACV WR and significantly reduced viral load after MPXV challenge. The single-chain or cocktail mRNA vaccines encoding the soluble antigens conferred 100% or 80% (in the case of AMAB-wt, 8.1 × 10^5^ PFU VACV WR) survival against a lethal VACV challenge, whereas a cocktail of the full-length antigens demonstrated comparatively lower protection. It is important to mention that, in the case of the challenge with WIBP-MPXV-001, all groups of BALB/c mice survived, so it is suggested to test this vaccine in an animal model with greater susceptibility to MPXV.

An additional mRNA-LNP vaccine was proposed by Tian et al. (2024) to express the antigen A29L from MPXV. To test this vaccine, BALB/c mice were immunized with two doses of the formulation, followed by a challenge with VACV TT. The A29L vaccine exhibited strong positive effects against VACV, significantly reducing viral load. Additionally, the percentage of body weight in the immunized group slightly decreased, and mice quickly recovered, reflecting the activation of immune defenses. Nevertheless, none of the mice in the two groups (vaccinated and controls) succumbed to the infection. The observations also suggested that the A29L mRNA-LNP vaccine did not have adverse effects such as substantial cutaneous reactions. Moreover, the vaccine successfully induces a robust immune response by cross-neutralizing both VACV and MPXV, making it a safe and effective vaccine. Hence, further research should be conducted on the A29L mRNA vaccine, aiding in the development of vaccines against MPXV.

### 3.4 Vaccines based on recombinant MPXV antigens

Recombinant antigens have become a key component in the development of next-generation vaccines, particularly in response to emerging infectious diseases such as mpox. These antigens are produced by expressing viral proteins in platforms, including *Escherichia coli*, yeast, insect cells, or mammalian cell lines like CHO cells (de Pinho Favaro et al., 2022). Once purified, these proteins serve as antigens that stimulate the immune system to recognize and respond to the infectious agent. Intriguingly, recombinant antigen technology is advantageous in vaccine production due to its scalability, high yields, and cost-effectiveness (Khalid et al., 2024).

A recombinant vaccine targeting antigens from MPXV was developed by Gao et al. (2023). Their first assessment aimed to determine whether vaccination with MVA could induce antibodies that cross-react with antigens from MPXV. The experiment was conducted on BALB/c mice that received two doses of MVA on days 0 and 21 at different viral concentrations, including 10^5^ TCID_50_/mL, 10^6^ TCID_50_/mL, or 10^7^ TCID_50_/mL. Researchers collected serum samples from the vaccinated mice, as those samples contained the antibodies produced after MVA immunization. The serum from the MVA-immunized mice was used in ELISA assays in order to test if the antibodies would bind to A29, A35, B6, H3, I1 and M1 despite the fact that MVA does not express these specific antigens. These results suggest that given the high similarity between MVA and MPXV, the neutralizing antibody response induced by MVA immunization is likely to cross-react with MPXV. Additionally, the highest immune responses occurred with the 10^7^ TCID_50_/mL concentration (the highest dose) (Gao et al., 2023).

In the second phase of the study (Gao et al., 2023), recombinant versions of MPXV antigens were synthesized using CHO cells. Although M1 and A29 elicited stronger neutralizing antibody responses, researchers decided to include other antigens such as A35, B6, H3 and I1 for further investigation. These recombinant antigens were tested for their ability to induce neutralizing antibodies against MVA. BALB/c mice were immunized twice with these antigens, either alone or combined with the adjuvant AddaVax to enhance immune responses. Adjuvanted A29, I1, and H3 antigens induced antibody responses four times higher than unadjuvanted groups, demonstrating the adjuvant’s immune-enhancing effect. Meanwhile, IgG antibody levels for A35, B6, and M1 remained comparable across groups. On the other hand, mice vaccinated with A29 and M1 developed significant cross-neutralizing antibodies that were effective against MVA (Gao et al., 2023). One drawback of this study is the absence of viral challenge, which could have provided additional information about the vaccine’s effectiveness. However, the findings showed the prospective role of M1 and A29 in the future development of recombinant antigen-based vaccines for the current mpox outbreak.

Tang et al. (2023) assessed the immunogenicity of a vaccine containing soluble forms of the MPXV recombinant antigens A29L, A35R, M1R, and B6R, along with its protective efficacy against the 2022 mpox mutant strain in BALB/c mice. Following vaccine administration with QS-21 adjuvant, antibody titers increased sharply after the initial boost. The vaccine-induced neutralizing antibodies effectively suppressed MPXV replication and minimized organ pathology in infected mice. Body weight measurements remained consistent across all three experimental groups (vaccinated, QS-21 alone, and PBS) that were challenged with the MPXV strain WIBP-MPXV-001. However, qPCR analysis revealed significant reductions in viral load within the lungs, ovaries, and spleens of vaccinated mice compared to those receiving QS-21, as well as in the lungs and ovaries when compared to the PBS-treated group. Although these findings are promising, further validation in a model more susceptible to MPXV infection and symptomatic disease is recommended.

During an investigation performed by Yang et al. (2023b), the MPXV antigens A29L, A35R, B6R, and M1R were successfully expressed in *E. coli* BL21 (DE3) cells and subsequently purified. In order to test their immunogenicity, these four recombinant proteins were combined in a mix called AMBA, which was administered to BALB/c mice alone, with aluminum hydroxide or with CpG7909 as adjuvants. Succeeding assays demonstrated that the immunization induced robust antigen-specific antibody production and a CD4^+^ T cell-mediated immune response. Additionally, virus neutralization assays confirmed that sera from immunized mice displayed high neutralizing activity against VACV. Notably, CpG7909 was found to be more effective than aluminum hydroxide in enhancing the immune response. Still, this vaccine should be tested in the future in an animal model subjected to viral challenge to verify its protective efficacy.

In another study carried out to evaluate the efficacy of a recombinant antigen vaccine, Wang et al. (2024) expressed a “two-in-one” immunogen in CHO cells, combining the MPXV antigens A35 from the EMV and M1 from the IMV. The experimental procedure involved immunizing BALB/c mice with three doses of alum-adjuvanted DAM. These mice were exposed to VACV WR to assess the vaccine’s efficacy. Remarkably, mice immunized with alum-adjuvanted DAM did not show significant weight loss compared to the control group, which experienced substantial weight loss and did not recover. In terms of survival, alum-adjuvanted DAM successfully provided full protection in the vaccinated group. Notably, increasing the concentration of DAM in mice resulted in stronger immune responses. This research indicates that the DAM vaccine could be a viable and safe alternative to the live VACV vaccine, as it produces higher levels of neutralizing antibodies and provides full protection against VACV.

Another recombinant antigen-based vaccine was evaluated by Li J. et al. (2024), who harnessed the B6R antigen of MPXV in combination with the BC02 adjuvant (B6R-BC02), which contains aluminum hydroxide and BC01 (BCG-CpG-DNA). To evaluate the ability of this vaccine to induce both cellular and humoral immune responses, BALB/c mice received two injections of B6R-BC02, leading to the production of MPXV-specific IgG, IgG1, and IgG2a antibodies. Additionally, it triggered a strong MPXV-specific Th1-oriented cellular immune response and persistent effector memory B-cell responses. This report also evidenced that BC02 enhances the activation of immune responses, promoting both rapid initiation and the long-term production of antibodies, along with sustained cellular immune responses. Despite these outcomes, the protective effect of the B6R-BC02 vaccine against a viral challenge in a reliable animal model for MPXV infection remains to be explored.

Yang et al. (2024) proposed another set of recombinant antigen-based vaccine candidates against MPXV, which comprised Mix-AE (A29 and E8), Mix-AEM (A29, E8, and M1), Mix-AEMA (A29, A35, E8, and M1), Mix-AEMB (A29, B6, E8, and M1), and Mix-AEMAB (A29, A35, B6, E8, and M1). The recombinant proteins of the vaccines were expressed in *E. coli* BL21 (DE3). Once BALB/c mice were immunized, the vaccines successfully induced strong neutralizing antibody responses and provided significant protection against VACV TT infection. Among these, Mix-AEM elicited notably higher levels of neutralizing antibodies, cellular immune response capacity, and virus clearance compared to Mix-AE. Immunization with single antigens showed that M1 induced greater neutralizing antibody levels than A29 and E8. These observations imply that M1 is a crucial and indispensable antigen for the design of new mpox vaccine candidates.

In an innovative methodology for producing next-generation mpox vaccines, Chen et al. (2025) used a reversible chemical cross-linking strategy to engineer protein antigens. The strategy was applied to the MPXV antigens A29L and A35R, creating antigen subunit vaccines in which each protein was cross-linked (i.e., A29L-CC and A35R-CC). Studies performed *in vivo* pointed out that the cross-linking strategy improved antigen delivery to lymph nodes and boosted both antigen-specific and virus-neutralizing antibody production. Subsequent experiments on vaccinated dormice that were challenged with the MPXV strain hMpxV/China/GZ8H-01/2023 that was obtained from a patient in Guangzhou, China showed that the engineered vaccines containing the two antigens reduced tissue damage, lowered viral load, and prolonged mouse survival, highlighting the potential of chemical cross-linking in protein-based subunit vaccine development. Particularly, immunization with a formulation containing A29L-CC, A35R-CC, and an alum adjuvant provided strong protection against MPXV-associated damage with a survival rate of 87.5%. This outcome also suggested that the combination of aluminum adjuvant and chemical cross-linked antigens generated a synergistic effect, enhancing the efficacy of the dual-antigen vaccine.

Likewise, in a recent vaccine proposal, Bai et al. (2025) engineered a chimeric A35R-Fc protein that included the Fc region of human IgG1 fused to the C-terminal of A35R. The results obtained by the authors demonstrated that A35R-Fc displayed a markedly higher binding affinity to A35R antibodies than a commercially available A35R protein and showed strong reactivity with human plasma. Furthermore, mice immunized with A35R-Fc developed significantly elevated neutralizing antibody titers against live MPXV. Given these promising results, it is crucial to evaluate this design in an animal model subjected to a viral challenge to confirm the vaccine’s protective efficacy.

### 3.5 Antibodies targeting MPXV antigens

In the search for innovative vaccine strategies, passive immunization has emerged as a promising yet underexplored approach. Instead of prompting the body to produce its own immune response, this strategy involves administering antibodies directly, thus offering immediate protection against infections, particularly in high-risk populations (Tharmalingam et al., 2022). These antibodies are often generated through recombinant approaches, allowing precise targeting of viral antigens. Platforms like CHO cells and plant expression systems are commonly used to produce these recombinant antibodies at a large scale (Pirkalkhoran et al., 2023). Alternatively, the hybridoma technique is also a common method for producing monoclonal antibodies. It involves fusing B lymphocytes from an immunized animal with myeloma cells, creating hybrid cells known as “hybridomas.” Once the desired antibody-secreting clones are identified, they are cultured to continuously proliferate, enabling large-scale production for clinical applications (Moraes et al., 2021). In spite of the potential of antibody-centered platforms for combating viral diseases, only a few studies have been conducted on the development of this type of immunotherapy against mpox.

A vaccine type based on passive immunization was developed by Li M. et al. (2023). After identifying that the monoclonal antibodies 3A1, 2D1, and 9F8 bind to A29L, they proceeded to produce those antibodies through hybridoma technology. The prophylactic and therapeutic efficacy of the antibodies was evaluated by administering monoclonal antibodies to mice and challenging them with VACV TT and VACV WR strains. The challenge occurred either 1 day after prophylactic administration (before infection) or following therapeutic administration (after infection). Interestingly, 9F8 displayed the highest neutralization activity and demonstrated full protective activity, while 3A1 and 2D1 showed partial protection in some groups, indicating that the antibody 9F8 could be considered in forthcoming immunotherapeutic trials against MPXV.

Zhao et al. (2024) investigated the use of monoclonal antibodies to target specific antigens of MPXV. Accordingly, the immunotherapeutic approaches tested were hMB621 and hMB668, which are antibodies that target the MPXV B6 antigen. These antibodies were administered to BALB/c mice, which were later challenged with VACV WR, followed by a second antibody dose. Neutralization tests indicated that both hMB621 and hMB668 have remarkable neutralizing activity against VACV. In terms of efficacy, mice control groups experienced significant weight loss and did not recover. In contrast, mice treated with hMB621 and hMB668 showed weight gain from day four post-infection and all of them survived the viral challenge. These outcomes indicate that both antibodies display significant neutralization activity and are promising candidates against MPXV.

In another investigation, three monoclonal antibodies with neutralizing activity were identified, M1H11 and M3B2, both targeting M1R, and B7C9, which specifically binds to B6R. Further experiments indicated that a combination of M1H11 and M3B2 exhibited superior protective effects in BALB/c mice. To further optimize this response, a bispecific antibody named Bis-M1M3 was engineered by conjugating the Fc region of the human-mouse chimeric M1H11 with the scFv fragment of M3B2. In BALB/c mice challenged with MPXV, Bis-M1M3 provided remarkable protection. Additional analyses demonstrated that the monoclonal antibodies and Bis-M1M3 exerted virus-neutralizing effects before viral entry into host cells. Additionally, pharmacokinetic studies in rhesus macaques revealed that Bis-M1M3 has an extended half-life (5.142 days), highlighting its potential as a therapeutic agent (Ren et al., 2024).

### 3.6 circRNA-based vaccine encoding MPXV antigens

In the evolving landscape of RNA-based vaccines, a new contender is emerging with the potential to overcome some of the limitations faced by traditional mRNA approaches. For instance, mRNAs are susceptible to degradation, which can limit the duration of protein expression. circRNAs, unlike their linear counterparts, form a closed-loop structure, making them far more resistant to enzymatic degradation (Liu et al., 2022). This enhanced stability leads to longer-lasting protein production, potentially reducing the dose required by circRNAs to trigger immune responses. Nonetheless, additional analyses are required to ascertain the safety of this type of vaccine (Bai et al., 2023).

In this context, circRNA-based vaccines were developed by (Zhou et al., 2024) to induce immune responses via the surface proteins A29L, A35R, B6R and M1R from MPXV. The antigens were encapsulated in LNPs to create four different types of vaccines: cirA29L, ciA35R, cirB6R, and cirM1R, as well as a mixture of the four antigens called cirMix4. Subsequently, BALB/c mice were immunized twice with either individual circRNAs or with the multi-antigen formulation, followed by a challenge with VACV TT. Mice that received a placebo or circA29L showed a significant weight loss and died after several days, while the other groups gradually recovered, with cirMix4 and circM1R demonstrating the quickest recovery. Mice vaccinated with circA35R and circM1R had fewer viral particles in their examined tissues compared to the placebo group. Moreover, all the vaccines except for circA29L showed complete protection against VACV, ensuring the survival of the immunized mice. Intriguingly, the cirMix4 vaccine induced high neutralizing antibodies against VACV and MPXV and also demonstrated the potential for robust cellular immunity, indicating that this vaccine could provide effective protection against MPXV.

**FIGURE 3:**
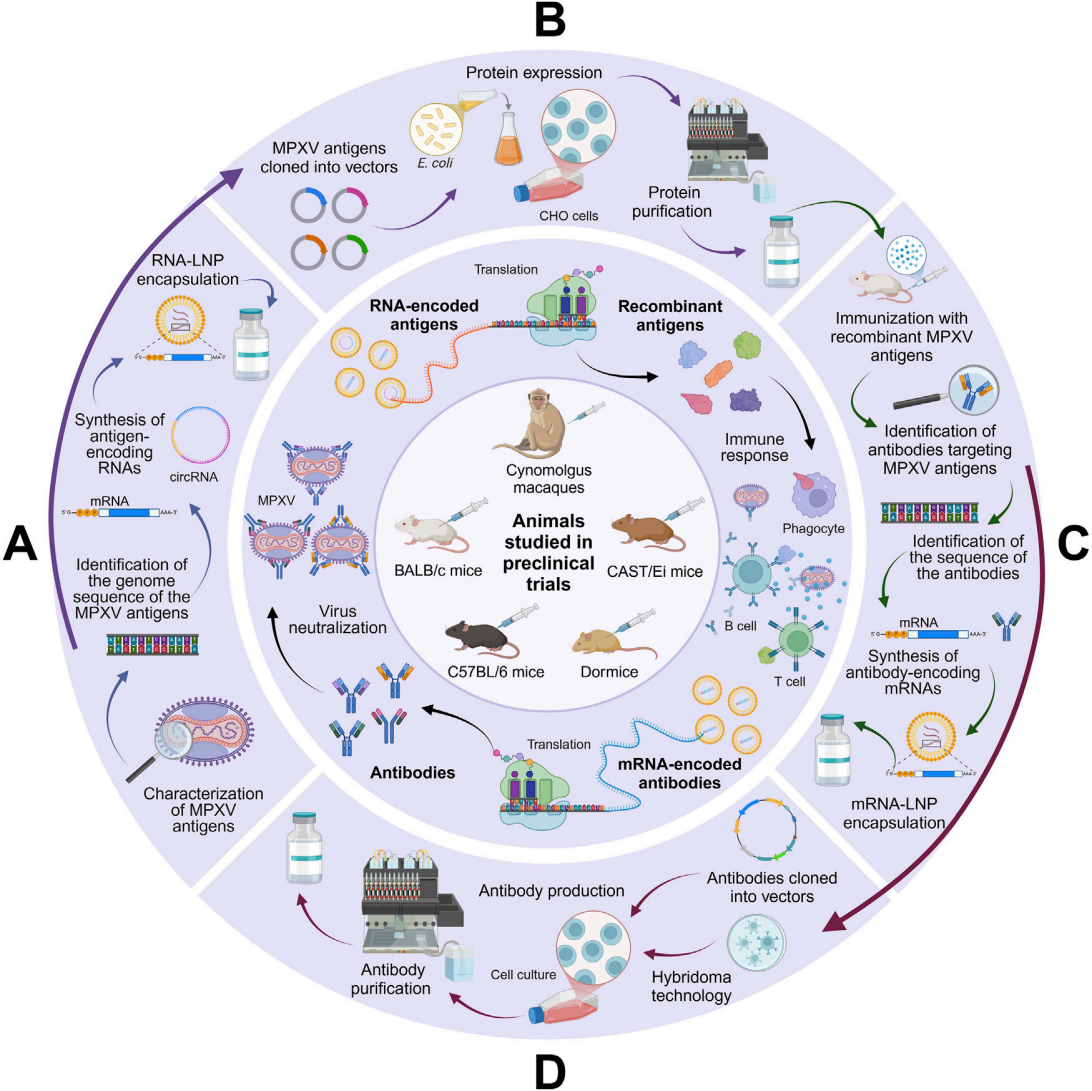
Overview of the mechanisms of new generation vaccines against MPXV. **(A)** mRNA and circRNA vaccines function by encoding MPXV antigens (e.g., A29L, A35R, B6R, E8L, and/or M1R), which trigger immune responses that prepare the system against the virus. **(B)** Recombinant protein vaccines, on the other hand, deliver specific antigens produced in expression platforms like *Escherichia coli* or CHO cells, directly stimulating immune responses without the need for cellular transcription. **(C)** mRNA-based antibody vaccines encode antibodies that target MPXV antigens, enabling the body to produce them internally and offering a faster protective alternative to conventional active immunization through the neutralization of the virus. **(D)** Similarly, anti-MPXV antibody vaccines work by administering antibodies produced in mammalian cells (using either recombinant or hybridoma technology), providing immediate immunity without requiring the host to produce its own antibodies (created with a licensed version of BioRender.com).

A visual summary of the different types of immunogenic approaches targeting MPXV discussed in this manuscript is depicted in Figure 3.

### 3.7 Risk of bias across studies

**FIGURE 4:**
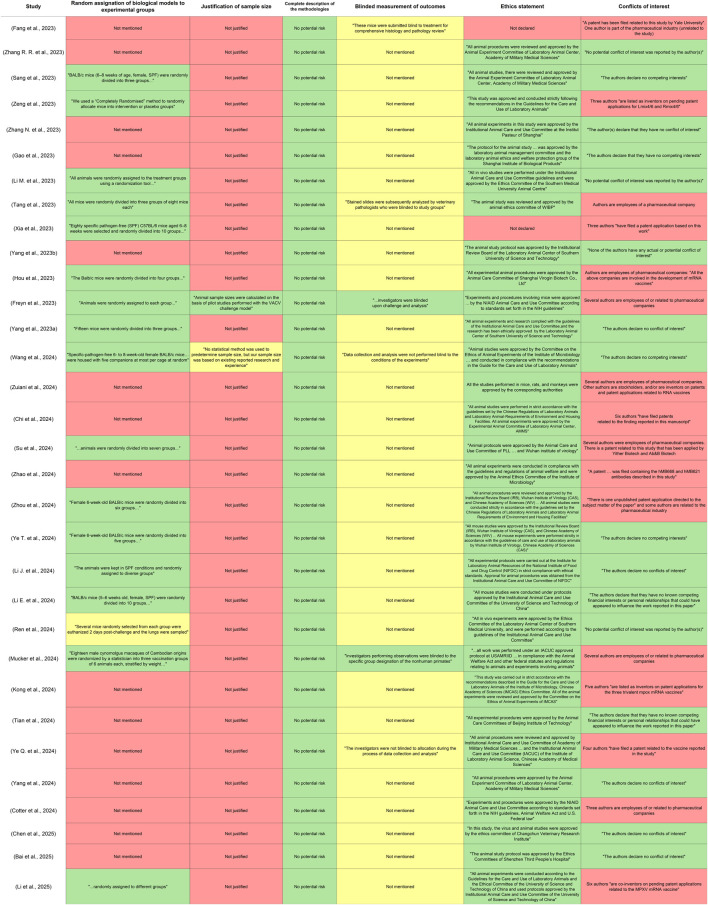
Results of the risk of bias assessment. The domains were evaluated for each included article, considering criteria for preclinical studies. Quotations extracted from the reports were included within the assessment to justify the risk assigned to each of the domains. The cells in green color represent low risk, yellow color unclear risk, and red color high risk.

The risks of bias in the studies involved that 16 reports did not mention randomization for generating experimental groups. Only one study provided a detailed explanation of its experimental design and justified its group sizes. Additionally, only two reports explicitly stated that the researchers performed a blinded measurement of the outcomes, particularly during viral challenges. Regarding the disclosure of ethical statements, two studies failed to present this information within the text of the article. Finally, many authors had affiliations with the pharmaceutical industry or had patent applications related to their findings or other vaccines. This assessment is presented in Figure 4, which includes relevant quotations from the included studies to justify the levels of risk assigned.

### 3.8 Patents of next-generation vaccines against MPXV

In the current inquiry, only 4 patents were found published between 2018 and 2024 and related to the development of next-generation vaccines exclusively against MPXV. All of them were issued under the registry of the World Intellectual Property Organization. China currently holds the highest number of patents in this field (2), followed by Germany (1) and USA (1). Despite the low number found, the quantity of patents in this field is expected to increase in the coming years since several of the reports on new generation mpox vaccines discussed herein declared the application of patents in their conflicts of interest (see Risk of bias across studies, Figure 4). Notably, all the patents examined in this review are directly associated with vaccine designs detailed in some of the analyzed studies. This connection underscores that these patents are not standalone claims but rather tangible products of the scientific breakthroughs documented in peer-reviewed research. As a result, they serve as clear indicators of the accelerated technological innovation in this field and reflect the urgency and global priority placed on mpox vaccine development. The key aspects of these patents are shown in Table 1.

**TABLE 1:** Summary of patents centered on vaccines against MPXV.

Title	Authors and publication year	Application country and company or institution	Type of vaccine	Targeted antigens	Country or office	Patent number
RNA compositions for delivery of monkeypox antigens and related methods	Poran et al. (2023)	Germany, BioNTech SE	RNA-based vaccines or recombinant antigens	A29L, A35R, B6R, MIR, E8L, A28L, H3L, A45L, B9R, B16R, C10L, C21L, E7R, F3L, F4L, G6R, H5R, I3L, O2L, Q1L, B12R, and C17L	World Intellectual Property Organization	WO2023230295
Multi-antigen chimeric poxvirus vaccine and use thereof	Gao et al. (2024a)	China, Institute of Microbiology, Chinese Academy of Sciences	Recombinant antigens of the MPXV	A35R, B6R, and M1R	World Intellectual Property Organization	WO2024114542
Monkeypox virus nucleic acid vaccine and use thereof	Gao et al. (2024b)	China, Institute of Microbiology, Chinese Academy of Sciences	mRNA-based vaccine	A35R and M1R	World Intellectual Property Organization	WO2024152870
Compositions and methods for enhancement of mRNA vaccine performance and vaccination against mpox	Chen and Fang (2024)	United States, Yale University	mRNA-based vaccine	A29L, A35R, B6R, E8L, and M1R	World Intellectual Property Organization	WO2024263202

## 4 Discussion

The results of this scoping review indicate that mRNA-based vaccines encoding MPXV antigens elicit the production of antibodies that aid in virus clearance, induce immune cell responses, and protect animal models from viral challenge, thus representing potential approaches to combat mpox. These observations are congruent with the previous key role played by mRNA-based vaccines in mitigating the COVID-19 pandemic (Iqbal et al., 2024). However, a few other studies included in this analysis suggest that recombinant MPXV antigens, antibodies targeting MPXV antigens, and circRNAs encoding MPXV antigens also hold potential as alternatives to prevent and contend this emerging virus. Therefore, these types of vaccine formulations should not be underestimated for future exploration.

Particularly, the application of antibodies in mpox prevention warrants further exploration, as this strategy could be helpful in cases where individuals necessitate urgent protection or are incapable of promptly generating antibodies, such as newborns, older adults, or patients with preexisting immunodeficiencies (Perricone et al., 2021; Verwey and Madhi, 2023). Moreover, it is important to acknowledge that the obstacles related to large-scale synthesis and purification of recombinant antibodies can be overcome due to recent advancements in the generation of mRNAs that can encode complex antibody structures upon delivery to the organism (Deal et al., 2021; Zhao et al., 2023). In fact, the completion of phase I clinical trials of mRNA-1944 (NCT03829384), a mRNA-LNP encoding an antibody (CHKV-24) targeting the Chikungunya virus (August et al., 2021), might be a great promise for mRNA-based antibodies to reach the clinical landscape. In parallel, the efficacy of the mRNA-encoded antibodies against MPXV (Chi et al., 2024) should be studied in more depth in future investigations.

This review also indicates the limitations of the included studies. For instance, a number of experiments (18 out of 32 studies) solely used VACV strains, which are safer to handle (Peng et al., 2023; Sharma et al., 2023), as the viral challenge to test the protection conferred by the vaccines rather than MPXV itself. Although VACV can serve as the platform for a range of vaccines targeting orthopoxviruses (Shchelkunova and Shchelkunov, 2023; Shchelkunov et al., 2024), the existence of genomic and structural differences between VACV and MPXV affect the level of virulence exhibited by each virus (Weaver and Isaacs, 2008; Forni et al., 2022; Shen-Gunther et al., 2025). Importantly, the low virulence of VACV was shown in some studies, where placebo-vaccinated controls survived despite not receiving prior immunization with the tested vaccines. Accordingly, this factor may introduce potential reproducibility risks in the studies regarding the actual capacity of these vaccines to protect against MPXV infection, and future studies should consider using MPXV strains as the viral challenge for testing vaccine efficacy. Indeed, a recent systematic review (Eslami et al., 2024) focused on first, second, third, and fourth-generation vaccines (platforms not centered on the MPXV itself) and excluded articles that did not use MPXV as a challenge to test the immunogenic effects of the vaccines.

Besides, the efficacy of new generation vaccines against the different MPXV clades must be evaluated in the outlook since the virulence of this virus also differs between clades, with MPXV clade I being the most virulent of all, followed by clade IIa and clade IIb (Americo et al., 2023; Okwor et al., 2023). While not the main focus of the respective reports, some researchers designed their vaccines using sequences of MPXV isolates different from those used in the viral challenge and those vaccines provided protection against the viral challenge (Kong et al., 2024; Chen et al., 2025). This suggests that these vaccines could potentially provide cross-protection between different isolates, although this fact was not concluded in the corresponding studies. Nevertheless, it is also crucial that future mpox vaccine designs incorporate antigens from multiple MPXV clades rather than focusing solely on one clade. Although some new generation mpox vaccine designs were labeled as “multivalent/polyvalent vaccines” by the authors of the respective studies, this terminology is not completely appropriate as multivalent vaccines are focused on targeting multiple variant strains or serotypes of a virus (Lauer et al., 2017; Sanyal, 2022; Hou et al., 2024). On the contrary, several current next-generation mpox vaccine candidates were tailored to target multiple antigens of a single clade, potentially limiting their cross-protective efficacy. Consequently, it is mandatory that upcoming investigations on new generation mpox vaccines consider that optimal vaccine designs should aim to confer robust protection across the diverse clades of MPXV, similar to what has been previously done with vaccines against other viruses, such as Bimervax (PHH-1V), which targets the alpha and beta variants of SARS-CoV-2 (Leal et al., 2023; England et al., 2024), as well as the bivalent oral polio vaccine that targets types 1 and 3 polioviruses (Sutter et al., 2010; Farrell et al., 2017).

In addition to the previous limitations, several investigations relied only on BALB/c mice as the biological model; in fact, only three studies assessed vaccine effectiveness in non-human primates (Mucker et al., 2024; Ye Q. et al., 2024; Zuiani et al., 2024). Although CAST/Ei mice are an appropriate model for orthopoxvirus-related studies (Americo et al., 2014; Earl et al., 2015), prairie dogs, rhesus macaques, and cynomolgus macaques are considered the most suitable animal models for studying the efficacy of mpox vaccines, as they exhibit a disease profile that resembles the one observed in humans (Guarner et al., 2004; Aid et al., 2023; Falendysz et al., 2023; Li Q. et al., 2024). However, despite their relevance, working with non-human primate species can be more expensive and limit the size of experimental groups, which can reduce statistical significance (Wei et al., 2023). For this reason, it is understandable that most of the studies analyzed in this scoping review utilized more accessible models, such as BALB/c mice, CAST/Ei mice, and C57BL/6 mice. Interestingly, despite the fact that BALB/c mice do not present susceptibility to MPXV infection (Shang et al., 2025), observations reported by Cheng et al. (2024) suggest that BALB/c mice infected with clade IIb MPXV strain SZTH42 may represent a suitable infection model for mpox-related, as the infection with that strain induced a clear pathogenic phenotype in the mice. Nevertheless, in the future, those vaccines tested only in mice that yielded promising results will need to be further evaluated in non-human primates.

Additionally, no animal model will perfectly replicate the full spectrum of symptoms seen in human infection (Wei et al., 2023), and the selection of the animal model will depend on the specific question pursued by each investigation (Rosa et al., 2023). Therefore, it is advisable to assess vaccines in various animal models, integrating key data from each model to obtain a clearer perspective that can guide advancements in human clinical trials. This clinical timeline is exemplified by Zuiani et al. (2024), who evaluated the protective effects of the BNT166a vaccine in CAST/Ei mice, BALB/c mice, and cynomolgus macaques to gain a broader understanding of the effects of BNT166a in different animal models challenged. Interestingly, BNT166a is one of the first next-generation vaccines exclusively designed for MPXV registered at ClinicalTrials.gov (NCT05988203) for a phase I/II trial aiming to evaluate the safety and immune responses of this immunogenic approach. Similarly, the mRNA-based vaccine named mRNA-1769, reported by Mucker et al. (2024) and Cotter et al. (2024), which was highlighted by Mayer et al. (2024), has already been registered (NCT05995275) for a phase I/II study which intends to assess the safety and immunogenicity of the vaccine in healthy subjects.

Another major constraint of preclinical studies on mpox vaccines is related to the small group sizes considered in the experimental designs. This is primarily due to ethical principles that restrict the excessive use of animals in experimental designs. While these ethical guidelines are crucial to minimizing animal suffering, they can lead to lower validity of the inferences made in a particular investigation (Konietschke et al., 2021). Accordingly, findings from these reports must be interpreted with caution before advancing to the next clinical phases, as their limited group sizes may impact the statistical robustness and generalizability of the outcomes. It is advisable that forthcoming preclinical studies on mpox vaccines consider other approaches within their experimental designs that allow them to obtain significant data with ethically and statistically acceptable group sizes. For example, using data from control groups of previous studies can enhance the power of these experiments through Bayesian priors and reduce the need for large groups in the new study (Bonapersona et al., 2021). The number of animal models used in experiments could also be reduced while increasing reproducibility by conducting mini-experiments, in which the originally considered group size is divided into smaller groups and tested at different time points. For instance, if a conventional design called for nine animal replicates in a single experiment, this number could be split into three mini-experiments with three biological replicates conducted at different times (von Kortzfleisch et al., 2020). This can introduce variation in external factors across the experiments, potentially helping to validate whether the outcomes are reproducible, at least at the intra-laboratory level. In any case, group size calculation or justification must be properly reported to avoid risks of bias (Avey et al., 2016). Noticeably, only one article on next-generation mpox vaccines justified the value of the sample sizes (Freyn et al., 2023).

Over-standardization of experimental setups often results in irreproducibility, so introducing more variability across vaccine testing may help ensure that findings are robust and applicable in diverse settings (Karp, 2018). Thus, there should also be an emphasis on conducting multi-laboratory studies for mpox vaccine development, as this can contribute to increasing the heterogeneity in experiments and validate their reproducibility (Voelkl et al., 2018). Additionally, integrating a broad genetic diversity in animal models and varying co-housing laboratory conditions is crucial for validation (Voelkl et al., 2020). Contrary to this, certain parameters should indeed be standardized within mpox vaccine evaluations to allow for comparisons between their outcome measures. These include the lethal dose of VACV or MPXV used during viral challenges and the route of administration, as these factors influence the severity of the MPXV infection manifested by the biological models (Wei et al., 2023). Notwithstanding this, the administration routes and doses used in the existing literature are highly heterogeneous, often depending on the viral strains and biological model. Indeed, only one article investigated the effect of the route of infection on the efficacy of the mpox vaccine (Cotter et al., 2024). To address this, we recommend conducting a specialized review of these factors to identify the most suitable doses and administration routes for each animal model and strain of VACV or MPXV.

Evaluating the long-term protection of mpox next-generation vaccines is crucial for shaping effective vaccination policies, especially as immunity can vary greatly across different vaccines. Some vaccines offer lifelong protection, while others require frequent boosters to maintain efficacy (Vashishtha and Kumar, 2024). Understanding the duration of immunity provided by mpox vaccines is essential, as this data informs public health strategies, particularly for vulnerable populations such as the elderly (Vidor, 2010). In this regard, while handful reports on new generation mpox vaccines indicated the existence of long-term immunity based on sustained cellular immune responses or antibody titers across several weeks or months (Li J. et al., 2024; Su et al., 2024; Yang et al., 2024; Ye Q. et al., 2024), some other experiments also provided information about long-lasting protection, being 65 days (Zhang N. et al., 2023), 91 days (Zhang R. R. et al., 2023), 16 weeks/112 days (Cotter et al., 2024), 162 days (Hou et al., 2023), 190 days (Kong et al., 2024), and 280 days (Li et al., 2025) after the first immunization, the longest times considered to perform the viral challenge. Consistently, mpox vaccine-related preclinical studies and clinical trials should focus not only on immediate efficacy but also on the persistence of immunity to ensure that long-term protection can be achieved with these vaccines.

Likewise, the pharmacodynamics, pharmacokinetics, and safety of mpox vaccines must be thoroughly explored. In spite of the fact that numerous investigations sustained the safety of their respective next-generation mpox vaccine designs through biochemical and histological analyses (Fang et al., 2023; Sang et al., 2023; Xia et al., 2023; Li E. et al., 2024; Tian et al., 2024; Wang et al., 2024), each type of mpox vaccine candidate might induce particular adverse events in humans following immunization. For mRNA vaccines, the lipid formulation can promote the entry of the mRNA into potentially any cell in the body (Federico, 2022), and it has even been suggested that it could integrate into the host DNA (Domazet-Lošo, 2022; Acevedo-Whitehouse and Bruno, 2023). Myocarditis and pericarditis have also been broadly documented in individuals who received mRNA COVID-19 vaccines (Lane et al., 2022), while chest pain is one of the most reported adverse effects for Nuvaxovid, a protein-based vaccine for COVID-19 (Clothier et al., 2024). On the other hand, adverse events for the antibody bamlanivimab for COVID-19 were generally mild and well-tolerated, being nausea and diarrhea among the most common events (Amani et al., 2024). Likewise, most of the adverse effects reported for the mRNA-1944 vaccine against Chikungunya virus were ephemeral and self-resolving, requiring no therapeutic intervention (August et al., 2021). Additionally, the use of recombinant antigen proteins raises questions, as the interaction between these proteins and the antibodies formed during the immune response can significantly alter their pharmacodynamics and pharmacokinetics (Mahmood and Green, 2005). Only clinical studies will provide a clearer understanding of how all these vaccines interact with the human body. Therefore, we emphasize the need for continued testing of next-generation mpox vaccines in the coming years.

On the other hand, some MPXV proteins, such as VP37, which plays a critical role in the formation of the enveloped virus (Huo et al., 2024), have not yet been thoroughly investigated as potential vaccine targets. Moreover, the functions of many MPXV hypothetical proteins remain unvalidated through experimental methods (Gupta, 2023; Raen et al., 2024). Consequently, a deeper exploration of these proteins could facilitate the identification of novel vaccine candidates for mpox. In this context, we believe that bioinformatics studies also offer a rich source of information for potential designs of next-generation mpox vaccines. Although these studies were excluded from this scoping review, the bioinformatic-related reports identified through our search methodology can be found in Supplementary Table S3.

The growing importance of developing new generation mpox vaccines has also driven the exploration of innovative strategies to enhance immune responses through adjuvant-based strategies. One such approach is the programmable self-adjuvant bionic vaccine based on macrophage-derived vesicles (AM@AEvs-PB), which facilitates efficient antigen presentation. This system incorporates MPXV antigens A29L, B6R, and M1R, leveraging activated macrophage vesicles to improve immune activation and protect from viral challenge (Lin et al., 2025). Besides, it has been discovered that the self-assembling lipopeptide C16-GCV_2_E_3_ can enhance immune responses when delivered with recombinant MPXV antigens (Bao et al., 2025). Thus, integrating these innovations with existing next-generation mpox vaccine candidates and future vaccine designs could significantly improve efforts to combat mpox.

**FIGURE 5:**
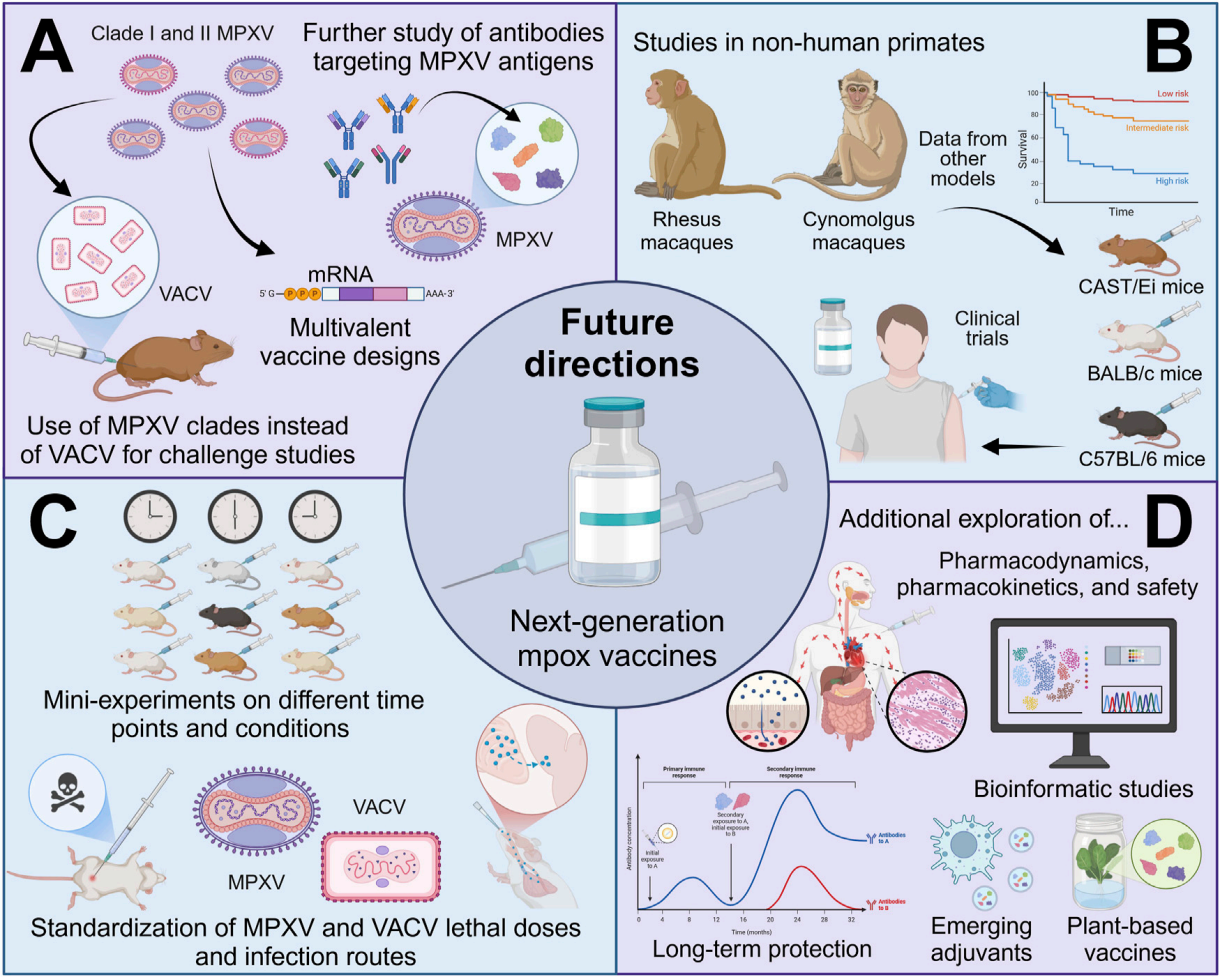
Future directions for next-generation mpox vaccine development. **(A)** Forthcoming investigations should explore more in-depth antibody-based strategies, multivalent vaccines targeting multiple clades of MPXV, and the utilization of MPXV, rather than VACV, for challenge studies, incorporating diverse MPXV clades for comprehensive analysis. **(B)** The use of animal models that better replicate human disease complexity, such as non-human primates, along with the integration of data from other animal models, can support more robust analyses of mpox vaccine efficacy. **(C)** Ensuring adequate group sizes and avoiding over-standardization of experimental conditions will be key for validating reproducibility while standardizing factors such as lethal doses and virus administration routes tailored to the biological model is necessary. **(D)** Additional focus on pharmacodynamics, pharmacokinetics, safety, long-term protection, bioinformatic studies, emerging adjuvants (e.g., programable macrophage-derived vesicles) and plant-based vaccine development will also contribute to advancing mpox vaccine research (created with a licensed version of BioRender.com).

Notably, to date, there are no plant-based vaccine candidates for mpox. This implies that there is a valuable opportunity and a clear path for future studies to explore innovative immunization strategies against this virus. Importantly, the potential of plant-derived immunotherapies to prevent and manage other viruses (El Jaddaoui et al., 2022; Su et al., 2023), cancers (Rahimian et al., 2021), neurodegenerative diseases (Bravo-Vázquez et al., 2023), among several other human ailments, has been widely explored. Plant-based vaccines offer several advantages, including cost-effectiveness, ease of large-scale production, non-invasive administration as plants or leaves can serve as the delivery system, and improved safety profiles due to their lower risk of contamination with animal or human pathogens (Rosales-Mendoza et al., 2020; Ndayambaje et al., 2025). Thus, it is strongly recommended that future studies focus on the production of MPXV antigens with widely characterized immunogenic properties in plant-based expression platforms, as these approaches could ultimately improve the accessibility and global reach of mpox vaccination. The suggested future directions for upcoming studies in this field are illustrated in Figure 5.

Our scoping review possesses several strengths. The study employed a thorough and transparent methodology, incorporating a comprehensive literature search through multiple databases. The search approach was meticulously adjusted several times prior to the final article extraction to guarantee comprehensiveness. Indeed, following the strict PRISMA guidelines in this study could have provided a great reduction in the risk of bias associated with human inaccuracies. Some limitations of our work include the possibility of missing articles published before the selected date range of 2018–2024. Nonetheless, since the first included article chronologically was from 2023, the risk of excluding earlier studies is likely low. As well, we further complemented the content of this scoping review by mentioning some significant pioneering reports in this field within the Introduction section.

Another limitation is that all the data came from preclinical studies with high heterogeneity across different aspects such as experimental designs, animal models, immunization schedules, placebos, strains used in the viral challenges, and follow-up times after the challenges. Even though we handled this heterogeneity by grouping studies according to vaccine type, similar to an existing vaccine-related systematic review (Hashemi et al., 2023), the interpretation of the findings discussed herein should be approached with caution as the variability between investigations may influence the overall conclusion on the real efficacy of each class of new generation mpox vaccine. To gain a deeper understanding of the heterogeneity among studies, of vaccine efficacy, and integrate these insights into final conclusions, additional data from both preclinical and ongoing clinical studies are needed. This expanded dataset would enable the elaboration of reviews that incorporate statistical assessments through meta-analysis, as previously evidenced by Gross et al. (2018). Finally, limiting the inclusion criteria to English-language articles may have excluded studies in other languages. Anyhow, few records in other languages were detected and the final conclusions would likely remain largely unchanged even if these studies were included.

The findings reported thus far on next-generation mpox vaccines, particularly those based on mRNA and recombinant antigens, indicate a strong trend toward conferring high protection against mpox disease. This is particularly evident in the high survival rates observed in animal models challenged. These findings are consistent with efficacy estimates for mRNA and protein subunit vaccines against SARS-CoV-2, which have demonstrated over 90% effectiveness in preventing disease in individuals over 18 years old (Sandoval et al., 2023). However, as previously discussed, many of the studies utilized VACV as the challenge virus, which is less virulent than MPXV and poses a lower risk to biological models. On the other hand, some studies challenged BALB/c mice with MPXV, which is not entirely correct since BALB/c mice are generally not susceptible to MPXV infection. Consequently, these findings may not be fully conclusive. Still, studies that employed MPXV for the challenge in appropriate animal models continue to support the notion that mRNA and recombinant antigen vaccines could achieve high efficacy against mpox.

For other immunotherapeutic platforms, such as antibodies and circRNA-based vaccines, the current body of research remains limited, preventing definitive conclusions about their efficacy. Anyhow, the available data suggest that these immunotherapies may achieve efficacy levels comparable to those of mRNA and recombinant antigen-based vaccines. Once clinical study data become available, we recommend conducting a systematic review to compare the efficacy of each vaccine platform in disease prevention. Therefore, this scoping review should serve as a precursor for future systematic reviews with meta-analyses, enabling the generation of statistically substantiated conclusions regarding the efficacy of different next-generation mpox vaccine candidates.

Overall, this research provides practical insights for clinical virologists to continue exploring the effects of the best antigen combinations in multi-antigen vaccines to prevent and manage mpox disease. In general, vaccines that incorporated multiple antigens in their design provided greater protection against viral challenges, resulting in higher survival rates. This suggests that targeting a broader range of viral components can enhance immune responses and improve overall vaccine efficacy. Notwithstanding this, we emphasize the necessity of developing multivalent vaccine designs that offer broader protection against the diverse clades of MPXV. Additionally, this work underscores the critical importance of international and interdisciplinary collaboration in the development of effective vaccines to prevent an mpox pandemic. From a policy perspective, this investigation also highlights that investments from non-governmental organizations and the pharmaceutical industry are critical nowadays to drive the development of innovative vaccine platforms, such as mRNA-based vaccines, recombinant antigens, or antibody-based passive immunizations. These collective efforts will be essential in addressing emerging health threats and ensuring global readiness against potential mpox outbreaks.

## Data Availability

The data that support the findings of this study are available from the corresponding author upon reasonable request or in the article/Supplementary Material.
