# Supplementary material for: Current status of next-generation vaccines against mpox virus: a scoping review
Source: Front Pharmacol. 2025 Apr 28;16:1533533. doi: 10.3389/fphar.2025.1533533 (PMC12066571; doi:10.3389/fphar.2025.1533533)
Supplement: Supplementary file 1 [file Supplementaryfile1.docx]

**SUPPLEMENTARY TABLE S1** **Information about the searches performed in the 11 databases consulted in this scoping review.**

| Name of the database | Search strings used in the database as appeared after the search | Dates of coverage allowed by the database | Date of the search |
| --- | --- | --- | --- |
| Scopus | ABS((monkeypox OR mpox) AND (virus OR disease OR infection) AND (vaccine OR immunization) AND (mRNA OR DNA OR epitope)) | From 2018 to 2024 | 22/08/2024 |
| MEDLINE  (ProQuest) | abstract(monkeypox OR mpox) AND abstract(virus OR disease OR infection) AND abstract(vaccine OR immunization) AND abstract(mRNA OR DNA OR epitope) | From 22-08-2018 to 22-08-2024 | 22/08/2024 |
| Web of Science | (monkeypox OR mpox) (Abstract) AND (virus OR disease OR infection) (Abstract) AND (vaccine OR immunization) (Abstract) AND (mRNA OR DNA OR epitope) (Abstract) | From 2018-08-22 to 2024-08-22 | 22/08/2024 |
| PubMed | (monkeypox[Title/Abstract] OR mpox[Title/Abstract]) AND (virus[Title/Abstract] OR disease[Title/Abstract] OR infection[Title/Abstract]) AND (vaccine[Title/Abstract] OR immunization[Title/Abstract]) AND (mRNA[Title/Abstract] OR DNA[Title/Abstract] OR epitope[Title/Abstract]) | From 2018-08-22 to 2024-08-22 | 22/08/2024 |
| Academic Search Ultimate (EBSCOhost) | AB (monkeypox OR mpox) AND AB (virus OR disease OR infection) AND AB (vaccine OR immunization) AND AB (mRNA OR DNA OR epitope) | From 22/08/2018 to 22/08/2024 | 22/08/2024 |
| SpringerLink Journals | AB((monkeypox OR mpox) AND (virus OR disease OR infection) AND (vaccine OR immunization) AND (mRNA OR DNA OR epitope))  Only the "Article" entry was selected in the "Content Type" options | From 2018 to 2024 | 22/08/2024 |
| Gale Academic OneFile | Abstract: (monkeypox OR mpox) AND Abstract: (virus OR disease OR infection) AND Abstract: (vaccine OR immunization) AND Abstract: (mRNA OR DNA OR epitope) | From 22-08-2018 to 22-08-2024 | 22/08/2024 |
| Taylor & Francis Journals | [[Abstract: monkeypox] OR [Abstract: mpox]] AND [[Abstract: virus] OR [Abstract: disease] OR [Abstract: infection]] AND [[Abstract: vaccine] OR [Abstract: immunization]] AND [[Abstract: mrna] OR [Abstract: dna] OR [Abstract: epitope]] AND [Publication Date: (01/01/2018 TO 12/31/2024)] | From 2018 to 2024 (01/01/2018 to 12/31/2024) | 22/08/2024 |
| Wiley Online Library | "monkeypox OR mpox" in Abstract and "virus OR disease OR infection" in Abstract and "vaccine OR immunization" in Abstract and "mRNA OR DNA OR epitope" in Abstract | From 08-2018 to 08-2024 | 22/08/2024 |
| Directory of Open Access Journals (DOAJ) | (monkeypox OR mpox) AND (virus OR disease OR infection) AND (vaccine OR immunization) AND (mRNA OR DNA OR epitope)  Searched in the "Abstract" option | From 2018 to 2024 | 22/08/2024 |
| PATENTSCOPE (WIPO) | FP:(monkeypox vaccine) | The patents were manually selected from 2018 to 2024 | 22/08/2024 |

**SUPPLEMENTARY TABLE S2 Summary of the included articles reporting the development of next-generation vaccines against MPXV.**

| Authors and publication year | Biological model | Type of vaccine | Targeted MPXV clade or isolate | Targeted antigens | Immunization scheme | Placebo | Viral strain and dose | Monitoring time after the challenge | Survival rate |
| --- | --- | --- | --- | --- | --- | --- | --- | --- | --- |
| (Fang et al., 2023) | Mice (strain not reported) | mRNA-LNP vaccine | USA, 2022 (isolate or accession no. not reported) | MPXVac-097: A29L, A35R, B6R, E8L, and M1R antigens in tandem joint by the 2A peptide  Mix-5: A29L, A35R, B6R, E8L, and M1R mRNA-LNPs | Doses of 5 μg for both MPXVac-097 and Mix-5 (1 μg of each mRNA-LNP)  Prime: Day 0  Boost 1: Day 7  Boost 2: Day 14  Challenge: Day 28 | Mice injected with PBS, but not challenged and unvaccinated mice were the control groups | VACV: strain and dose not reported (intranasally) | 11 days | All mice survived the viral challenge, including PBS-treated and unvaccinated controls, which did not show more than 10% weight loss.* |
| (Zhang et al., 2023b) | BALB/c mice | mRNA-LNP vaccine | MPXV isolate MPXV_USA_2022_MA001 (ON563414.2) | AR-MPXV3: A29L, B6R, and E8L  AR-MPXV4a: A29L, B6R, E8L, and M1R  AR-MPXV4b: A29L, A35R, B6R, and E8L  AR-MPXV5: A29L, A35R, B6R, E8L, and M1R | The mixtures contained doses of 5 μg of each mRNA-LNP  Prime: Day 0  Boost: Day 21  Challenge: Day 91 | Empty LNPs | VACV TT: 1×10^6^ PFU (intranasally) | 20 days | All mice survived the viral challenge, including the empty LNP-treated controls, which showed weight recovery.* |
| (Sang et al., 2023) | BALB/c mice | mRNA-LNP vaccine | Not reported | mRNA-A-LNP: A29L, A35R, B6R, and M1R  mRNA-B-LNP: A29L, A35R, B6R, and M1R | Doses of 40 μg  Prime: Day 0  Boost: Day 14  Challenge: Day 30 | Naive mice were the control group | VACV TT: 4×10^5^ median Tissue Culture Infectious Dose (TCID_50_, subcutaneously) | NA | Not assessed with the challenged mice. |
| (Zeng et al., 2023) | BALB/c mice | mRNA-LNP vaccine | Not reported | L/Rmix4: A29, A35, B6, and M1  L/Rmix6: A29, A35, B6, E8, H3, and M1 | Doses of 1 μg or 5 μg  Prime: Day 0  Boost: Day 14  Challenge: Day 28 | PBS | VACV WR: 5×10^6^ PFU (intraperitoneally) | 14 days | All vaccinated mice survived the viral challenge, while 83.3% (5/6) of the PBS-treated controls died. |
| (Zhang et al., 2023a) | BALB/c mice | mRNA-LNP vaccine | MPXV isolate MPXV-M5312_HM12_Rivers  (GCF_014621545.1) | MPV-E2: A35R and B6R  MPV-M2: H3L and M1R  MPV-M4: A29L, E8L, H3L, and M1R  MPV-EM6: A29L, A35R, B6R, E8L, H3L, and M1R | Doses of 7.5 μg  Prime: Day 0  Boost: Day 14  Challenge: Day 65 | Non-coding mRNA | VACV TT: 1×10^6^ PFU (intranasally) | 19 days | All vaccinated mice survived the viral challenge, while all the non-coding mRNA-treated controls were sacrificed after excessive weight loss.* |
| (Gao et al., 2023) | BALB/c mice | Recombinant antigens of the MPXV | Not reported | A29, A35, B6, H3, I1, and M1 (some adjuvanted with AddaVax) | Doses of 10 μg or 60 μg (mix containing 10 μg of each antigen)  Prime: Day 0  Boost: Day 21  No challenge | PBS | Not performed | NA | Not assessed |
| (Li et al., 2023) | BALB/c mice | Antibodies targeting MPXV antigens | A29L from the MPXV isolate MPXV-M5312_HM12_Rivers (YP_010377135.1) | The antigen A29L was detected by 3 monoclonal antibodies (3A1, 2D1, and 9F8)  These antibodies were further used to evaluate their protective effect against MPXV | Prophylactic assays  Dose: 10 mg/kg  Immunization: Day 0  Challenge: Day 1  Therapeutic assays  Dose: 10 mg/kg  Challenge: Day 0  Immunization: Day 1 | Control antibody | VACV TT or WR: 5×10^6^ TCID_50_ (intranasally) | 21 days | Prophylactic assays: All mice exposed to VACV TT survived. In contrast, infection with VACV WR resulted in complete mortality among the control antibody-treated mice. Meanwhile, in the group treated with the 2D1 antibody, only one mouse challenged with VACV WT succumbed, while all the remaining A29L antibody-treated mice survived.  Therapeutic assays: All mice exposed to VACV TT survived. Almost all vaccinated mice survived in the VACV WR-infected group, except from mice treated with 3A1 and challenged with VACV WR (83.33% survived), and mice treated with 2D1 and challenged with VACV WR (50% survived). Infection with VACV WR resulted in complete mortality among the control antibody-treated mice. |
| (Tang et al., 2023) | BALB/c mice | Recombinant antigens of the MPXV | Not reported | A29L, A35R, B6R, and M1R (adjuvanted with QS-21) | Doses of 15 μg  Prime: Day 0  Boost 1: Day 21  Boost 2: Day 42  Challenge: Day 56 | QS-21 alone or PBS | MPXV (WIBP-MPXV-001): 2.24×10^8^ PFU/mL (intranasally and intraperitoneally) | 5 days | All mice survived the viral challenge, including the QS-21- and PBS-treated controls, which did not show significant weight loss.* |
| (Xia et al., 2023) | C57BL/6 mice | mRNA-LNP vaccine | MPXV isolate MPXV_USA2003_099_Rope_Squirrel (MT903348) | Individual mRNA-LNPs or cocktail of A29L, A35R, B6R, and M1R mRNA-LNPs (named A27, A33, B5, and L1 in the article due to their homologs in VACV) | Doses of 0.5 μg or 5 μg (individual mRNA-LNPs), or 2 μg (mRNA-LNP cocktail with 0.5 μg of each antigen)  Prime: Day 0  Boost: Day 14  Challenge: Day 40 | PBS | VACV TT: 5×10^6^ TCID_50_ (intranasally) | 30 days | All mice vaccinated with the mRNA-LNP cocktail survived, while only 40% of the PBS-treated controls survived.  With the doses of 0.5 μg individual mRNA-LNPs, survival rates were 60% (A27), 80% (A33), 87.5% (B5), and 80% (L1).  At the higher dose of 5 μg, nearly all four mRNA-LNPs provided full protection against mortality, with the exception of A33, which had a survival rate of 80%. |
| (Yang et al., 2023b) | BALB/c mice | Recombinant antigens of the MPXV | MPXV isolate MPXV_USA_2022_MA001 (ON563414) | AMBA: mixture of A29, A35R, B6R, and M1R | Doses of 40 μg (mix containing 10 μg of each antigen)  Prime: Day 0  Boost: Day 14  No challenge | Alum adjuvant or CpG adjuvant | Not performed | NA | Not assessed |
| (Hou et al., 2023) | BALB/c mice | mRNA-LNP vaccine | Clade I MPXV, strain Zaire 79 (accession no. not reported) | VGPox 1 and VGPox 2: A35R full or short extracellular domain combined with a signal peptide and the complete M1R sequence (SP-A35R IECD-M1R and SP-A35R sECD-M1R, respectively)  VGPox 3: A35R and M1R mRNA-LNPs | Data from the single-dose experiment  Dose of 10 μg  Vaccination: Day 0  Challenge: Day 8 | DPBS | VACV WR: 1×10^6^ PFU (intranasally) | 18 days | All mice survived the viral challenge, including the DPBS-treated controls, which showed weight recovery (weight loss did not exceed 30%).* |
| (Freyn et al., 2023) | BALB/c mice | mRNA-LNP vaccine | MPXV isolate MPXV_USA_2022_MA001 (ON563414) | Individual and multi-antigen vaccines of A29, A35, B6, and M1 | Doses of 0.5 μg or 2 μg (individual mRNA-LNPs), or 2 μg or 8 μg (multi-antigen vaccines)  Prime: Week 0  Boost: Week 3  Challenge: Week 3 after boost (multi-antigen vaccines) or week 5 after boost (individual mRNA-LNPs) | PBS | VACV WR: 1×10^6^ PFU (intranasally) | 14 days: mice immunized with multi-antigen vaccines  17 days: mice immunized with individual mRNAs | All mice vaccinated with the multi-antigen vaccines A29+A35+B6+M1, A35+B6+M1, and A35+M1 (2 μg or 8 μg) survived the viral challenge, while all the PBS-treated controls died.  The survival rates for the individual mRNA-LNPs were 0% (A29), 70% (A35), 70% (B6), and 80% (M1) for the dose of 0.5 μg, and 10% (A29), 90% (A35), 90% (B6), and 90% (M1) for the dose of 2 μg. All the PBS-treated controls died. |
| (Yang et al., 2023a) | BALB/c mice | mRNA-LNP vaccine | MPXV isolate MPXV_USA_2022_MA001 (ON563414) | MPXmix: equal concentrations of A29L, A35R, B6R, and M1R mRNA-LNPs  MPXfus: one single mRNA (called MAAB) encoding the 4 antigens joint by flexible linkers (GGGGS) | Doses of 20 μg  Prime: Day 0  Boost: Day 14  No challenge | Empty LNPs | Not performed | NA | Not assessed |
| (Wang et al., 2024) | BALB/c mice | Recombinant antigens of the MPXV | MPXV isolate MPXV_USA_2022_MA001 (ON563414.3) | A35 antigen bivalently fused with the M1 antigen, forming a multi-antigen called DAM (adjuvanted with alum) | Data from the alum-adjuvanted DAM experiment  Doses of 2 μg, 10 μg, or 30 μg  Prime: Day 0  Boost 1: Day 21  Boost 2: Day 42  Challenge: Day 56 | Alum-adjuvanted PBS | VACV WR: 2×10^5^ PFU (intranasally) | 14 days | All vaccinated mice (2 μg, 10 μg, or 30 μg) survived the viral challenge, while all the alum-adjuvanted PBS-treated controls died. |
| (Zuiani et al., 2024) | CAST/Ei mice | mRNA-LNP vaccine | Sequences from early cases of the 2022 outbreak | BNT166a: A35, B6, H3, and M1  BNT166c: A35, B6, and M1  Combination of A35 and B6 mRNA-LNPs | Doses of 4 μg  Prime: Day 0  Boost: Day 21  Challenge: Day 56 | 0.9% NaCl | Clade I MPXV (Zaire 79/V79-I-005): 1×10^5^ PFU (intranasally) | 14 days | All mice vaccinated with BNT166a or BNT166c survived the viral challenge, while all the saline-treated controls died. The survival rate of the A35+B6 vaccine was 30%. |
| (Zuiani et al., 2024) | BALB/c mice | mRNA-LNP vaccine | Sequences from early cases of the 2022 outbreak | BNT166a: A35, B6, H3, and M1  Individual mRNA-LNPs of A35, B6, H3, and M1 | Doses of 4 μg (BNT166a) or 1 μg (individual mRNA-LNPs)  Prime: Day 0  Boost: Day 21  Challenge: Day 42 | 0.9% NaCl | VACV WR: 5×10^4^ PFU (intranasally) | 14 days | All mice vaccinated with BNT166a, A35, B6, or M1 survived the viral challenge, while all the saline-treated controls and H3-treated mice died. |
| (Zuiani et al., 2024) | Cynomolgus macaques | mRNA-LNP vaccine | Sequences from early cases of the 2022 outbreak | BNT166a: A35, B6, H3, and M1 | Doses of 30 μg  Prime: Day 0  Boost: Day 28  Challenge: Day 60 | 0.9% NaCl | Clade I MPXV (Zaire 79/V79-I-005): 5×10^7^ PFU (intratracheally) | 28 days | All macaques vaccinated with BNT166a survived the viral challenge, while only 1 of 6 macaques survived in the saline-treated controls. |
| (Chi et al., 2024) | BALB/c mice | mRNA-LNP encoding antibodies targeting VACV or MPXV antigens | Not reported | mRNA-mab301-LNP: targeting the VACV A27 antigen  mRNA-mab22-LNP: targeting the VACV A33 antigen  mRNA-mab283-LNP: targeting the VACV B5 antigen  mRNA-mab26-LNP: targeting the MPXV M1 antigen  Mix2a: mRNA-mab22-LNP and mRNA-mab26-LNP  Mix2b: The article does not clearly define whether it is a mixture of mRNA-mab26-LNP and mRNA-mab283-LNP or mRNA-mab22-LNP and mRNA-mab301-LNP and | Dose of 1 mg/kg (individual mRNA-LNPs) or 2 mg/kg (cocktails with 1 mg/kg of each mRNA-LNP)  Immunization: Day 0  Challenge: Day 1 | Empty LNPs | VACV WR: 7.5×10^4^ PFU (intranasally) | 7 days | All mice vaccinated with mRNA-mab22-LNP, mRNA-mab26-LNP, mRNA-mab283-LNP, Mix2a, or Mix2b survived, while all the empty LNP-treated controls died. |
| (Su et al., 2024) | BALB/c mice and Sprague Dawley (SD) rats | mRNA-LNP vaccine | Not reported | ALAB-LNP: VACV A27, A33, B5, and L1 antigens in tandem joint by a modified P2A sequence  4Sin-LNP (mixture of individual mRNA-LNPs of VACV A27, A33, B5, and L1) | ALAB-LNP doses for mice: 5 μg, 10 μg, or 20 μg  4Sin-LNP doses for mice: 5 μg, 10 μg, or 20 μg  ALAB-LNP doses for rats: 30 μg, 60 μg, or 100 μg  Prime: Day 0  Boost: Week 4  No challenge | Empty LNPs | Not performed | NA | Not assessed |
| (Zhao et al., 2024) | BALB/c mice | Antibodies targeting MPXV antigens | B6 from the MPXV isolate MPXV_USA_2022_MA001 (URK20605.1) | Antibodies hMB621 and hMB668, which target the B6 antigen | Doses of 10 mg/kg  Prime: Hour 0  Challenge: Hour 4  Boost: Hour 8 | Monoclonal antibody against SARS-CoV-2 or PBS | VACV WR: 5×10^4^ PFU (intranasally) | 6 days | All mice immunized with hMB621 or hMB668 survived de viral challenge.  Although the mice in the SARS-CoV-2 monoclonal antibody-treated and PBS-treated controls did not die, weight curves indicated that there was no recovery of weight loss on day 6, when all mice were euthanized.* |
| (Zhou et al., 2024) | BALB/c mice | circRNA-LNP vaccine | Not reported | cirA29L: encoding A29L  cirA35R: encoding A35R  cirB6R: encoding B6R  cirM1R: encoding M1R  ciRMix4: mixture of the 4 circRNAs | Doses of 5 μg (individual circRNA-LNPs) or 20 μg (mixture with 5 μg of each circRNA-LNP)  Prime: Day 0  Boost: Day 14  Challenge: Day 28 | Empty LNPs | VACV TT: 1×10^6^ PFU (intranasally) | 21 days | All mice immunized with cirA35R, cirB6R, cirM1R, or ciRMix4 survived the viral challenge, while all the empty LNP-treated controls and cirA29L-treated mice died. |
| (Ye et al., 2024b) | BALB/c mice | mRNA-LNP vaccine | Clade I MPXV, Zaire 79/ V79-I-005 (HQ857562.1) | LBA: A29L and B6R joint by the P2A sequence  LAM: A35R and M1R joint by the P2A sequence  LBAAM: A29L, A35R, B6R, and M1R joint by the P2A sequence  LBA&LAM: a mixture of LBA and LAM mRNAs | Doses of 20 μg (10 μg of each mRNA-LNP for LBA&LAM)  Prime: Day 0  Boost: Day 14  Challenge: Day 30 | Empty LNPs | VACV TT: 1×10^6^ PFU (intranasally) | 21 days | All mice vaccinated with LBA, LAM, LBAAM, or LBA&LAM survived the viral challenge, while all the empty LNP-treated controls died. |
| (Li et al., 2024b) | BALB/c mice | Recombinant antigen of the MPXV | Not reported | B6R-BC02: B6R adjuvanted with the BCG-CpG-DNA compound adjuvant System 02 (BC02) | Doses of 5 μg  Prime: Week 0  Boost 1: Week 3  Boost 2: Week 18 (only one group of mice)  No challenge | PBS or naked antigen | Not performed | NA | Not assessed |
| (Li et al., 2024a) | BALB/c mice | mRNA-LNP vaccine | MPXV isolate MPXV-M5312_HM12_Rivers (NC_063383.1) | MPXV-1103: A29, A35, B6, and M1 joint by three flexible linkers (G_4_S_1_)_3_  Individual mRNA-LNPs of A29, A35, B6, and M1  Mix-4-LNPs: a mixture of the 4 mRNA-LNPs | Doses of 5 μg (individual mRNA-LNPs), 1 μg, 5 μg or 20 μg (MPXV-1103), or 5 μg (Mix-4-LNPs, 1.25 μg of each mRNA-LNP)  Prime: Day 0  Boost: Day 14  Challenge: Day 56 | Empty LNPs or PBS | VACV TT: 5×10^6^ PFU (intranasally) | 28 days | All mice vaccinated with MPXV-1103 (1 μg, 5 μg or 20 μg), Mix-4-LNPs, or individual mRNA-LNPs survived the viral challenge, while all the empty LNP-treated and PBS-treated mice died. |
| (Ren et al., 2024) | BALB/c mice | Antibodies targeting MPXV antigens | MPXV isolate MPXV_USA_2022_MA001 (ON563414.3) | Antibodies M1H11 and M3B2, targeting M1R, and B7C9, targeting B6R | Dose of 10 mg/kg  Challenge: Hour 0  Immunization: Hour 12 | Control IgG | VACV TT: 2.5×10^5^ TCID_50_ (infection route not reported) | 10 days | All mice immunized with M1H11, M3B2, B7C9, M1H11+M3B2, M1H11+B7C9, or M3B2+B7C9 survived the viral challenge, as well as the control IgG-treated mice. |
| (Ren et al., 2024) | BALB/c mice | Antibodies targeting MPXV antigens | MPXV isolate MPXV_USA_2022_MA001 (ON563414.3) | Bispecific antibody Bis-M1M3 targeting M1R: Fc region of a chimeric M1H11 conjugated with the scFv of M3B2 | Dose of 5 mg/kg  Challenge: Hour 0  Immunization: Hour 24 | Control IgG | MPXV (strain not reported): 5×10^5^ TCID_50_ (infection route not reported) | 14 days | All mice immunized with Bis-M1M3, cM1M11+cM3B2, cM1M11, or cM3B2 survived the viral challenge, while all the IgG-treated controls died. |
| (Mucker et al., 2024) | Cynomolgus macaques | mRNA-LNP vaccine | Clade IIb MPXV (accession no. not reported) | mRNA-1769: encoding equal concentrations of A29L, A35R, B6R, and M1R | Doses of 150 μg  Prime: Day 0  Boost: Day 26  Challenge: Day 56 | PBS | Clade I MPXV (Zaire 79/ V79-I-005): 5×10^7^ PFU (intravenously) | 26 days | All macaques vaccinated with mRNA-1769 survived the viral challenge, while only 1 of 6 macaques from the PBS-treated controls survived. |
| (Kong et al., 2024) | BALB/c mice | mRNA-LNP vaccine | MPXV isolate MPXV_USA_2022_MA001 (ON563414.3) | Single-chain trivalent vaccines AMAB-wt, AMAB-C140S, and AMB-C140S: encoding soluble A35R, B6R, and M1R antigens  Individual mRNAs-LNPs or cocktails of A35R, B6R, and M1R | Doses of 2.5 μg (individual mRNA-LNPs), 5 μg (two-antigen cocktails), 7.5 μg (three-antigen cocktails), or 7.5 μg (single-chain vaccines)  Prime: Day 0  Boost: Day 14  Challenge: Day 29 or day 39 | Empty LNPs | VACV WR: 1.89×10^5^ PFU (for day 29) or 8.1×10^5^ PFU (for day 39), both intranasally | 14 days | Challenge with 1.89×10^5^ PFU: All mice vaccinated (AMAB-wt, AMAB-C140S, or AMB-C140S) survived the viral challenge, while all the empty LNP-treated controls died.  Challenge with 8.1×10^5^ PFU: All mice vaccinated with AMAB-C140S, AMB-C140S, or sA35R+sB6R+sM1R survived the viral challenge, while AMAB-wt conferred partial protection, with 1 mouse succumbing to infection (80% survival rate). In contrast, all empty LNP-treated controls and A35R+B6R+M1R-treated mice did not survive. |
| (Kong et al., 2024) | BALB/c mice | mRNA-LNP vaccine | MPXV isolate MPXV_USA_2022_MA001 (ON563414.3) | Single-chain trivalent vaccines AMAB-wt, AMAB-C140S, and AMB-C140S: encoding soluble A35R, B6R, and M1R antigens  Individual mRNAs or cocktails of A35R, B6R, and M1R | Doses of 2.5 μg (individual mRNA-LNPs), 5 μg (two-antigen cocktails), 7.5 μg (three-antigen cocktails), or 7.5 μg (single-chain vaccines)  Prime: Day 0  Boost: Day 14  Challenge: Day 190 | Empty LNPs | MPXV (WIBP-MPXV-001): 2×10^7^ PFU (intranasally and intraperitoneally) | 4 days | All mice survived the viral challenge, including the empty LNP-treated controls.* |
| (Tian et al., 2024) | BALB/c mice | mRNA-LNP vaccine | Not reported | A29L | Doses of 5 μg  Prime: Day 0  Boost: Day 21  Challenge: Day 35 | PBS | VACV TT: dose not reported (infection route not reported) | 5 days | All mice survived the viral challenge, including the PBS-treated controls, which showed weight recovery.* |
| (Ye et al., 2024a) | Cynomolgus macaques | mRNA-LNP vaccine | Contemporary clade II MPXV | AR-MPXV5: A29L, A35R, B6R, E8L, and M1R  Continuation of Zhang et al. (2023b) | Doses of 200 μg  Prime: Day 0  Boost: Day 28  Challenge: Day 52 | Empty LNPs | MPXV (MPXV-B.1-China-C-Tan-CQ01): 1×10^7^ TCID_50_ (intravenously) | 10 days | All macaques survived the viral challenge, including the empty LNP-treated controls (no deaths were reported). However, macaques in the control group presented several skin lesions. |
| (Yang et al., 2024) | BALB/c mice | Recombinant antigens of the MPXV | MPXV isolate MPXV_USA_2022_MA001 (ON563414.2) | Mix-AE: A29 and E8  Mix-AEM: A29, E8, and M1  Mix-AEMA: A29, A35, E8, and M1  Mix-AEMB: A29, B6, E8, and M1  Mix-AEMAB: A29, A35, B6, E8, and M1 | Doses of 20 μg  Prime: Day 0  Boost 1: Day 14  Boost 2: Day 28  Challenge: Day 71 | SP01 adjuvant | VACV TT: 4×10^5^ PFU (intranasally) | NA | Survival rates were not reported as mice were euthanized 3 days post-challenge for other analyses. |
| (Cotter et al., 2024) | CAST/Ei mice | mRNA-LNP vaccine | Clade IIb MPXV (accession no. not reported) | mRNA-1769: encoding equal concentrations of A29L, A35R, B6R, and M1R  Continuation of Mucker et al. (2024) | Data from the MPXV protection experiment  Doses of 1 µg or 4 µg  Prime: Week 0  Boost: Week 3  Challenge: Week 6 | PBS | MPXV (MPXV-USA-2003-044): 1×10^5^ PFU (intranasally) or 1×10^4^ PFU (intraperitoneally) | 14 days | Intranasally: All mice vaccinated with mRNA-1769 (1 µg or 4 µg) survived the viral challenge, while 3 mice of the 6 PBS-treated controls died.  Intraperitoneally: All mice vaccinated with mRNA-1769 (1 µg or 4 µg) survived the viral challenge, while only 2 mice of the 6 PBS-treated controls survived. |
| (Chen et al., 2025) | Dormice | Recombinant antigens of the MPXV | MPXV Singapore 2019 strain: A29L (QJQ40281.1) and A35R (QJQ40286.1) | Individually cross-linked antigens: A29L-CC and A35R-CC  Non-cross-linked antigens: A29L and A35R  Some antigens were adjuvanted with aluminum hydroxide | Doses of 20 µg  Prime: Week 0  Boost: Week 3  Challenge: Week 6 | PBS | MPXV (hMpxV/China/GZ8H-01/2023, from a patient in Guangzhou, China): 1×10^5.5^ PFU (intranasally) | 15 days | The survival rates for vaccinated dormice were 37.5% (A29L+A35R+alum), 50% (A29L-CC+A35L-CC), and 87.5% (A29L-CC+A35L-CC+alum). The survival rate for the PBS-treated controls was 12.5%, while A29L+A35R showed no significant benefit over the controls. |
| (Bai et al., 2025) | BALB/c mice | Recombinant antigen of the MPXV | MPXV: A35R (the reported accession no. is MT350282.1, but it belongs to SARS-CoV-2) | A35R-Fc: chimeric A35R protein with the Fc fragment of human IgG1 fused to the C-terminal (adjuvanted with CPG1018 and alum) | Doses of 5 μg (commercial A35R) or 15 μg (A35R-Fc)  Prime: Day 0  Boost: Day 14  No challenge | PBS | Not performed | NA | Not assessed |
| (Li et al., 2025) | BALB/c mice | mRNA-LNP vaccine | MPXV isolate MPXV-M5312_HM12_Rivers (NC_063383.1) | MPXV-1103: A29, A35, B6, and M1 joint by three flexible linkers (G_4_S_1_)_3_  Continuation of Li et al. (2024a) | Doses of 1 μg, 5 μg, or 20 μg  Prime: Day 0  Boost: Day 14  Challenge: Day 280 | PBS or empty LNPs | VACV TT: 5×10^6^ PFU (intranasally) | 21 days | All mice vaccinated with MPXV-1103 (1 μg, 5 μg, or 20 μg) survived the viral challenge, while all the PBS-treated and empty LNP-treated controls died. |

*Data inferred from weight loss curves.

**SUPPLEMENTARY TABLE S3 Bioinformatic studies and patents on MPXV vaccine development without experimental validation in animal models identified through the search strategy.**

| Article title | Authors and year | Source title | DOI | Abstract |
| --- | --- | --- | --- | --- |
| Multi-Epitope-Based Vaccine Candidate for Monkeypox: An In Silico Approach | (Abdi et al., 2022) | Vaccines | 10.3390/vaccines10091564 | Currently, there are limited treatment options available for the monkeypox disease. We used a computational strategy to design a specific antigenic vaccine against pathogens. After using various immunoinformatic tools and filters, cytotoxic T-cell lymphocyte (CTL)-, helper T-cell lymphocyte (HTL)-, and interferon gamma (IFN-γ)-inducing epitopes, which comprised the vaccine, in addition to other parameters, such as antigenic and allergic profiles, were assessed to confirm the safety of the vaccine. However, vaccine interaction and stability with Toll-like receptors (TLRs) were assessed by dynamic simulation methods, and it was found that the constructed vaccine was stable. In addition, C-IMMSIM tools were used to determine the immune-response-triggering capabilities of the vaccine. These immunoinformatic findings reveal that constructed vaccine candidates may be capable of triggering an efficient immune response for monkeypox viral infections. However, experimental evaluation is required to verify the safety and immunogenic profile of constructed vaccines. |
| Immunoinformatic-based design of immune-boosting multiepitope subunit vaccines against monkeypox virus and validation through molecular dynamics and immune simulation | (Suleman et al., 2022) | Frontiers in Immunology | 10.3389/fimmu.2022.1042997 | Monkeypox virus is the causative agent of monkeypox disease, belonging to an orthopoxvirus genus, with a disease pattern similar to that of smallpox. The number of monkeypox cases have robustly increased recently in several countries around the world, potentially causing an international threat. Therefore, serious measures are indispensable to be taken to mitigate the spread of the disease and hence, under these circumstances, vaccination is the best choice to neutralize the monkeypox virus. In the current study, we used immunoinformatic approaches to target the L1R, B5R, and A33R proteins of the monkeypox virus to screen for immunogenic cytotoxic T-lymphocyte (CTL), helper T-lymphocyte (HTL), and B-cell epitopes to construct multiepitope subunit vaccines. Various online tools predicted the best epitope from immunogenic targets (L1R, B5R, and A33R) of monkeypox virus. The predicted epitopes were joined together by different linkers and subjected to 3D structure prediction. Molecular dynamics simulation analysis confirmed the proper folding of the modeled proteins. The strong binding of the constructed vaccines with human TLR-2 was verified by the molecular docking and determination of dissociation constant values. The GC content and codon adaptation index (CAI) values confirmed the high expression of the constructed vaccines in the pET-28a (+) expression vector. The immune response simulation data delineated that the injected vaccines robustly activated the immune system, triggering the production of high titers of IgG and IgM antibodies. In conclusion, this study provided a solid base of concept to develop dynamic and effective vaccines that contain several monkeypox virus-derived highly antigenic and nonallergenic peptides to control the current pandemic of monkeypox virus. |
| Designing, characterization, and immune stimulation of a novel multi-epitopic peptide-based potential vaccine candidate against monkeypox virus through screening its whole genome encoded proteins: An immunoinformatics approach | (Bhattacharya et al., 2022b) | Travel Medicine and Infectious Disease | 10.1016/j.tmaid.2022.102481 | Background: The current monkeypox virus (MPXV) spread in the non-epidemic regions raises global concern. Presently, the smallpox vaccine is used against monkeypox with several difficulties. Conversely, no next-generation vaccine is available against MPXV. Here, we proposed a novel multi-epitopic peptide-based in-silico potential vaccine candidate against the monkeypox virus. Methods: The multi-epitopic potential vaccine construct was developed from antigen screening through whole genome-encoded 176 proteins of MPXV. Afterward, ten common B and T cell epitopes (9-mer) having the highest antigenicity and high population coverage were chosen, and a vaccine construct was developed using peptide linkers. The vaccine was characterized through bioinformatics to understand antigenicity, non-allergenicity, physicochemical properties, and binding affinity to immune receptors (TLR4/MD2-complex). Finally, the immune system simulation of the vaccine was performed through immunoinformatics and machine learning approaches. Results: The highest antigenic epitopes were used to design the vaccine. The docked complex of the vaccine and TLR4/MD2 had shown significant free binding energy (−98.37 kcal/mol) with a definite binding affinity. Likewise, the eigenvalue (2.428517e-05) from NMA analysis of this docked complex reflects greater flexibility, adequate molecular motion, and reduced protein deformability, and it can provoke a robust immune response. Conclusions: The designed vaccine has shown the required effectiveness against MPXV without any side effects, a significant milestone against the neglected disease. |
| Novel multi epitope-based vaccine against monkeypox virus: vaccinomic approach | (Shantier et al., 2022) | Scientific Reports | 10.1038/s41598-022-20397-z | While mankind is still dealing with the COVID-19 pandemic, a case of monkeypox virus (MPXV) has been reported to the WHO on May 7, 2022. Monkeypox is a viral zoonotic disease that has been a public health threat, particularly in Africa. However, it has recently expanded to other parts of the world, so it may soon become a global issue. Thus, the current work was planned and then designed a multi-epitope vaccine against MPXV utilizing the cell surface-binding protein as a target in order to develop a novel and safe vaccine that can evoke the desirable immunological response. The proposed MHC-I, MHC-II, and B-cell epitopes were selected to design multi-epitope vaccine constructs linked with suitable linkers in combination with different adjuvants to enhance the immune responses for the vaccine constructs. The proposed vaccine was composed of 275 amino acids and was shown to be antigenic in Vaxijen server (0.5311) and non-allergenic in AllerTop server. The 3D structure of the designed vaccine was predicted, refined and validated by various in silico tools to assess the stability of the vaccine. Moreover, the solubility of the vaccine construct was found greater than the average solubility provided by protein-Sol server which indicating the solubility of the vaccine construct. Additionally, the most promising epitopes bound to MHC I and MHC II alleles were found having good binding affinities with low energies ranging between − 7.0 and − 8.6 kcal/mol. According to the immunological simulation research, the vaccine was found to elicit a particular immune reaction against the monkeypox virus. Finally, the molecular dynamic study shows that the designed vaccine is stable with minimum RMSF against MHC I allele. We conclude from our research that the cell surface-binding protein is one of the primary proteins involved in MPXV pathogenesis. As a result, our study will aid in the development of appropriate therapeutics and prompt the development of future vaccines against MPXV. |
| Immuno-informatics profiling of monkeypox virus cell surface binding protein for designing a next generation multi-valent peptide-based vaccine | (Yousaf et al., 2022) | Frontiers in Immunology | 10.3389/fimmu.2022.1035924 | Monkeypox is a viral etiological agent with hallmarks analogous to those observed in smallpox cases in the past. The ongoing outbreak of Monkeypox viral infection is becoming a global health problem. Multi-valent peptide based next generation vaccines provides us a promising solution to combat these emerging infectious diseases by eliciting cell-mediated and humoral immune response. Considering the success rate of subtractive proteomics pipeline and reverse vaccinology approach, in this study, we have developed a novel, next-generation, multi-valent, in silico peptide based vaccine construct by employing cell surface binding protein. After analyzing physiochemical and biological properties of the selected target, the protein was subjected to B cell derived T cell epitope mapping. Iterative scrutinization lead to the identification of two highly antigenic, virulent, non-allergic, non-toxic, water soluble, and Interferon-gamma inducer epitopes i.e. HYITENYRN and TTSPVRENY. We estimated that the shortlisted epitopes for vaccine construction, roughly correspond to 99.74% of the world’s population. UK, Finland and Sweden had the highest overall population coverage at 100% which is followed by Austria (99.99%), Germany (99.99%), France (99.98%), Poland (99.96), Croatia (99.93), Czech Republic (99.87%), Belgium (99.87), Italy (99.86%), China (97.83%), India (97.35%) and Pakistan (97.13%). The designed vaccine construct comprises of 150 amino acids with a molecular weight of 16.97242 kDa. Molecular docking studies of the modelled MEMPV (Multi-epitope Monkeypox Vaccine) with MHC I (PDB ID: 1I1Y), MHC II (PDB ID: 1KG0), and other immune mediators i.e. toll like receptors TLR3 (PDB ID: 2A0Z), and TLR4 (PDB ID: 4G8A) revealed strong binding affinity with immune receptors. Host immune simulation results predicted that the designed vaccine has strong potency to induce immune responses against target pathogen in the form of cellular and antibody-dependent immunity. Our findings suggest that the hypothesized vaccine candidate can be utilized as a potential therapeutic against Monkeypox however experimental study is required to validate the results and safe immunogenicity. |
| Multi-Epitope Vaccine Design against Monkeypox Virus via Reverse Vaccinology Method Exploiting Immunoinformatic and Bioinformatic Approaches | (Bhattacharya et al., 2022a) | Vaccines | 10.3390/vaccines10122010 | (1) Background: The monkeypox virus is a zoonotic orthopox DNA virus that is closely linked to the virus. In light of the growing concern about this virus, the current research set out to use bioinformatics and immunoinformatics to develop a potential vaccine against the virus. (2) Methods: A multiepitope vaccine was constructed from the B-cell and T-cell epitopes of the MPXVgp181 strain using adjuvant and different linkers. The constructed vaccine was predicted for antigenicity, allergenicity, toxicity, and population coverage. In silico immune simulation studies were also carried out. Expression analysis and cloning of the constructed vaccine was carried out in the pET-28a(+) vector using snapgene. (3) Results: The constructed vaccine was predicted to be antigenic, non-allergenic, and non-toxic. It was predicted to have excellent global population coverage and produced satisfactory immune response. The in silico expression and cloning studies were successful in E. coli, which makes the vaccine construct suitable for mass production in the pharmaceutical industry. (4) Conclusion: The constructed vaccine is based on the B-cell and T-cell epitopes obtained from the MPXVgp181 strain. This research can be useful in developing a vaccine to combat the monkeypox virus globally after performing in-depth in vitro and in vivo studies. |
| Multi-Epitope Vaccine for Monkeypox Using Pan-Genome and Reverse Vaccinology Approaches | (Swetha et al., 2022) | Viruses | 10.3390/v14112504 | Outbreaks of monkeypox virus infections have imposed major health concerns worldwide, with high morbidity threats to children and immunocompromised adults. Although repurposed drugs and vaccines are being used to curb the disease, the evolving traits of the virus, exhibiting considerable genetic dynamicity, challenge the limits of a targeted treatment. A pan-genome-based reverse vaccinology approach can provide fast and efficient solutions to resolve persistent inconveniences in experimental vaccine design during an outbreak-exigency. The approach encompassed screening of available monkeypox whole genomes (n = 910) to identify viral targets. From 102 screened viral targets, viral proteins L5L, A28, and L5 were finalized based on their location, solubility, and antigenicity. The potential T-cell and B-cell epitopes were extracted from the proteins using immunoinformatics tools and algorithms. Multiple vaccine constructs were designed by combining the epitopes. Based on immunological properties, chemical stability, and structural quality, a novel multi-epitopic vaccine construct, V4, was finalized. Flexible-docking and coarse-dynamics simulation portrayed that the V4 had high binding affinity towards human HLA-proteins (binding energy &lt; -15.0 kcal/mol) with low conformational fluctuations (&lt;1 Å). Thus, the vaccine construct (V4) may act as an efficient vaccine to induce immunity against monkeypox, which encourages experimental validation and similar approaches against emerging viral infections. |
| Contriving multi-epitope vaccine ensemble for monkeypox disease using an immunoinformatics approach | (Aziz et al., 2022) | Frontiers in Immunology | 10.3389/fimmu.2022.1004804 | The current global outbreak of monkeypox (MPX) disease, caused by Monkeypox virus (MPXV), has resulted in 16 thousand infection cases, five deaths, and has been declared a global health emergency of international concern by the World Health Organization. Given current challenges in the safety of existing vaccines, a vaccine to prevent MPX infection and/or onset of symptoms would significantly advance disease management. In this context, a multi-epitope-based vaccine could be a well-suited approach. Herein, we searched a publicly accessible database (Virus Pathogen Database and Analysis Resource) for MPXV immune epitopes from various antigens. We prioritized a group of epitopes (10 CD8+ T cells and four B-cell epitopes) using a computer-aided technique based on desirable immunological and physicochemical properties, sequence conservation criteria, and non-human homology. Three multi-epitope vaccines were constructed (MPXV-1–3) by fusing finalized epitopes with the aid of appropriate linkers and adjuvant (beta-defensin 3, 50S ribosomal protein L7/L12, and Heparin-binding hemagglutinin). Codon optimization and in silico cloning in the pET28a (+) expression vector ensure the optimal expression of each construct in the Escherichia Coli system. Two and three-dimensional structures of the constructed vaccines were predicted and refined. The optimal binding mode of the construct with immune receptors [Toll-like receptors (TLR2, TLR3, and TLR4)] was explored by molecular docking, which revealed high docking energies of MPXV-1–TLR3 (–99.09 kcal/mol), MPXV-2–TLR3 (–98.68 kcal/mol), and MPXV-3–TLR2 (–85.22 kcal/mol). Conformational stability and energetically favourable binding of the vaccine-TLR2/3 complexes were assessed by performing molecular dynamics simulations and free energy calculations (Molecular Mechanics/Generalized Born Surface Area method). In silico immune simulation suggested that innate, adaptive, and humoral responses will be elicited upon administration of such potent multi-epitope vaccine constructs. The vaccine constructs are antigenic, non-allergen, non-toxic, soluble, topographically exposed, and possess favourable physicochemical characteristics. These results may help experimental vaccinologists design a potent MPX vaccine. |
| Multi-epitope chimeric vaccine design against emerging Monkeypox virus via reverse vaccinology techniques- a bioinformatics and immunoinformatics approach | (Aiman et al., 2022) | Frontiers in Immunology | 10.3389/fimmu.2022.985450 | The emerging monkeypox virus (MPXV) is a zoonotic orthopoxvirus that causes infections in humans similar to smallpox. Since May 2022, cases of monkeypox (MPX) have been increasingly reported by the World Health Organization (WHO) worldwide. Currently, there are no clinically validated treatments for MPX infections. In this study, an immunoinformatics approach was used to identify potential vaccine targets against MPXV. A total of 190 MPXV-2022 proteins were retrieved from the ViPR database and subjected to various analyses including antigenicity, allergenicity, toxicity, solubility, IFN-γ, and virulence. Three outer membrane and extracellular proteins were selected based on their respective parameters to predict B-cell and T-cell epitopes. The epitopes are conserved among different strains of MPXV and the population coverage is 100% worldwide, which will provide broader protection against various strains of the virus globally. Nine overlapping MHC-I, MHC-II, and B-cell epitopes were selected to design multi-epitope vaccine constructs linked with suitable linkers in combination with different adjuvants to enhance the immune responses of the vaccine constructs. Molecular modeling and structural validation ensured high-quality 3D structures of vaccine constructs. Based on various immunological and physiochemical properties and docking scores, MPXV-V2 was selected for further investigation. In silico cloning revealed a high level of gene expression for the MPXV-V2 vaccine within the bacterial expression system. Immune and MD simulations confirmed the molecular stability of the MPXV-V2 construct, with high immune responses within the host cell. These results may aid in the development of experimental vaccines against MPXV with increased potency and improved safety. |
| Designing a new Vaccine Based on Multiple Epitopes Against Monkeypox Virus with the help of new Methods Based on Immunoinformatics Software | (Pirmoradi, 2022) | Iranian Journal of Pharmaceutical Sciences | 10.22037/ijps.v18.42143 | Monkeypox virus is a zoonotic virus belonging to the family of Poxviridae and the genus Orthopoxvirus, which is the cause of a viral disease between humans and animals, the characteristics of which are comparable to other cases of smallpox with some differences. Recently some patients caused by the monkeypox virus (MPXV) were reported to the WHO. In recent years, the widespread and rapid spread of some epidemics highlighted the need for the rapid development of effective vaccines in scientific communities. Although conventional therapies have played an essential role in the treatment of many diseases, emerging diseases require new methods of treatment with fewer complications. It is therefore important to develop an effective vaccine for infections caused by the Monkeypox virus to prevent mortality and the safety of the community. In this research, we have used bioinformatics to design a vaccine against the F13 envelope protein Monkeypox virus. A total epitope confined to B cells and MHC I and II alleles were structurally constructed in F13 envelope protein to stimulate immunity and antibody recognition which was used to construct a chimeric peptide vaccine. The vaccine was predicted as a stable, antigenic, and non-allergenic combination. analysis of the TRL4/vaccine docking complex and simulation indicate a sufficiently stable binding with receptor activation. The immune response simulation following hypothetical immunization indicates the potential for stimulation and production of active and memory B cells, as well as the potential for cell production of CD8 + T, CD4 + T, and the development of effective immunological responses induced by Th2 and Th1. Analysis of the in silico processes have shown that the structure of the vaccine produces high antigenicity and good cellular immunity in the host body and stimulates various immune receptors such as TLR4, MHC I and MHC II. Vaccine function was also associated with increased IgM and IgG and a set of Th1 and Th2 cytokines. But final confirmation of the effectiveness of the designed vaccine requires clinical processes. |
| Defining antigen targets to dissect vaccinia virus and monkeypox virus-specific T cell responses in humans | (Grifoni et al., 2022) | Cell Host and Microbe | 10.1016/j.chom.2022.11.003 | The monkeypox virus (MPXV) outbreak confirmed in May 2022 in non-endemic countries is raising concern about the pandemic potential of novel orthopoxviruses. Little is known regarding MPXV immunity in the context of MPXV infection or vaccination with vaccinia-based vaccines (VACV). As with vaccinia, T cells are likely to provide an important contribution to overall immunity to MPXV. Here, we leveraged the epitope information available in the Immune Epitope Database (IEDB) on VACV to predict potential MPXV targets recognized by CD4+ and CD8+ T cell responses. We found a high degree of conservation between VACV epitopes and MPXV and defined T cell immunodominant targets. These analyses enabled the design of peptide pools able to experimentally detect VACV-specific T cell responses and MPXV cross-reactive T cells in a cohort of vaccinated individuals. Our findings will facilitate the monitoring of cellular immunity following MPXV infection and vaccination. |
| A Vaccine Strategy Based on the Identification of an Annular Ganglioside Binding Motif in Monkeypox Virus Protein E8L | (Fantini et al., 2022) | Viruses | 10.3390/v14112531 | The recent outbreak of Monkeypox virus requires the development of a vaccine specifically directed against this virus as quickly as possible. We propose here a new strategy based on a two-step analysis combining (i) the search for binding domains of viral proteins to gangliosides present in lipid rafts of host cells, and (ii) B epitope predictions. Based on previous studies of HIV and SARS-CoV-2 proteins, we show that the Monkeypox virus cell surface-binding protein E8L possesses a ganglioside-binding motif consisting of several subsites forming a ring structure. The binding of the E8L protein to a cluster of gangliosides GM1 mimicking a lipid raft domain is driven by both shape and electrostatic surface potential complementarities. An induced-fit mechanism unmasks selected amino acid side chains of the motif without significantly affecting the secondary structure of the protein. The ganglioside-binding motif overlaps three potential linear B epitopes that are well exposed on the unbound E8L surface that faces the host cell membrane. This situation is ideal for generating neutralizing antibodies. We thus suggest using these three sequences derived from the E8L protein as immunogens in a vaccine formulation (recombinant protein, synthetic peptides or genetically based) specific for Monkeypox virus. This lipid raft/ganglioside-based strategy could be used for developing therapeutic and vaccine responses to future virus outbreaks, in parallel to existing solutions. |
| Immunoinformatics-Aided Design of a Peptide Based Multiepitope Vaccine Targeting Glycoproteins and Membrane Proteins against Monkeypox Virus | (Akhtar et al., 2022) | Viruses | 10.3390/v14112374 | Monkeypox is a self-limiting zoonotic viral disease and causes smallpox-like symptoms. The disease has a case fatality ratio of 3–6% and, recently, a multi-country outbreak of the disease has occurred. The currently available vaccines that have provided immunization against monkeypox are classified as live attenuated vaccinia virus-based vaccines, which pose challenges of safety and efficacy in chronic infections. In this study, we have used an immunoinformatics-aided design of a multi-epitope vaccine (MEV) candidate by targeting monkeypox virus (MPXV) glycoproteins and membrane proteins. From these proteins, seven epitopes (two T-helper cell epitopes, four T-cytotoxic cell epitopes and one linear B cell epitopes) were finally selected and predicted as antigenic, non-allergic, interferon-γ activating and non-toxic. These epitopes were linked to adjuvants to design a non-allergic and antigenic candidate MPXV-MEV. Further, molecular docking and molecular dynamics simulations predicted stable interactions between predicted MEV and human receptor TLR5. Finally, the immune-simulation analysis showed that the candidate MPXV-MEV could elicit a human immune response. The results obtained from these in silico experiments are promising but require further validation through additional in vivo experiments. |
| Análisis in silico de un candidato a vacuna multi-epítopo contra viruela del mono usando vaculonogía reversa | (Montenegro Oyola et al., 2022) | Magazine of the Colombian Association of Biological Sciences / Revista de la Asociación Colombiana de Ciencias Biológicas (ACCB) | 10.47499/revistaaccb.v1i34.265 | Introduction. Monkey pox is a zoonotic infection with an increased global transmission rate during 2022, denoted epidemiological trouble in public health. Currently, the disease has no specific treatments available; thus, a preventive approach can be achieved through immunization. Objective. was to design in silico a vaccine applying advanced computational techniques using a multi-epitope construct of the Monkeypox virus. Materials and methods. Antigens were selected based on reports about proteins that cause the activation of cytotoxic T and B lymphocytes. The immunoinformatics assays were antigenicity, allergenicity, toxicity, MHC binding affinity, and IFN-Î³ stimulation. Results and discussion. Eight epitopes of the M1R, DNA polymerase, B6R, and A35R proteins of the M. virus showed a significant response for immune cells. Eleven epitopes with antigenicity >0.3, non-allergenic and non-toxic were chosen, of which 4 presented high affinity to T lymphocytes, 4 generated high activation of B lymphocytes, and 3 were associated with IFN-Î³ activation results. The in silico construction of the 509-amino acid vaccine candidate with high topological similarity registered mainly a negative charge, in addition to being soluble with an aliphatic index >80%, stable and particular with MHC activation and high molecular affinity with TLR-3, and also presented multi-antigenicity, similar to vaccines generated by this methodology with M. tuberculosis and Influenza. One-dose injection simulation of the molecular construct showed activation of T helper plasma cells for about 15 to 25 days and high expression of IFN-Î³ and IL-2 for eight days. Conclusion. These results indicate an excellent immunization process that could be potentiated with multi-dosing. |
| Delineating multi-epitopes vaccine designing from membrane protein CL5 against all monkeypox strains: a pangenome reverse vaccinology approach | (Alsaiari et al., 2023) | Journal of Biomolecular Structure and Dynamics | 10.1080/07391102.2023.2248301 | The recently identified monkeypox virus (MPXV or mpox) is a zoonotic orthopox virus that infects humans and causes diseases with traits like smallpox. The world health organization (WHO) estimates that 3-6% of MPXV cases result in death. As it might impact everyone globally, like COVID, and become the next pandemic, the cure for this disease is important for global public health. The high incidence and disease ratio of MPXV necessitates immediate efforts to design a unique vaccine candidate capable of addressing MPXV diseases. Here, we used a computational pan-genome-based vaccine design strategy for all currently reported 19 MPXV strains acquired from different regions of the world. Thus, this study's objective was to develop a new and safe vaccine candidate against MPXV by targeting the membrane CL5 protein; identified after the pangenome analysis. Proteomics and reverse vaccinology have covered up all of the MPXV epitopes that would usually stimulate robust host immune responses. Following this, only two mapped (MHC-I, MHC-II, and B-cell) epitopes were observed to be extremely effective that can be used in the construction of CL5 protein vaccine candidates. The suggested vaccine (V5) candidate from eight vaccine models was shown to be antigenic, non-allergenic, and stable (with 213 amino acids). The vaccine's candidate efficacy was evaluated by using many in silico methods to predict, improve, and validate its 3D structure. Molecular docking and molecular dynamics simulations further reveal that the proposed vaccine candidate ensemble has a high interaction energy with the HLAs and TRL2/4 immunological receptors under study. Later, the vaccine sequence was used to generate an expression vector for the E. coli K12 strain. Further study uncovers that V5 was highly immunogenic because it produced robust primary, secondary, and tertiary immune responses. Eventually, the use of computer-aided vaccine designing may significantly reduce costs and speed up the process of developing vaccines. Although, the results of this research are promising, however, more research (experimental; in vivo, and in vitro studies) is needed to verify the biological efficacy of the proposed vaccine against MPXV. |
| Immunoinformatics-based multi-epitope vaccine design for the re-emerging monkeypox virus | (Farzan et al., 2023) | International Immunopharmacology | 10.1016/j.intimp.2023.110725 | Background: On May 7, 2022, WHO reported a new monkeypox case. By May 2023 over 80,000 cases had been reported worldwide outside previously endemic nations. (This primarily affected the men who have sex with men (MSM) community in rich nations). The present research aims to develop a multi-epitope vaccine for the monkeypox virus (MPXV) using structural and cell surface proteins. Methods: The first part of the research involved retrieving protein sequences. The Immune Epitope Database (IEDB) was then used to analyze the B and T lymphocyte epitopes. After analyzing the sensitizing properties, toxicity, antigenicity, and molecular binding, appropriate linkers were utilized to connect selected epitopes to adjuvants, and the structure of the vaccine was formulated. Algorithms from the field of immunoinformatics predicted the secondary and tertiary structures of vaccines. The physical, chemical, and structural properties were refined and validated to achieve maximum stability. Molecular docking and molecular dynamic simulations were subsequently employed to assess the vaccine's efficacy. Afterward, the ability of the vaccine to interact with toll-like receptors 3 and 4 (TLR3 and TLR4) was evaluated. Finally, the optimized sequence was then introduced into the Escherichia coli (E. coli) PET30A + vector. Results: An immunoinformatics evaluation suggested that such a vaccine might be safe revealed that this vaccine is safe, hydrophilic, temperature- and condition-stable, and can stimulate innate immunity by binding to TLR3 and TLR4. Conclusion: Our findings suggest that the first step in MPXV pathogenesis is structural and cell surface epitopes. In this study, the most effective and promising epitopes were selected and designed through precision servers. Furthermore, through the utilization of multi-epitope structures and a combination of two established adjuvants, this research has the potential to be a landmark in developing an antiviral vaccine against MPXV. However, additional in vitro and in vivo tests are required to confirm these results. |
| Design peptide and multi-epitope protein vaccine candidates against monkeypox virus using reverse vaccinology approach: an in-silico study | (Jahantigh et al., 2023) | Journal of Biomolecular Structure and Dynamics | 10.1080/07391102.2023.2201850 | Monkeypox is a zoonotic virus that has recently affected different countries worldwide. On July 23, 2022, the WHO declared the outbreak of monkeypox as a public health emergency of international concern. Surveillance studies conducted in Central Africa in the 1980s and later during outbreaks in the same region showed smallpox vaccines to be clinically somewhat effective against Monkeypox virus. However, there is no specific vaccine against this virus. This research used bioinformatics techniques to establish a novel multi-epitope vaccine candidate against Monkeypox that can induce a strong immune response. Five well-known antigenic proteins (E8L, A30L, A35R, A29L, and B21R) of the virus were picked and assessed as possible immunogenic peptides. Two suitable peptide candidates were selected according to bio-informatics analysis. Based upon in silico evaluation, two multi-epitope vaccine candidates (ALALAR and ALAL) were built with rich-epitope domains consisting of high-ranking T and B-cell epitopes. After predicting and evaluating the 3D structure of the protein candidates, the most efficient 3D models were considered for docking studies with Toll-like receptor 4 (TLR4) and the HLA-A * 11:01, HLA-A*01:01, HLA-A*02:01, HLA-A*03:01, HLA-A*07:02, HLA-A*15:01, HLA-A*30:01 receptors. Subsequently, molecular dynamics (MD) simulation of up to 150 nanoseconds was employed to assess the durability of the interaction of the vaccine candidates with immune receptors. MD studies showed that M5-HLA-A*11:01, ALAL-TLR4, and ALALAR-TLR4 complexes were stable during simulation. Analysis of the in silico outcomes indicates that the M5 peptide and ALAL and ALALAR proteins may be suitable vaccine candidates against the Monkeypox virus. |
| Deciphering the Immunogenicity of Monkeypox Proteins for Designing the Potential mRNA Vaccine | (Shah et al., 2023) | ACS Omega | 10.1021/acsomega.3c07866 | The Monkeypox virus (MPXV), an orthopox virus, is responsible for monkeypox in humans, a zoonotic disease similar to smallpox. This infection first appeared in the 1970s in humans and then in 2003, after which it kept on spreading all around the world. To date, various antivirals have been used to cure this disease, but now, MPXV has developed resistance against these, thus increasing the need for an alternative cure for this deadly disease. In this study, we devised a reverse vaccinology approach against MPXV using a messenger RNA (mRNA) vaccine by pinning down the antigenic proteins of this virus. By using bioinformatic tools, we predicted prospective immunogenic B and T lymphocyte epitopes. Based on cytokine inducibility score, nonallergenicity, nontoxicity, antigenicity, and conservancy, the final epitopes were selected. Our analysis revealed the stable structure of the mRNA vaccine and its efficient expression in host cells. Furthermore, strong interactions were demonstrated with toll-like receptors 2 (TLR2) and 4 (TLR4) according to the molecular dynamic simulation studies. The in silico immune simulation analyses revealed an overall increase in the immune responses following repeated exposure to the designed vaccine. Based on our findings, the vaccine candidate designed in this study has the potential to be tested as a promising novel mRNA therapeutic vaccine against MPXV infection. |
| Translational vaccinomics and structural filtration algorithm to device multiepitope vaccine for catastrophic monkeypox virus | (Singh et al., 2023) | Computers in Biology and Medicine | 10.1016/j.compbiomed.2022.106497 | Recent outbreak of monkeypox disease commenced in April 2022, and on May 7, the first confirmed case was reported. The world health organization then designated monkeypox disease as a public health emergency of international outrage on July 23, after it spread to 70 non-endemic nations in less than 15 days. This catastrophic viral infection encourages the development of antiviral therapeutics due to the lack of specific treatments with negligible adverse effects. This analysis developed a highly immunogenic multiepitope subunit vaccine against the monkeypox virus using an in silico translational vaccinomics technique. Highly antigenic B cell and T cell (HTL and CTL) epitopes were predicted and conjugated with the help of unique linkers. An adjuvant (β-defensin) and a pan-HLA DR sequence were attached at the vaccine construct's N-terminal to invoke a robust immunological response. Additionally, physiochemical, allergic, toxic, and antigenic properties were anticipated. Interactions between the vaccine candidate and the TLR3 demonstrated that the vaccine candidate triggers a robust immunological response. Finally, the stability is confirmed by the molecular dynamics study. In contrast, the modified vaccine candidate's ability to produce a protective immune response were verified by an immune dynamics simulation study conducted via C-ImmSim server. This study validates the generation of B cell, Th cell, and Tc cell populations as well as the production of IFN‐γ. |
| Development of a Multi-Epitope Universal mRNA Vaccine Candidate for Monkeypox, Smallpox, and Vaccinia Viruses: Design and In Silico Analyses | (Rcheulishvili et al., 2023b) | Viruses | 10.3390/v15051120 | Notwithstanding the presence of a smallpox vaccine that is effective against monkeypox (mpox), developing a universal vaccine candidate against monkeypox virus (MPXV) is highly required as the mpox multi-country outbreak has increased global concern. MPXV, along with variola virus (VARV) and vaccinia virus (VACV), belongs to the Orthopoxvirus genus. Due to the genetic similarity of antigens in this study, we have designed a potentially universal mRNA vaccine based on conserved epitopes that are specific to these three viruses. In order to design a potentially universal mRNA vaccine, antigens A29, A30, A35, B6, and M1 were selected. The conserved sequences among the three viral species-MPXV, VACV, and VARV-were detected, and B and T cell epitopes containing the conserved elements were used for the design of the multi-epitope mRNA construct. Immunoinformatics analyses demonstrated the stability of the vaccine construct and optimal binding to MHC molecules. Humoral and cellular immune responses were induced by immune simulation analyses. Eventually, based on in silico analysis, the universal mRNA multi-epitope vaccine candidate designed in this study may have a potential protection against MPXV, VARV, and VACV that will contribute to the advancement of prevention strategies for unpredictable pandemics. |
| Identification of B and T Cell Epitopes to Design an Epitope-Based Peptide Vaccine against the Cell Surface Binding Protein of Monkeypox Virus: An Immunoinformatics Study | (Mazumder et al., 2023) | Journal of Immunology Research | 10.1155/2023/2274415 | Background. Although the monkeypox virus-associated illness was previously confined to Africa, recently, it has started to spread across the globe and become a significant threat to human lives. Hence, this study was designed to identify the B and T cell epitopes and develop an epitope-based peptide vaccine against this virus's cell surface binding protein through an in silico approach to combat monkeypox-associated diseases. Results. The analysis revealed that the cell surface binding protein of the monkeypox virus contains 30 B cell and 19 T cell epitopes within the given parameter. Among the T cell epitopes, epitope "ILFLMSQRY"was found to be one of the most potential peptide vaccine candidates. The docking analysis revealed an excellent binding affinity of this epitope with the human receptor HLA-B∗15:01 with a very low binding energy (-7.5 kcal/mol). Conclusion. The outcome of this research will aid the development of a T cell epitope-based peptide vaccine, and the discovered B and T cell epitopes will facilitate the creation of other epitope and multi-epitope-based vaccines in the future. This research will also serve as a basis for further in vitro and in vivo analysis to develop a vaccine that is effective against the monkeypox virus. |
| Development of multi-epitope vaccines against the monkeypox virus based on envelope proteins using immunoinformatics approaches | (Tan et al., 2023) | Frontiers in Immunology | 10.3389/fimmu.2023.1112816 | Background: Since May 2022, cases of monkeypox, a zoonotic disease caused by the monkeypox virus (MPXV), have been increasingly reported worldwide. There are, however, no proven therapies or vaccines available for monkeypox. In this study, several multi-epitope vaccines were designed against the MPXV using immunoinformatics approaches. Methods: Three target proteins, A35R and B6R, enveloped virion (EV) form-derived antigens, and H3L, expressed on the mature virion (MV) form, were selected for epitope identification. The shortlisted epitopes were fused with appropriate adjuvants and linkers to vaccine candidates. The biophysical andbiochemical features of vaccine candidates were evaluated. The Molecular docking and molecular dynamics(MD) simulation were run to understand the binding mode and binding stability between the vaccines and Toll-like receptors (TLRs) and major histocompatibility complexes (MHCs). The immunogenicity of the designed vaccines was evaluated via immune simulation. Results: Five vaccine constructs (MPXV-1-5) were formed. After the evaluation of various immunological and physicochemical parameters, MPXV-2 and MPXV-5 were selected for further analysis. The results of molecular docking showed that the MPXV-2 and MPXV-5 had a stronger affinity to TLRs (TLR2 and TLR4) and MHC (HLA-A*02:01 and HLA-DRB1*02:01) molecules, and the analyses of molecular dynamics (MD) simulation have further confirmed the strong binding stability of MPXV-2 and MPXV-5 with TLRs and MHC molecules. The results of the immune simulation indicated that both MPXV-2 and MPXV-5 could effectively induce robust protective immune responses in the human body. Conclusion: The MPXV-2 and MPXV-5 have good efficacy against the MPXV in theory, but further studies are required to validate their safety and efficacy. |
| Immunoinformatics design of multivalent epitope vaccine against monkeypox virus and its variants using membrane-bound, enveloped, and extracellular proteins as targets | (Waqas et al., 2023) | Frontiers in Immunology | 10.3389/fimmu.2023.1091941 | Introduction: The current monkeypox (MPX) outbreak, caused by the monkeypox virus (MPXV), has turned into a global concern, with over 59,000 infection cases and 23 deaths worldwide. Objectives: Herein, we aimed to exploit robust immunoinformatics approach, targeting membrane-bound, enveloped, and extracellular proteins of MPXV to formulate a chimeric antigen. Such a strategy could similarly be applied for identifying immunodominant epitopes and designing multi-epitope vaccine ensembles in other pathogens responsible for chronic pathologies that are difficult to intervene against. Methods: A reverse vaccinology pipeline was used to select 11 potential vaccine candidates, which were screened and mapped to predict immunodominant B-cell and T-cell epitopes. The finalized epitopes were merged with the aid of suitable linkers, an adjuvant (Resuscitation-promoting factor), a PADRE sequence (13 aa), and an HIV TAT sequence (11 aa) to formulate a multivalent epitope vaccine. Bioinformatics tools were employed to carry out codon adaptation and computational cloning. The tertiary structure of the chimeric vaccine construct was modeled via I-TASSER, and its interaction with Toll-like receptor 4 (TLR4) was evaluated using molecular docking and molecular dynamics simulation. C-ImmSim server was implemented to examine the immune response against the designed multi-epitope antigen. Results and discussion: The designed chimeric vaccine construct included 21 immunodominant epitopes (six B-cell, eight cytotoxic T lymphocyte, and seven helper T-lymphocyte) and is predicted non-allergen, antigenic, soluble, with suitable physicochemical features, that can promote cross-protection among the MPXV strains. The selected epitopes indicated a wide global population coverage (93.62%). Most finalized epitopes have 70%–100% sequence similarity with the experimentally validated immune epitopes of the vaccinia virus, which can be helpful in the speedy progression of vaccine design. Lastly, molecular docking and molecular dynamics simulation computed stable and energetically favourable interaction between the putative antigen and TLR4. Conclusion: Our results show that the multi-epitope vaccine might elicit cellular and humoral immune responses and could be a potential vaccine candidate against the MPXV infection. Further experimental testing of the proposed vaccine is warranted to validate its safety and efficacy profile. |
| Vaccinomics to design a multi-epitope-based vaccine against monkeypox virus using surface-associated proteins | (Khan et al., 2023b) | Journal of Biomolecular Structure and Dynamics | 10.1080/07391102.2022.2158942 | In 2022, the ongoing multi-country outbreak of monkeypox virus—now occurring outside Africa, too is a global health concern. Monkeypox is a zoonotic virus, which causes disease mainly in animals, and then it is transferred to humans. Recently, in the monkeypox epidemic, a large number of human cases emerged while the global health community worked to tackle the outbreak and save lives. Herein, a multi-epitope-based vaccine is designed against monkeypox virus using two surface-associated proteins: MPXVgp002 accession number > YP_010377003.1 and MPXVgp008 accession number > YP_010377007.1 proteins. These proteins were utilized for B- and T-cell epitopes prediction. The epitopes were further screened, and the screen filtered KCKDNEYRSR, RSCNTTHNR, and RTRRETGAS with the antigenicity scores of 0.5279, 0.5604, and 0.7628, respectively. Overall, the epitopes can induce immunity in 99.74% population of the world. Further, GPGPG linkers were used for joining the epitopes and EAAAK linker was used for adjuvant attachment. It has a three-dimensional structure modelled for retaining the structural stability. Three pairs of amino acid residues that were able to make disulfide bonds were chosen: Gly1-Ser82, Cys7-Tyr10, and Phe51-Ile55. Molecular docking of vaccine was done with toll-like receptors, viz., 2, 3, 4, and 8 immune cell receptors. The docking results revealed that the vaccine as potential molecule due to its better binding affinity with toll-like receptors 2, 3, 4 and 8. Top complex in docking in with each receptor was selected based on lowest energy scores— −888.7 kcal/mol (TLR-2), −976.3 kcal/mol (TLR-3), −801.9 kcal/mol (TLR-4), and −955.4 kcal/mol (TLR-4)—were subjected to simulation. The docked complexes were evaluated in 500 ns of MD simulation. Throughout the simulation time, no significant deviation occurred. This confirmed that the vaccine as potential vaccine candidate to interact with immune cell receptors. This interaction is important for the immune system activation. In conclusion, the proposed vaccine construct against monkeypox could induce an effective immune response and speed up the vaccine development process. However, the study is completely based on the computational approach, hence, the experimental validation is required. |
| Developing a multiepitope vaccine for the prevention of SARS-CoV-2 and monkeypox virus co-infection: A reverse vaccinology analysis | (Jiang et al., 2023) | International Immunopharmacology | 10.1016/j.intimp.2023.109728 | Background: Severe acute respiratory syndrome coronavirus 2 (SARS-CoV-2) and monkeypox virus (MPXV) severely threaten human health; however, currently, no vaccine can prevent a co-infection with both viruses. Methods: Five antigens were selected to predict dominant T and B cell epitopes screened for immunogenicity, antigenicity, toxicity, and sensitization. After screening, all antigens joined in the construction of a novel multiepitope vaccine. The physicochemical and immunological characteristics, and secondary and tertiary structures of the vaccine were predicted and analyzed using bio- and immunoinformatics. Finally, codon optimization and cloning in-silico were performed. Results: A new multiepitope vaccine, named S7M8, was constructed based on four helper T lymphocyte (HTL) epitopes, six cytotoxic T lymphocyte (CTL) epitopes, five B cell epitopes, as well as Toll-like receptor (TLR) agonists. The antigenicity and immunogenicity scores of the S7M8 vaccine were 0.907374 and 0.6552, respectively. The S7M8 vaccine was comprised of 26.96% α-helices, the optimized Z-value of the tertiary structure was −5.92, and the favored area after majorization in the Ramachandran plot was 84.54%. Molecular docking showed that the S7M8 vaccine could tightly bind to TLR2 (−1100.6 kcal/mol) and TLR4 (−950.3 kcal/mol). In addition, the immune stimulation prediction indicated that the S7M8 vaccine could activate T and B lymphocytes to produce high levels of Th1 cytokines and antibodies. Conclusion: S7M8 is a promising biomarker with good antigenicity, immunogenicity, non-toxicity, and non-sensitization. The S7M8 vaccine can trigger significantly high levels of Th1 cytokines and antibodies and may be a potentially powerful tool in preventing SARS-CoV-2 and MPXV. |
| In silico design and immunoinformatics analysis of a universal multi-epitope vaccine against monkeypox virus | (Sanami et al., 2023) | PLoS ONE | 10.1371/journal.pone.0286224 | Monkeypox virus (MPXV) outbreaks have been reported in various countries worldwide; however, there is no specific vaccine against MPXV. In this study, therefore, we employed computational approaches to design a multi-epitope vaccine against MPXV. Initially, cytotoxic T lymphocyte (CTL), helper T lymphocyte (HTL), linear B lymphocytes (LBL) epitopes were predicted from the cell surface-binding protein and envelope protein A28 homolog, both of which play essential roles in MPXV pathogenesis. All of the predicted epitopes were evaluated using key parameters. A total of 7 CTL, 4 HTL, and 5 LBL epitopes were chosen and combined with appropriate linkers and adjuvant to construct a multi-epitope vaccine. The CTL and HTL epitopes of the vaccine construct cover 95.57% of the worldwide population. The designed vaccine construct was found to be highly antigenic, non-allergenic, soluble, and to have acceptable physicochemical properties. The 3D structure of the vaccine and its potential interaction with Toll-Like receptor-4 (TLR4) were predicted. Molecular dynamics (MD) simulation confirmed the vaccine's high stability in complex with TLR4. Finally, codon adaptation and in silico cloning confirmed the high expression rate of the vaccine constructs in strain K12 of Escherichia coli (E. coli). These findings are very encouraging; however, in vitro and animal studies are needed to ensure the potency and safety of this vaccine candidate. |
| Proteomics-based vaccine targets annotation and design of subunit and mRNA-based vaccines for Monkeypox virus (MPXV) against the recent outbreak | (Jin et al., 2023) | Computers in Biology and Medicine | 10.1016/j.compbiomed.2023.106893 | Monkeypox Virus (MPXV) is a growing public health threat with increasing cases and fatalities globally. To date, no specific vaccine or small molecule therapeutic choices are available for the treatment of MPXV disease. In this work, we employed proteomics and structural vaccinology approaches to design mRNA and multi-epitopes-based vaccines (MVC) against MPXV. We first identified ten proteins from the whole proteome of MPXV as potential vaccine targets. We then employed structural vaccinology approaches to map potential epitopes of these proteins for B cell, cytotoxic T lymphocytes (CTL), and Helper T lymphocytes (HTL). Finally, 9 CTL, 6 B cell, and 5 HTL epitopes were joined together through suitable linkers to construct MVC (multi-epitope vaccine) and mRNA-based vaccines. Molecular docking, binding free energy calculation, and in silico cloning revealed robust interaction of the designed MVC with toll-like receptor 2 (TLR2) and efficient expression in E. Coli K12 strain. The immune simulation results revealed that the antigen titer after the injection reached to the maximum level on the 5th day and an abrupt decline in the antigen titer was observed upon the production of IgM, IgG and IgM + IgG, dendritic cells, IFN-gamma, and IL (interleukins), which suggested the potential of our designed vaccine candidate for inducing an immune response against MPXV. |
| Computational Vaccine Design for Poxviridae Family Viruses | (Khan et al., 2023a) | Methods in Molecular Biology | 10.1007/978-1-0716-3239-0_31 | The computational approach to designing vaccines has several useful characteristics over traditional vaccine development, such as being highly specific, less time-consuming and less expensive. Thus, this chapter describes an immunoinformatics workflow to design a vaccine against a member of the Poxviridae family known as Monkeypox virus. The immunoinformatics approach uses several online servers to select highly antigenic and non-allergenic CTL, HTL, and B cell epitopes. Then, it links the predicted epitopes through linkers and submit them for 3D structure modeling. Afterward, the modeled vaccine is docked with TLRs to check the induction of the immune system. Finally, immune simulations are performed to check the level of several immune factors like IgG, IgM, cytokines and interleukins, among others, upon the injection of the constructed vaccine. This approach can be used to successfully design novel and effective vaccine candidates against emerging species from the Poxviridae family. |
| Computer-Aided Multi-Epitope Based Vaccine Design Against Monkeypox Virus Surface Protein A30L: An Immunoinformatics Approach | (Ramprasadh et al., 2023) | Protein Journal | 10.1007/s10930-023-10150-4 | Monkeypox, a viral zoonotic disease resembling smallpox, has emerged as a significant national epidemic primarily in Africa. Nevertheless, the recent global dissemination of this pathogen has engendered apprehension regarding its capacity to metamorphose into a sweeping pandemic. To effectively combat this menace, a multi-epitope vaccine has been meticulously engineered with the specific aim of targeting the cell envelope protein of Monkeypox virus (MPXV), thereby stimulating a potent immunological response while mitigating untoward effects. This new vaccine uses T-cell and B-cell epitopes from a highly antigenic, non-allergenic, non-toxic, conserved, and non-homologous A30L protein to provide protection against the virus. In order to ascertain the vaccine design with the utmost efficacy, protein–protein docking methodologies were employed to anticipate the intricate interactions with Toll-like receptors (TLR) 2, 3, 4, 6, and 8. This meticulous approach led the researchers to discern an optimal vaccine architecture, bolstered by affirmative prognostications derived from both molecular dynamics (MD) simulations and immune simulations. The current research findings indicate that the peptides ATHAAFEYSK, FFIVVATAAV, and MNSLSIFFV exhibited antigenic properties and were determined to be non-allergenic and non-toxic. Through the utilization of codon optimization and in-silico cloning techniques, our investigation revealed that the prospective vaccine exhibited a remarkable expression level within Escherichia coli. Moreover, upon conducting immune simulations, we observed the induction of a robust immune response characterized by elevated levels of both B-cell and T-cell mediated immunity. Moreover, as the initial prediction with in-silico techniques has yielded promising results these epitope-based vaccines can be recommended to in vitro and in silico studies to validate their immunogenic properties. |
| Immunoinformatics and reverse vaccinology approach in designing a novel highly immunogenic multivalent peptide-based vaccine against the human monkeypox virus | (Choudhury et al., 2023) | Frontiers in Molecular Biosciences | 10.3389/fmolb.2023.1295817 | Background: Monkeypox is a highly infectious zoonotic disease, often resulting in complications ranging from respiratory illnesses to vision loss. The escalating global incidence of its cases demands prompt attention, as the absence of a proven post-exposure treatment underscores the criticality of developing an effective vaccine. Methods: Interactions of the viral proteins with TLR2 and TLR4 were investigated to assess their immunogenic potentials. Highly immunogenic proteins were selected and subjected to epitope mapping for identifying B-cell and MHC class I and II epitopes. Epitopes with high antigenicity were chosen, considering global population coverage. A multi-target, multi-epitope vaccine peptide was designed, incorporating a beta-defensin 2 adjuvant, B-cell epitopes, and MHC class I and II epitopes. Results: The coordinate structure of the engineered vaccine was modeled and validated. In addition, its physicochemical properties, antigenicity, allergenicity, and virulence traits were evaluated. Molecular docking studies indicated strong interactions between the vaccine peptide and the TLR2 receptor. Furthermore, molecular dynamics simulations and immune simulation studies reflected its potent cytosolic stability and robust immune response dynamics induced by the vaccine. Conclusion: This study explored an innovative structure-guided approach in the use of immunoinformatics and reverse vaccinology in pursuit of a novel multi-epitope vaccine against the highly immunogenic monkeypox viral proteins. The simulation studies indicated the engineered vaccine candidate to be promising in providing prophylaxis to the monkeypox virus; nevertheless, further in vitro and in vivo investigations are required to prove its efficacy. |
| An immunoinformatic approach towards development of a potent and effective multi-epitope vaccine against monkeypox virus (MPXV) | (Mishra et al., 2023) | Journal of Biomolecular Structure and Dynamics | 10.1080/07391102.2022.2163426 | Monkeypox is a viral zoonotic disease, often transmitted to humans from animals. While the whole world is haggling with the COVID-19 pandemic, the emergence of the monkeypox virus (MPXV) arose as a new challenge to mankind. Till date, numerous cases related to the MPXV have been reported in several countries across the globe, but, its momentary distribution in the current time has left everyone in fright with increasing mortality and limited clinically approved treatments. Therefore, it is of immense importance to develop a potent and highly effective vaccine capable of inducing desired immunogenic responses against the highly contagious MPXV. Herein, using various immunoinformatic and computational biology tools, we made an attempt to develop a multi-epitope vaccine construct against the MPXV which is antigenic, non-allergen and non-toxic in nature and capable of exhibiting immunogenic behavior. The sequence of vaccine construct was designed using the proposed 4 MHC-I, 3 MHC-II and 4 B-cell epitopes linked with suitable adjuvant and linkers. The modeled structure of the vaccine construct was used to assess its interaction with the Toll-like Receptor 4 (TLR4) using ClusPro and HADDOCK. All-atoms molecular dynamics simulation of the MPXV vaccine construct-TLR4 complex followed by a high level of gene expression of the construct within the bacterial system affirmed its stability along with induction of immunogenic response within the host cell. Altogether, our immunoinformatic approach aid in the development of a stable chimeric vaccine construct against MPXV and needs further experimental validation for its immunological relevance and usefulness as a vaccine candidate. |
| Design, evaluation, and immune simulation of potentially universal multi-epitope mpox vaccine candidate: focus on DNA vaccine | (Rcheulishvili et al., 2023a) | Frontiers in Microbiology | 10.3389/fmicb.2023.1203355 | Monkeypox (mpox) is a zoonotic infectious disease caused by the mpox virus. Mpox symptoms are similar to smallpox with less severity and lower mortality. As yet mpox virus is not characterized by as high transmissibility as some severe acute respiratory syndrome 2 (SARS-CoV-2) variants, still, it is spreading, especially among men who have sex with men (MSM). Thus, taking preventive measures, such as vaccination, is highly recommended. While the smallpox vaccine has demonstrated considerable efficacy against the mpox virus due to the antigenic similarities, the development of a universal anti-mpox vaccine remains a necessary pursuit. Recently, nucleic acid vaccines have garnered special attention owing to their numerous advantages compared to traditional vaccines. Importantly, DNA vaccines have certain advantages over mRNA vaccines. In this study, a potentially universal DNA vaccine candidate against mpox based on conserved epitopes was designed and its efficacy was evaluated via an immunoinformatics approach. The vaccine candidate demonstrated potent humoral and cellular immune responses in silico, indicating the potential efficacy in vivo and the need for further research. |
| Exploring the whole proteome of monkeypox virus to design B cell epitope-based oral vaccines using immunoinformatics approaches | (Pritam, 2023) | International Journal of Biological Macromolecules | 10.1016/j.ijbiomac.2023.126498 | In the last few months 85,536 cases and 91 deaths were reported for monkeypox disease from 110 and 71 locations from all over the world, correspondingly. The vaccines of other viruses that belong to the Poxviridae family were recommended for monkeypox. There is no licensed vaccine available for monkeypox that originated from monkeypox virus. In the present study, using the reverse vaccinology approach we have performed whole proteome analysis of monkeypox virus to screen out the potential antigenic proteins that can be used as vaccine candidates. We have also designed 12 B cell epitopes-based vaccine candidates using immunoinformatics approach. We have found a total 15 potential antigenic proteins out of which 14 antigens are novel and can be used for further vaccine development against monkeypox. We have performed the physicochemical properties, antigenic, immunogenic and allergenicity prediction of the designed vaccine candidates MPOXVs (MPOXV1-MPOXV12). Further, we have performed molecular docking, in silico immune simulation and cloning of MPOXVs. All MPOXVs are potential vaccine candidate that can potentially activate the innate, cellular, and humoral immune response. However, further experimental validation is required before moving to clinical trials. This is the first oral vaccine reported for monkeypox virus derived from monkeypox proteins. |
| An integrative reverse vaccinology, immunoinformatic, docking and simulation approaches towards designing of multi-epitopes based vaccine against monkeypox virus | (Ullah et al., 2023) | Journal of Biomolecular Structure and Dynamics | 10.1080/07391102.2022.2125441 | Monkeypox is a viral zoonotic disease that is caused by the monkeypox virus (MPXV) and is mainly transmitted to human through close contact with an infected person, animal, or fomites which is contaminated by the virus. In the present research work, reverse vaccinology and several other bioinformatics and immunoinformatics tools were utilized to design multi-epitopes-based vaccine against MPXV by exploring three probable antigenic extracellular proteins: cupin domain-containing protein, ABC transporter ATP-binding protein and DUF192 domain-containing protein. Both cellular and humoral immunity induction were the main concerning qualities of the vaccine construct, hence from selected proteins both B and T-cells epitopes were predicted. Antigenicity, allergenicity, toxicity, and water solubility of the predicted epitopes were assessed and only probable antigenic, non-allergic, non-toxic and good water-soluble epitopes were used in the multi-epitopes vaccine construct. The developed vaccine was found to be potentially effective against MPXV and to be highly immunogenic, cytokine-producing, antigenic, non-toxic, non-allergenic, and stable. Additionally, to increase stability and expression efficiency in the host E. coli, disulfide engineering, codon adaptation, and in silico cloning were employed. Molecular docking and other biophysical approaches were utilized to evaluate the binding mode and dynamic behavior of the vaccine construct with TLR-2, TLR-4, and TLR-8. The outcomes of the immune simulation demonstrated that both B and T cells responded more strongly to the vaccination component. The detailed in silico analysis concludes that the proposed vaccine will induce a strong immune response against MPXV infection, making it a promising target for additional experimental trials. |
| Designing multi-epitope monkeypox virus-specific vaccine using immunoinformatics approach | (Zaib et al., 2023) | Journal of Infection and Public Health | 10.1016/j.jiph.2022.11.033 | Background: Monkeypox virus is an enveloped DNA virus that belongs to Poxviridae family. The virus is transmitted from rodents to primates via infected body fluids, skin lesions, and respiratory droplets. After being infected with virus, the patients experience fever, myalgia, maculopapular rash, and fluid-filled blisters. It is necessary to differentiate monkeypox virus from other poxviruses during diagnosis which can be appropriately envisioned via DNA analysis from swab samples. During small outbreaks, the virus is treated with therapies administered in other orthopoxviruses infections and does not have its own specific therapy and vaccine. Consequently, in this article, two potential peptides have been designed. Methods: For the purpose of designing a vaccine, protein sequences were retrieved followed by the prediction of B- and T-cell epitopes. Afterward, vaccine structures were predicted which were docked with toll-like receptors. The docked complexes were analyzed with iMODS. Moreover, vaccine constructs nucleotide sequences were optimized and expressed in silico. Results: COP-B7R vaccine construct (V1) has antigenicity score of 0.5400, instability index of 29.33, z-score of − 2.11-, and 42.11% GC content whereas COP-A44L vaccine construct (V2) has an antigenicity score of 0.7784, instability index of 23.33, z-score of − 0.61, and 48.63% GC content. It was also observed that COP-A44L can be expressed as a soluble protein in Escherichia coli as compared to COP-B7R which requires a different expression system. Conclusion: The obtained results revealed that both vaccine constructs show satisfactory outcomes after in silico investigation and have significant potential to prevent the monkeypox virus. However, COP-A44L gave better results. |
| Design of a novel multiple epitope-based vaccine: an immunoinformatics approach to combat monkeypox | (Hayat et al., 2023) | Journal of Biomolecular Structure and Dynamics | 10.1080/07391102.2022.2141887 | Monkeypox virus is an infectious agent that causes fever, Pneumonitis encephalitis, rash, lymphadenopathy and bacterial infection. The current outbreak of monkeypox has reawakened the global health concern. In the current situation of increasing viral infection, no vaccine or drug is available for monkeypox. Thus, there is an urgent need for viable vaccine development to prevent viral transmission by boosting human immunity. Herein, using immunoinformatics approaches, a multi-epitope vaccine was constructed for the Monkeypox virus. In this connection, B-Cell and T-cell epitopes were identified and joined with the help of adjutants and linkers. The vaccine construct was selected based on promising vaccine candidates and immunogenic potential. Further epitopes were selected based on antigenicity score, non-allergenicity and good immunological properties. Molecular docking reveals strong interactions between TLR-9 and the predicted vaccine construct. Finally, molecular dynamics simulations were performed to evaluate the stability and compactness of the constructed vaccine. The MD simulation results demonstrated the significant stability of the polypeptide vaccine construct. The predicted vaccine represented good stability, expression, immunostimulatory capabilities and significant solubility. Design vaccine was verified as efficient in different computer-based immune response investigations. Additionally, the constructed vaccine also represents a good population coverage in computer base analysis. |
| Probing the proteome of mpox virus for in silico design of a multiepitope vaccine | (Danazumi et al., 2023) | Future Drug Discovery | 10.4155/fdd-2023-0013 | Aim: This work aims to contribute toward development of preventive measures for the control of monkeypox (mpox) virus disease through computational design of a multiepitope vaccine. Methods: To accomplish this, we employed a robust immunoinformatics approach to design a putative chimeric vaccine candidate from 18 viral transmembrane proteins. Results: The resulting chimeric vaccine candidate is a 76.4 kDa protein containing 687 amino acids with an estimated isoelectric point of 9.39. In addition, it was predicted to adopt a stable 3D conformation that harbors discontinuous B-cell epitopes and strongly interacts with key immune receptors. Conclusion: The designed hypothetical antigen is a valuable addition to the collection of prospective vaccine candidates for future development and trials against the reemerging mpox disease. |
| Immunoinformatic-guided novel mRNA vaccine designing to elicit immunogenic responses against the endemic Monkeypox virus | (Aiman et al., 2024) | Journal of Biomolecular Structure and Dynamics | 10.1080/07391102.2023.2233627 | Monkeypox virus (MPXV) is an orthopoxvirus, causing zoonotic infections in humans with smallpox-like symptoms. The WHO reported MPXV cases in May 2022 and the outbreak caused significant morbidity threats to immunocompromised individuals and children. Currently, no clinically validated therapies are available against MPXV infections. The present study is based on immunoinformatics approaches to design mRNA-based novel vaccine models against MPXV. Three proteins were prioritized based on high antigenicity, low allergenicity, and toxicity values to predict T- and B-cell epitopes. Lead T- and B-cell epitopes were used to design vaccine constructs, linked with epitope-specific linkers and adjuvant to enhance immune responses. Additional sequences, including Kozak sequence, MITD sequence, tPA sequence, Goblin 5’, 3’ UTRs, and a poly(A) tail were added to design stable and highly immunogenic mRNA vaccine construct. High-quality structures were predicted by molecular modeling and 3D-structural validation of the vaccine construct. Population coverage and epitope-conservancy speculated broader protection of designed vaccine model against multiple MPXV infectious strains. MPXV-V4 was eventually prioritized based on its physicochemical and immunological parameters and docking scores. Molecular dynamics and immune simulations analyses predicted significant structural stability and binding affinity of the top-ranked vaccine model with immune receptors to elicit cellular and humoral immunogenic responses against the MPXV. The pursuance of experimental and clinical follow-up of these prioritized constructs may lay the groundwork to develop safe and effective vaccine against MPXV. |
| Discovering conserved epitopes of Monkeypox: Novel immunoinformatic and machine learning approaches | (Izadi et al., 2024) | Heliyon | 10.1016/j.heliyon.2024.e24972 | The Monkeypox virus, an Orthopoxvirus with zoonotic origins, has been responsible for a growing number of human infections reminiscent of smallpox since May 2022, as reported by the World Health Organization. As of now, there are no established medical treatments for managing Monkeypox infections. In this study, we used machine learning to select conserved epitopes. Proteins were determined using Reverse Vaccinology and Gene Ontology subcellular localization, and their epitopes were predicted. NextClade was used to calculate the number of mutations in each amino acid position using 2433 Monkeypox sequences. The Unsupervised Nearest Neighbor machine learning algorithm and ideal matrix [0 0] were used to calculate the conservancy score of epitopes. Six proteins were determined for epitope prediction. Finally, 47 MHC-I epitopes, 5 MHC-II epitopes, and 10 Linear B cell epitopes were discovered. Our method can select epitopes for vaccine design to prevent viruses with accelerated evolution and high mutation rate. |
| A novel multi-epitope peptide vaccine targeting immunogenic antigens of Ebola and monkeypox viruses with potential of immune responses provocation in silico | (Mahmoodi et al., 2024) | Biotechnology and Applied Biochemistry | 10.1002/bab.2646 | The emergence or reemergence of monkeypox (Mpox) and Ebola virus (EBOV) agents causing zoonotic diseases remains a huge threat to human health. Our study aimed at designing a multi-epitope vaccine (MEV) candidate to target both the Mpox and EBOV agents using immunoinformatics tools. Viral protein sequences were retrieved, and potential nonallergenic, nontoxic, and antigenic epitopes were obtained. Next, cytotoxic and helper T-cell (CTL and HTL, respectively) and B-cell (BCL) epitopes were predicted, and those potential epitopes were fused utilizing proper linkers. The in silico cloning and expression processes were implemented using Escherichia coli K12. The immune responses were prognosticated using the C-ImmSim server. The MEV construct (29.53 kDa) included four BCL, two CTL, and four HTL epitopes and adjuvant. The MEV traits were pertinent in terms of antigenicity, non-allergenicity, nontoxicity, physicochemical characters, and stability. The MEV candidate was also highly expressed in E. coli K12. The strong affinity of MEV-TLR3 was confirmed using molecular docking and molecular dynamics simulation analyses. Immune simulation analyses unraveled durable activation and responses of cellular and humoral arms alongside innate immune responses. The designed MEV candidate demonstrated appropriate traits and was promising in the prediction of immune responses against both Mpox and EBOV agents. Further experimental assessments of the MEV are required to verify its efficacy. |
| Designing a smallpox B-cell and T-cell multi-epitope subunit vaccine using a comprehensive immunoinformatics approach | (Yu et al., 2024) | Microbiology Spectrum | 10.1128/spectrum.00465-24 | Smallpox is a highly contagious human disease caused by the variola virus. Although the disease was eliminated in 1979 due to its highly contagious nature and historical pathogenicity, with a mortality rate of up to 30%, this virus is an important candidate for biological weapons. Currently, vaccines are the critical measures to prevent this virus infection and spread. In this study, we designed a peptide vaccine using immunoinformatics tools, which have the potential to activate human immunity against variola virus infection efficiently. The design of peptides derives from vaccine-candidate proteins showing protective potential in vaccinia WR strains. Potential non-toxic and nonallergenic T-cell and B-cell binding and cytokine-inducing epitopes were then screened through a priority prediction using special linkers to connect B-cell epitopes and T-cell epitopes, and an appropriate adjuvant was added to the vaccine construction to enhance the immunogenicity of the peptide vaccine. The 3D structure display, docking, and free energy calculation analysis indicate that the binding affinity between the vaccine peptide and Toll-like receptor 3 is high, and the vaccine receptor complex is highly stable. Notably, the vaccine we designed is obtained from the protective protein of the vaccinia and combined with preventive measures to avoid side effects. This vaccine is highly likely to produce an effective and safe immune response against the variola virus infection in the body. IMPORTANCE In this work, we designed a vaccine with a cluster of multiple T-cell/B-cell epitopes, which should be effective in inducing systematic immune responses against variola virus infection. Besides, this work also provides a reference in vaccine design for preventing monkeypox virus infection, which is currently prevalent. |
| In silico designing a novel TLR4-mediating multiepitope vaccine against monkeypox via advanced immunoinformatics and bioinformatics approaches | (Lahimchi et al., 2024) | Journal of Biomolecular Structure and Dynamics | 10.1080/07391102.2023.2203253 | Monkeypox virus is a member of the Poxviridae family, which causes monkeypox zoonotic disease. Since July 2022, the prevention of monkeypox have become more considerable due to the new outbreak, making it a global concern. Therefore, we used an in silico-based method, including immunoinformatics, bioinformatics, molecular docking, and gene cloning approaches to design a novel multiepitope vaccine against monkeypox. Three immunogenic envelope proteins of monkeypox virus, including G10R, E8L, and A30L, were selected to predict appropriate immune system stimulator epitopes. The A30L is common between smallpox and monkeypox virus, so the proposed vaccine may be effective against smallpox too. There is no evidence of allergenicity and toxicity of the vaccine epitopes. To boost the immunogenicity of the designed vaccine, we used the helper epitope of PADRE and RS01as adjuvants. Furthermore, some linkers are used to link epitopes and adjuvants together. The physicochemical futures of the designed vaccine were assessed. The tertiary structure of the vaccine was modeled and then refined to improve its structure and physicochemical properties. To analyze the vaccine construct and TLR4 complex affinity, they were docked to gather. Besides, the vaccine was cloned into E.coli. pET-21b(+) plasmid to reveal that it can be expressed and stimulate the immune system. Immune stimulation evaluation showed that the candidate vaccine could induce the production of IgM, IgG1, and IgG2 antibodies. Overall, we suggested an effective vaccine candidate against monkeypox. However, Future studies and clinical trials should be done to ensure the efficacy and safety of this vaccine. |
| In-silico formulation of a next-generation polyvalent vaccine against multiple strains of monkeypox virus and other related poxviruses | (Moin et al., 2024) | PLoS ONE | 10.1371/journal.pone.0300778 | Mpox (formerly known as monkeypox) virus and some related poxviruses including smallpox virus pose a significant threat to public health, and effective prevention and treatment strategies are needed. This study utilized a reverse vaccinology approach to retrieve conserved epitopes for monkeypox virus and construct a vaccine that could provide cross-protection against related viruses with similar antigenic properties. The selected virulent proteins of monkeypox virus, MPXVgp165, and Virion core protein P4a, were subjected to epitope mapping for vaccine construction. Two vaccines were constructed using selected T cell epitopes and B cell epitopes with PADRE and human beta-defensins adjuvants conjugated in the vaccine sequence. Both constructs were found to be highly antigenic, non-allergenic, nontoxic, and soluble, suggesting their potential to generate an adequate immune response and be safe for humans. Vaccine construct 1 was selected for molecular dynamic simulation studies. The simulation studies revealed that the TLR8-vaccine complex was more stable than the TLR3-vaccine complex. The lower RMSD and RMSF values of the TLR8 bound vaccine compared to the TLR3 bound vaccine suggested better stability and consistency of hydrogen bonds. The Rg values of the vaccine chain bound to TLR8 indicated overall stability, whereas the vaccine chain bound to TLR3 showed deviations throughout the simulation. These results suggest that the constructed vaccine could be a potential preventive measure against monkeypox and related viruses however, further experimental validation is required to confirm these findings. |
| Design and computational evaluation of a novel multi-epitope hybrid vaccine against monkeypox virus: Potential targets and immunogenicity assessment for pandemic preparedness | (Yaseen et al., 2024) | Biologicals | 10.1016/j.biologicals.2024.101770 | Monkeypox is a type of DNA-enveloped virus that belongs to the orthopoxvirus family, closely related to the smallpox virus. It can cause an infectious disease in humans known as monkeypox disease. Although there are multiple drugs and vaccines designed to combat orthopoxvirus infections, with a primary focus on smallpox, the recent spread of the monkeypox virus to over 50 countries have ignited a mounting global concern. This unchecked viral proliferation has raised apprehensions about the potential for a pandemic corresponding to the catastrophic impact of COVID-19. This investigation explored the structural proteins of monkeypox virus as potential candidates for designing a novel hybrid multi-epitope vaccine. The epitopes obtained from the selected proteins were screened to ensure their non-allergenicity, non-toxicity, and antigenicity to trigger T and B-cell responses. The interaction of the vaccine with toll-like receptor-3 (TLR-3) and major histocompatibility complexes (MHCs) was assessed using Cluspro 2.0. To establish the reliability of the docked complexes, a comprehensive evaluation was conducted using Immune and MD Simulations and Normal Mode Analysis. However, to validate the computational results of this study, additional in-vitro and in-vivo research is essential. |
| Design of multi-epitope chimeric vaccine against Monkeypox virus and SARS-CoV-2: A vaccinomics perspective | (Al-Madhagi et al., 2024) | Journal of Cellular and Molecular Medicine | 10.1111/jcmm.18452 | The current era we experience is full with pandemic infectious agents that no longer threatens the major local source but the whole globe. Almost the most emerging infectious agents are severe acute respiratory syndrome coronavirus-2 (SARS CoV-2), followed by monkeypox virus (MPXV). Since no approved antiviral drugs nor licensed active vaccines are yet available, we aimed to utilize immunoinformatics approach to design chimeric vaccine against the two mentioned viruses. This is the first study to deal with design divalent vaccine against SARS-CoV-2 and MPXV. ORF8, E and M proteins from Omicron SARS-CoV-2 and gp182 from MPXV were used as the protein precursor from which multi-epitopes (inducing B-cell, helper T cells, cytotoxic T cells and interferon-ɣ) chimeric vaccine was contrived. The structure of the vaccine construct was predicted, validated, and docked to toll-like receptor-2 (TLR-2). Moreover, its sequence was also used to examine the immune simulation profile and was then inserted into the pET-28a plasmid for in silico cloning. The vaccine construct was probable antigen (0.543) and safe (non-allergen) with strong binding energy to TLR-2 (−1169.8 kcal/mol) and found to have significant immune simulation profile. In conclusion, the designed chimeric vaccine was potent and safe against SARS-CoV-2 and MPXV, which deserves further consideration. |
| An immuno-informatics approach for annotation of hypothetical proteins and multi-epitope vaccine designed against the Mpox virus | (Ahmed et al., 2024) | Journal of Biomolecular Structure and Dynamics | 10.1080/07391102.2023.2239921 | A worrying new outbreak of Monkeypox (Mpox) in humans is caused by the Mpox virus (MpoxV). The pathogen has roughly 28 hypothetical proteins of unknown structure, function, and pathogenicity. Using reliable bioinformatics tools, we attempted to analyze the MpoxV genome, identify the role of hypothetical proteins (HPs), and design a potential candidate vaccine. Out of 28, we identified seven hypothetical proteins using multi-server validation with high confidence for the occurrence of conserved domains. Their physical, chemical, and functional characterizations, including molecular weight, theoretical isoelectric point, 3D structures, GRAVY value, subcellular localization, functional motifs, antigenicity, and virulence factors, were performed. We predicted possible cytotoxic T cell (CTL), helper T cell (HTL) and linear and conformational B cell epitopes, which were combined in a 219 amino acid multiepitope vaccine with human β defensin as a linker. This multi-epitopic vaccine was structurally modelled and docked with toll-like receptor-3 (TLR-3). The dynamical stability of the vaccine-TLR-3 docked complexes exhibited stable interactions based on RMSD and RMSF tests. Additionally, the modelled vaccine was cloned in-silico in an E. coli host to check the appropriate expression of the final vaccine built. Our results might conform to an immunogenic and safe vaccine, which would require further experimental validation. |
| Immunoinformatic Approach for Rational Identification of Immunogenic Peptides Against Host Entry and/or Exit Mpox Proteins and Potential Multiepitope Vaccine Construction | (de Araújo et al., 2024) | Journal of Infectious Diseases | 10.1093/infdis/jiad443 | COVID-19 has intensified humanity’s concern about the emergence of new pandemics. Since 2018, epidemic outbreaks of the mpox virus have become worrisome. In June 2022, the World Health Organization declared the disease a global health emergency, with 14 500 cases reported by the Centers for Disease Control and Prevention in 60 countries. Therefore, the development of a vaccine based on the current virus genome is paramount in combating new cases. In view of this, we hypothesized the obtainment of rational immunogenic peptides predicted from proteins responsible for entry of the mpox virus into the host (A17L, A26L/A30L, A33R, H2R, L1R), exit (A27L, A35R, A36R, C19L), and both (B5R). To achieve this, we aligned the genome sequencing data of mpox virus isolated from an infected individual in the United States in June 2022 (ON674051.1) with the reference genome dated 2001 (NC_003310.1) for conservation analysis. The Immune Epitope Database server was used for the identification and characterization of the epitopes of each protein related to major histocompatibility complex I or II interaction and recognition by B-cell receptors, resulting in 138 epitopes for A17L, 233 for A28L, 48 for A33R, 77 for H2R, 77 for L1R, 270 for A27L, 72 for A35R, A36R, 148 for C19L, and 276 for B5R. These epitopes were tested in silico for antigenicity, physicochemical properties, and allergenicity, resulting in 51, 40, 10, 34, 38, 57, 25, 7, 47, and 53 epitopes, respectively. Additionally, to select an epitope with the highest promiscuity of binding to major histocompatibility complexes and B-cell receptor simultaneously, all epitopes of each protein were aligned, and the most repetitive and antigenic regions were identified. By classifying the results, we obtained 23 epitopes from the entry proteins, 16 from the exit proteins, and 7 from both. Subsequently, 1 epitope from each protein was selected, and all 3 were fused to construct a chimeric protein that has potential as a multiepitope vaccine. The constructed vaccine was then analyzed for its physicochemical, antigenic, and allergenic properties. Protein modeling, molecular dynamics, and molecular docking were performed on Toll-like receptors 2, 4, and 8, followed by in silico immune simulation of the vaccine. Finally, the results indicate an effective, stable, and safe vaccine that can be further tested, especially in vitro and in vivo, to validate the findings demonstrated in silico. |
| Formulation of next-generation polyvalent vaccine candidates against three important poxviruses by targeting DNA-dependent RNA polymerase using an integrated immunoinformatics and molecular modeling approach | (Kumar et al., 2024) | Journal of Infection and Public Health | 10.1016/j.jiph.2024.102470 | Background: Poxviruses comprise a group of large double-stranded DNA viruses and are known to cause diseases in humans, livestock animals, and other animal species. The Mpox virus (MPXV; formerly Monkeypox), variola virus (VARV), and volepox virus (VPXV) are among the prevalent poxviruses of the Orthopoxviridae genera. The ongoing Mpox infectious disease pandemic caused by the Mpox virus has had a major impact on public health across the globe. To date, only limited repurposed antivirals and vaccines are available for the effective treatment of Mpox and other poxviruses that cause contagious diseases. Methods: The present study was conducted with the primary goal of formulating multi-epitope vaccines against three evolutionary closed poxviruses i.e., MPXV, VARV, and VPXV using an integrated immunoinformatics and molecular modeling approach. DNA-dependent RNA polymerase (DdRp), a potential vaccine target of poxviruses, has been used to determine immunodominant B and T-cell epitopes followed by interactions analysis with Toll-like receptor 2 at the atomic level. Results: Three multi-epitope vaccine constructs, namely DdRp_MPXV (V1), DdRp_VARV (V2), and DdRp_VPXV (V3) were designed. These vaccine constructs were found to be antigenic, non-allergenic, non-toxic, and soluble with desired physicochemical properties. Protein-protein docking and interaction profiling analysis depicts a strong binding pattern between the targeted immune receptor TLR2 and the structural models of the designed vaccine constructs, and manifested a number of biochemical bonds (hydrogen bonds, salt bridges, and non-bonded contacts). State-of-the-art all-atoms molecular dynamics simulations revealed highly stable interactions of vaccine constructs with TLR2 at the atomic level throughout the simulations on 300 nanoseconds. Additionally, the outcome of the immune simulation analysis suggested that designed vaccines have the potential to induce protective immunity against targeted poxviruses. Conclusions: Taken together, formulated next-generation polyvalent vaccines were found to have good efficacy against closely related poxviruses (MPXV, VARV, and VPXV) as demonstrated by our extensive immunoinformatics and molecular modeling evaluations; however, further experimental investigations are still needed. |
| Exploring computational approaches to design mRNA Vaccine against vaccinia and Mpox viruses | (Oladipo et al., 2024) | Immunity, Inflammation and Disease | 10.1002/iid3.1360 | Background: Messenger RNA (mRNA) vaccines emerged as a powerful tool in the fight against infections. Unlike traditional vaccines, this unique type of vaccine elicits robust and persistent innate and humoral immune response with a unique host cell-mediated pathogen gene expression and antigen presentation. Methods: This offers a novel approach to combat poxviridae infections. From the genome of vaccinia and Mpox viruses, three key genes (E8L, E7R, and H3L) responsible for virus attachment and virulence were selected and employed for designing the candidate mRNA vaccine against vaccinia and Mpox viral infection. Various bioinformatics tools were employed to generate (B cell, CTL, and HTL) epitopes, of which 28 antigenic and immunogenic epitopes were selected and are linked to form the mRNA vaccine construct. Additional components, including a 5′ cap, 5′ UTR, adjuvant, 3′ UTR, and poly(A) tail, were incorporated to enhance stability and effectiveness. Safety measures such as testing for human homology and in silico immune simulations were implemented to avoid autoimmunity and to mimics the immune response of human host to the designed mRNA vaccine, respectively. The mRNA vaccine's binding affinity was evaluated by docking it with TLR-2, TLR-3, TLR-4, and TLR-9 receptors which are subsequently followed by molecular dynamics simulations for the highest binding one to predict the stability of the binding complex. Results: With a 73% population coverage, the mRNA vaccine looks promising, boasting a molecular weight of 198 kDa and a molecular formula of C8901H13609N2431O2611S48 and it is said to be antigenic, nontoxic and nonallergic, making it safe and effective in preventing infections with Mpox and vaccinia viruses, in comparison with other insilico-designed vaccine for vaccinia and Mpox viruses. Conclusions: However, further validation through in vivo and in vitro techniques is underway to fully assess its potential. |
| In silico development of a novel anti-mutation, multi-epitope mRNA vaccine against MPXV variants of emerging lineage and sub-lineages by using immunoinformatics approaches | (Tan et al., 2024) | Journal of Biomolecular Structure and Dynamics | 10.1080/07391102.2024.2325109 | Over the past year, an unexpected surge in human monkeypox (hMPX) cases has been observed. This outbreak differs from previous ones, displaying distinct epidemiological characteristics and transmission patterns, believed to be influenced by a newly emerging monkeypox virus (MPXV) lineage. Notably, this emerging MPXV lineage has exhibited several non-synonymous mutations, some of which are linked to immunomodulatory activities and antigenic characteristics that aid in host detection. However, specific treatments or vaccines for human monkeypox are currently lacking. Hence, we aim to develop a multi-epitope mRNA vaccine by using immunoinformatics approaches against the MPXV, particularly its emerging variants. Six proteins (A29L, A35R, B6R, M1R, H3L, and E8L) were chosen for epitope and mutation site identification. Seventeen top-performing epitopes and eight epitopes containing mutation sites were selected and combined with adjuvants, the PADRE sequence, and linkers for vaccine development. The molecular and physical properties of the designed vaccine (WLmpx) were favorable. Immunological characteristics of WLmpx were assessed through molecular docking, molecular dynamics (MD) simulations, and immune simulations. Finally, the vaccine sequence was utilized to formulate an mRNA-based vaccine. The informatics-based predicted results indicated that the designed vaccine exhibits significant potential in eliciting high-level humoral and cellular immune responses, but further validation through in vivo and vitro studies is warranted. |
| Construction of Peptide Vaccine Candidate Based on β-Cell Epitopes of Indonesian Monkeypox Virus (MPXV) Virulence Protein:A Reverse Vaccinology | (Kharisma et al., 2024) | Research Journal of Pharmacy and Technology | 10.52711/0974-360X.2024.00045 | Infection with a DNA virus called monkeypox virus (MPXV) in humans has been identified in the Congo since 1970. Antiviral drugs are not effective for preventing MPXV infection. MPXV infection cases in Indonesia are very low but MPXV has the potential to become a global pandemic so it is very important to do prevention such as vaccine development. This study aims to construct a B cell epitope-based peptide vaccine candidate in Indonesian MPXV through an in silico approach. The development of the MPXV vaccine can be performed through a computational approach for preliminary studies. In silico-based construction of vaccines using B cell epitopes, antigenicity, allergenicity, docking, and molecular dynamics analysis have been used by researchers and scientists in solving viral infection cases. We recommend Pep A and Pep D as vaccine candidates because they allow recognition by B cells, antigenic peptides, non-allergenic and non-toxin. Peptide vaccine candidate can trigger B-cell activation to produce IgM isotype-specific antibodies through BCR interaction. In summary, the results of this study can be used for an initial study of MPXV vaccine development in Indonesia. |
| An immunoinformatic approach for developing a multi-epitope subunit vaccine against Monkeypox virus | (Nayak et al., 2024) | In Silico Pharmacology | 10.1007/s40203-024-00220-5 | An in-silico approach was implemented to develop a multi-epitope subunit vaccine construct against the recent outbreak of the Monkeypox virus. The contribution of 10 different antigenic proteins based on their antigenicity led to the selection of 10 HTL, 9 CTL, and 6 BCL epitopes. The construct was further investigated for its allergenicity, antigenicity, and physio-chemical properties using servers such as AllerTOP and Allergen FP, VaxiJen and ANTIGENPro, and ProtParam respectively. The secondary structure of the vaccine was predicted using the SOPMA server followed by I-TASSER for the 3D structure. After refinement and validation of structural stability of the modelled vaccine, a molecular docking assay was implemented to study the interaction of the known TLR4 receptor with that of the constructed vaccine using the ClusPro server. The docked vaccine and TLR4 receptor were studied using the molecular dynamics (MD) simulation to validate the stability of the complex. After codon optimization the cDNA was constructed and in-silico cloning of the vaccine construct was carried out. The vaccine was also subjected to computational immune assay which predicted a powerful immune response against the Monkeypox virus validating that the developed multi-epitope vaccine construct can be a potent vaccine candidate. |
| EPITOPE BASED VACCINE DESIGN AGAINST MONKEY POX VIRUS BY DEPLOYING LATEST IMMUNOINFORMATICS APPROACH | Joshi & Kumar | Patent from India | IN202321006081 | Out Invention “Epitope based Vaccine design against Monkey pox virus by deploying latest Immunoinformatics approach.” Is a arising monkey pox infection (MPXV) is a zoonotic orthopoxvirus that causes contaminations in people like smallpox. Since May 2022, instances of monkeypox (MPX) have been progressively detailed by the World Wellbeing Association (WHO) around the world. At present, there are no clinically approved medicines for MPX contaminations. In this review, an Immunoinformatics approach was utilized to distinguish potential immunization focuses against MPXV. A sum of 190 MPXV-2022 proteins were recovered from the Vi-PR data set and exposed to different investigations including antigenicity, allergen city, poisonousness, dissolvability, IFN-?, and harmfulness. Three external layer and extracellular proteins were chosen in view of their separate boundaries to anticipate B-cell and Immune system microorganism epitopes. The epitopes are moderated among various types of MPXV and the populace inclusion is 100 percent around the world, which will give more extensive assurance against different kinds of the infection universally. Nine covering MHC-I, MHC-II, and B-cell epitopes were chosen to plan multi-epitope antibody develops connected with reasonable linkers in mix with various adjuvants to upgrade the safe reactions of the immunization develops. Atomic demonstrating and underlying approval guaranteed excellent 3D designs of immunization develops. In light of different immunological and physiochemical properties and docking scores, MPXV-V2 was chosen for additional examination. In silico cloning uncovered an elevated degree of quality articulation for the MPXV-V2 immunization inside the bacterial articulation framework. Resistant and MD reproductions affirmed the sub-atomic strength of the MPXV-V2 build, with high safe reactions inside the host cell. These outcomes might support the advancement of exploratory immunizations against MPXV with expanded intensity and further developed security. |

**References**

Abdi, S. A. H., Ali, A., Sayed, S. F., Abutahir, Ali, A., and Alam, P. (2022). Multi-Epitope-Based Vaccine Candidate for Monkeypox: An In Silico Approach. *Vaccines* 10 (9), 1564. doi: 10.3390/vaccines10091564

Ahmed, M. H., Samia, N. S. N., Singh, G., Gupta, V., Mishal, M. F. M., Hossain, A., et al. (2024). An immuno-informatics approach for annotation of hypothetical proteins and multi-epitope vaccine designed against the Mpox virus. *J. Biomol. Struct. Dyn.* 42 (10), 5288–5307. doi: 10.1080/07391102.2023.2239921

Aiman, S., Alhamhoom, Y., Ali, F., Rahman, N., Rastrelli, L., Khan, A., et al. (2022). Multi-epitope chimeric vaccine design against emerging Monkeypox virus via reverse vaccinology techniques- a bioinformatics and immunoinformatics approach. *Front. Immunol.* 13, 985450. doi: 10.3389/fimmu.2022.985450

Aiman, S., Ali, Y., Malik, A., Alkholief, M., Ahmad, A., Akhtar, S., et al. (2024). Immunoinformatic-guided novel mRNA vaccine designing to elicit immunogenic responses against the endemic Monkeypox virus. *J. Biomol. Struct. Dyn.* 42 (12), 6292–6306. doi: 10.1080/07391102.2023.2233627

Akhtar, N., Kaushik, V., Grewal, R. K., Wani, A. K., Suwattanasophon, C., Choowongkomon, K., et al. (2022). Immunoinformatics-Aided Design of a Peptide Based Multiepitope Vaccine Targeting Glycoproteins and Membrane Proteins against Monkeypox Virus. *Viruses* 14 (11), 2374. doi: 10.3390/v14112374

Al-Madhagi, H., Kanawati, A., and Tahan, Z. (2024). Design of multi-epitope chimeric vaccine against Monkeypox virus and SARS-CoV-2: A vaccinomics perspective. *J. Cell. Mol. Med.* 28 (10), e18452. doi: 10.1111/jcmm.18452

Alsaiari, A. A., Hakami, M. A., Alotaibi, B. S., Alkhalil, S. S., Alkhorayef, N., Khan, K., et al. (2023). Delineating multi-epitopes vaccine designing from membrane protein CL5 against all monkeypox strains: a pangenome reverse vaccinology approach. *J. Biomol. Struct. Dyn.* 42 (16), 8385–8406. doi: 10.1080/07391102.2023.2248301

Aziz, S., Almajhdi, F. N., Waqas, M., Ullah, I., Salim, M. A., Khan, N. A., et al. (2022). Contriving multi-epitope vaccine ensemble for monkeypox disease using an immunoinformatics approach. *Front. Immunol.* 13, 1004804. doi: 10.3389/fimmu.2022.1004804

Bai, S., Cui, Y., Liao, Q., Yi, H., Liao, Z., Zhang, G., et al. (2025). Enhanced Immunogenicity and Affinity with A35R-Fc-Based Chimeric Protein Compared to MPXV A35R Protein. *Viruses* 17 (1), 116. doi: 10.3390/v17010116

Bhattacharya, K., Shamkh, I. M., Khan, M. S., Lotfy, M. M., Nzeyimana, J. B., Abutayeh, R. F., et al. (2022a). Multi-Epitope Vaccine Design against Monkeypox Virus via Reverse Vaccinology Method Exploiting Immunoinformatic and Bioinformatic Approaches. *Vaccines* 10 (12), 2010. doi: 10.3390/vaccines10122010

Bhattacharya, M., Chatterjee, S., Nag, S., Dhama, K., and Chakraborty, C. (2022b). Designing, characterization, and immune stimulation of a novel multi-epitopic peptide-based potential vaccine candidate against monkeypox virus through screening its whole genome encoded proteins: An immunoinformatics approach. *Travel Med. Infect. Dis.* 50, 102481. doi: 10.1016/j.tmaid.2022.102481

Chen, L., Shang, C., Wang, Z., Zheng, M., Zhang, C., Li, D., et al. (2025). Chemical cross-linking facilitates antigen uptake and presentation and provides improved protection from Mpox with a dual-antigen subunit vaccine. *MedComm* 6 (1), e70045. doi: 10.1002/mco2.70045

Chi, H., Zhao, S. Q., Chen, R. Y., Suo, X. X., Zhang, R. R., Yang, W. H., et al. (2024). Rapid development of double-hit mRNA antibody cocktail against orthopoxviruses. *Signal Transduct. Target. Ther.* 9 (1), 69. doi: 10.1038/s41392-024-01766-8

Choudhury, A., Chandra, A., Dawoud, T. M., Nafidi, H. A., Singh, N., and Bourhia, M. (2023). Immunoinformatics and reverse vaccinology approach in designing a novel highly immunogenic multivalent peptide-based vaccine against the human monkeypox virus. *Front. Mol. Biosci.* 10, 1295817. doi: 10.3389/fmolb.2023.1295817

Cotter, C. A., Ignacio, M. A., Americo, J. L., Earl, P. L., Mucker, E. M., Frey, T. R., et al. (2024). Mpox mRNA-1769 vaccine inhibits orthopoxvirus replication at intranasal, intrarectal, and cutaneous sites of inoculation. *npj Vaccines* 9 (1), 256. doi: 10.1038/s41541-024-01052-2

Danazumi, A. U., Adepoju, O. A., Dibba, L. B., Ibrahim, B., Gital, S. I., Joseph, G. I., et al. (2023). Probing the proteome of mpox virus for *in silico* design of a multiepitope vaccine. *Futur. Drug Discov.* 5 (4), FDD86. doi: 10.4155/fdd-2023-0013

de Araújo, L. P., de Melo Santos, N. C., Corsetti, P. P., and de Almeida, L. A. (2024). Immunoinformatic Approach for Rational Identification of Immunogenic Peptides Against Host Entry and/or Exit Mpox Proteins and Potential Multiepitope Vaccine Construction. *J. Infect. Dis.* 229 (Supplement_2), S285–S292. doi: 10.1093/infdis/jiad443

Fang, Z., Monteiro, V. S., Renauer, P. A., Shang, X., Suzuki, K., Ling, X., et al. (2023). Polyvalent mRNA vaccination elicited potent immune response to monkeypox virus surface antigens. *Cell Res.* 33 (5), 407–410. doi: 10.1038/s41422-023-00792-5

Fantini, J., Chahinian, H., and Yahi, N. (2022). A Vaccine Strategy Based on the Identification of an Annular Ganglioside Binding Motif in Monkeypox Virus Protein E8L. *Viruses* 14 (11), 2531. doi: 10.3390/v14112531

Farzan, M., Farzan, M., Mirzaei, Y., Aiman, S., Azadegan-Dehkordi, F., and Bagheri, N. (2023). Immunoinformatics-based multi-epitope vaccine design for the re-emerging monkeypox virus. *Int. Immunopharmacol.* 123, 110725. doi: 10.1016/j.intimp.2023.110725

Freyn, A. W., Atyeo, C., Earl, P. L., Americo, J. L., Chuang, G. Y., Natarajan, H., et al. (2023). An mpox virus mRNA-lipid nanoparticle vaccine confers protection against lethal orthopoxviral challenge. *Sci. Transl. Med.* 15 (716), eadg3540. doi: 10.1126/scitranslmed.adg3540

Gao, F., He, C., Liu, M., Yuan, P., Tian, S., Zheng, M., et al. (2023). Cross-reactive immune responses to monkeypox virus induced by MVA vaccination in mice. *Virol. J.* 20 (1), 126. doi: 10.1186/s12985-023-02085-0

Grifoni, A., Zhang, Y., Tarke, A., Sidney, J., Rubiro, P., Reina-Campos, M., et al. (2022). Defining antigen targets to dissect vaccinia virus and monkeypox virus-specific T cell responses in humans. *Cell Host Microbe* 30 (12), 1662-1670.e4. doi: 10.1016/j.chom.2022.11.003

Hayat, C., Shahab, M., Khan, S. A., Liang, C., Duan, X., Khan, H., et al. (2023). Design of a novel multiple epitope-based vaccine: an immunoinformatics approach to combat monkeypox. *J. Biomol. Struct. Dyn.* 41 (19), 9344–9355. doi: 10.1080/07391102.2022.2141887

Hou, F., Zhang, Y., Liu, X., Murad, Y. M., Xu, J., Yu, Z., et al. (2023). mRNA vaccines encoding fusion proteins of monkeypox virus antigens protect mice from *vaccinia virus* challenge. *Nat. Commun.* 14 (1), 5925. doi: 10.1038/s41467-023-41628-5

Izadi, M., Mirzaei, F., Bagherzadeh, M. A., Ghiabi, S., and Khalifeh, A. (2024). Discovering conserved epitopes of Monkeypox: Novel immunoinformatic and machine learning approaches. *Heliyon* 10 (3), e24972. doi: 10.1016/j.heliyon.2024.e24972

Jahantigh, H. R., Shahbazi, B., Gouklai, H., Van der Weken, H., Gharibi, Z., Rezaei, Z., et al. (2023). Design peptide and multi-epitope protein vaccine candidates against monkeypox virus using reverse vaccinology approach: an in-silico study. *J. Biomol. Struct. Dyn.* 41 (23), 14398–14418. doi: 10.1080/07391102.2023.2201850

Jiang, F., Liu, Y., Xue, Y., Cheng, P., Wang, J., Lian, J., et al. (2023). Developing a multiepitope vaccine for the prevention of SARS-CoV-2 and monkeypox virus co-infection: A reverse vaccinology analysis. *Int. Immunopharmacol.* 115, 109728. doi: 10.1016/j.intimp.2023.109728

Jin, Y., Fayyaz, A., Liaqat, A., Khan, A., Alshammari, A., Wang, Y., et al. (2023). Proteomics-based vaccine targets annotation and design of subunit and mRNA-based vaccines for Monkeypox virus (MPXV) against the recent outbreak. *Comput. Biol. Med.* 159, 106893. doi: 10.1016/j.compbiomed.2023.106893

Khan, A., Wei, D. Q., and Suleman, M. (2023a). “Computational Vaccine Design for *Poxviridae* Family Viruses,” in *Computational Vaccine Design. Methods in Molecular Biology*, ed. P. A. Reche (New York, NY: Humana), 475–485. doi: 10.1007/978-1-0716-3239-0_31

Khan, S., Irfan, M., Hameed, A. R., Ullah, A., Abideen, S. A., Ahmad, S., et al. (2023b). Vaccinomics to design a multi-epitope-based vaccine against monkeypox virus using surface-associated proteins. *J. Biomol. Struct. Dyn.* 41 (20), 10859–10868. doi: 10.1080/07391102.2022.2158942

Kharisma, V. D., Ansori, A. N. M., Murtadlo, A. A. A., Widyananda, M. H., Ullah, M. E., Naw, S. W., et al. (2024). Construction of Peptide Vaccine Candidate Based on β-Cell Epitopes of Indonesian Monkeypox Virus (MPXV) Virulence Protein:A Reverse Vaccinology. *Res. J. Pharm. Technol.* 17 (1), 291–296. doi: 10.52711/0974-360X.2024.00045

Kong, T., Du, P., Ma, R., Wang, H., Ma, X., Lu, J., et al. (2024). Single-chain A35R-M1R-B6R trivalent mRNA vaccines protect mice against both mpox virus and vaccinia virus. *eBioMedicine* 109, 105392. doi: 10.1016/j.ebiom.2024.105392

Kumar, A., Dutt, M., Dehury, B., Martinez, G. S., Singh, K. P., and Kelvin, D. J. (2024). Formulation of next-generation polyvalent vaccine candidates against three important poxviruses by targeting DNA-dependent RNA polymerase using an integrated immunoinformatics and molecular modeling approach. *J. Infect. Public Health* 17 (7), 102470. doi: 10.1016/j.jiph.2024.102470

Lahimchi, M. R., Madanchi, H., Ahmadi, K., Shahbazi, B., and Yousefi, B. (2024). In silico designing a novel TLR4-mediating multiepitope vaccine against monkeypox *via* advanced immunoinformatics and bioinformatics approaches. *J. Biomol. Struct. Dyn.* 42 (4), 2094–2110. doi: 10.1080/07391102.2023.2203253

Li, E., Gong, Q., Zhang, J., Guo, X., Xie, W., Chen, D., et al. (2024a). An mpox quadrivalent mRNA vaccine protects mice from lethal vaccinia virus challenge. *Antiviral Res.* 230, 105974. doi: 10.1016/j.antiviral.2024.105974

Li, E., Yang, Q., Xie, W., Gong, Q., Guo, X., Zhou, J., et al. (2025). An mpox quadrivalent mRNA vaccine elicits sustained and protective immunity in mice against lethal vaccinia virus challenge. *Emerg. Microbes Infect.* 14 (1), 2447619. doi: 10.1080/22221751.2024.2447619

Li, J., Li, X., Dong, J., Wei, J., Guo, X., Wang, G., et al. (2024b). Enhanced Immune Responses in Mice by Combining the Mpox Virus B6R-Protein and Aluminum Hydroxide-CpG Vaccine Adjuvants. *Vaccines* 12 (7), 776. doi: 10.3390/vaccines12070776

Li, M., Ren, Z., Wang, Y., Jiang, Y., Yang, M., Li, D., et al. (2023). Three neutralizing mAbs induced by MPXV A29L protein recognizing different epitopes act synergistically against orthopoxvirus. *Emerg. Microbes Infect.* 12 (2), 2223669. doi: 10.1080/22221751.2023.2223669

Mahmoodi, S., Amirzakaria, J. Z., and Ghasemian, A. (2024). A novel multi-epitope peptide vaccine targeting immunogenic antigens of Ebola and monkeypox viruses with potential of immune responses provocation in silico. *Biotechnol. Appl. Biochem.* doi: 10.1002/bab.2646

Mazumder, L., Hasan, M. R., Fatema, K., Begum, S., Azad, A. K., and Islam, M. A. (2023). Identification of B and T Cell Epitopes to Design an Epitope-Based Peptide Vaccine against the Cell Surface Binding Protein of Monkeypox Virus: An Immunoinformatics Study. *J. Immunol. Res.* 2023 (1), 2274415. doi: 10.1155/2023/2274415

Mishra, S., Rout, M., Panda, S., Singh, S. K., Sinha, R., Dehury, B., et al. (2023). An immunoinformatic approach towards development of a potent and effective multi-epitope vaccine against monkeypox virus (MPXV). *J. Biomol. Struct. Dyn.* 41 (21), 11714–11727. doi: 10.1080/07391102.2022.2163426

Moin, A. T., Rani, N. A., Patil, R. B., Robin, T. B., Ullah, M. A., Rahim, Z., et al. (2024). *In-silico* formulation of a next-generation polyvalent vaccine against multiple strains of monkeypox virus and other related poxviruses. *PLoS One* 19 (5), e0300778. doi: 10.1371/journal.pone.0300778

Montenegro Oyola, C. F., Noguera Rosero, B. A., and García-López, J. P. (2022). Análisis in silico de un candidato a vacuna multi-epítopo contra viruela del mono usando vaculonogía reversa. *Rev. la Asoc. Colomb. Ciencias Biológicas* 1 (34), 81–92. doi: 10.47499/revistaaccb.v1i34.265

Mucker, E. M., Freyn, A. W., Bixler, S. L., Cizmeci, D., Atyeo, C., Earl, P. L., et al. (2024). Comparison of protection against mpox following mRNA or modified vaccinia Ankara vaccination in nonhuman primates. *Cell* 187 (20), 5540-5553.e10. doi: 10.1016/j.cell.2024.08.043

Nayak, A. K., Chakraborty, A., Shukla, S., Kumar, N., and Samanta, S. (2024). An immunoinformatic approach for developing a multi-epitope subunit vaccine against Monkeypox virus. *Silico Pharmacol.* 12 (1), 42. doi: 10.1007/s40203-024-00220-5

Oladipo, E. K., Oyelakin, O. D., Aiyelabegan, A. O., Olajide, E. O., Olatayo, V. O., Owolabi, K. P., et al. (2024). Exploring computational approaches to design mRNA Vaccine against vaccinia and Mpox viruses. *Immunity, Inflamm. Dis.* 12 (8), e1360. doi: 10.1002/iid3.1360

Pirmoradi, S. (2022). Designing a new Vaccine Based on Multiple Epitopes Against Monkeypox Virus with the help of new Methods Based on Immunoinformatics Software. *Iran. J. Pharm. Sci.* 18 (2), 160–175. doi: 10.22034/ijps.2022.704326

Pritam, M. (2023). Exploring the whole proteome of monkeypox virus to design B cell epitope-based oral vaccines using immunoinformatics approaches. *Int. J. Biol. Macromol.* 252, 126498. doi: 10.1016/j.ijbiomac.2023.126498

Ramprasadh, S. V., Rajakumar, S., Srinivasan, S., Susha, D., Sharma, S., and Chourasiya, R. (2023). Computer-Aided Multi-Epitope Based Vaccine Design Against Monkeypox Virus Surface Protein A30L: An Immunoinformatics Approach. *Protein J.* 42 (6), 645–663. doi: 10.1007/s10930-023-10150-4

Rcheulishvili, N., Mao, J., Papukashvili, D., Feng, S., Liu, C., Wang, X., et al. (2023a). Design, evaluation, and immune simulation of potentially universal multi-epitope mpox vaccine candidate: focus on DNA vaccine. *Front. Microbiol.* 14, 1203355. doi: 10.3389/fmicb.2023.1203355

Rcheulishvili, N., Mao, J., Papukashvili, D., Feng, S., Liu, C., Yang, X., et al. (2023b). Development of a Multi-Epitope Universal mRNA Vaccine Candidate for Monkeypox, Smallpox, and Vaccinia Viruses: Design and In Silico Analyses. *Viruses* 15 (5), 1120. doi: 10.3390/v15051120

Ren, Z., Li, M., Chen, J., Gong, X., Song, S., Li, D., et al. (2024). Identification of mpox M1R and B6R monoclonal and bispecific antibodies that efficiently neutralize authentic mpox virus. *Emerg. Microbes Infect.*, 2401931. doi: 10.1080/22221751.2024.2401931

Sanami, S., Nazarian, S., Ahmad, S., Raeisi, E., ul Qamar, M. T., Tahmasebian, S., et al. (2023). *In silico* design and immunoinformatics analysis of a universal multi-epitope vaccine against monkeypox virus. *PLoS One* 18 (5), e0286224. doi: 10.1371/journal.pone.0286224

Sang, Y., Zhang, Z., Liu, F., Lu, H., Yu, C., Sun, H., et al. (2023). Monkeypox virus quadrivalent mRNA vaccine induces immune response and protects against vaccinia virus. *Signal Transduct. Target. Ther.* 8 (1), 172. doi: 10.1038/s41392-023-01432-5

Shah, M., Jaan, S., Shehroz, M., Sarfraz, A., Asad, K., Wara, T. U., et al. (2023). Deciphering the Immunogenicity of Monkeypox Proteins for Designing the Potential mRNA Vaccine. *ACS Omega* 8 (45), 43341–43355. doi: 10.1021/acsomega.3c07866

Shantier, S. W., Mustafa, M. I., Abdelmoneim, A. H., Fadl, H. A., Elbager, S. G., and Makhawi, A. M. (2022). Novel multi epitope-based vaccine against monkeypox virus: vaccinomic approach. *Sci. Rep.* 12 (1), 15983. doi: 10.1038/s41598-022-20397-z

Singh, S., Rao, A., Kumar, K., Mishra, A., and Prajapati, V. K. (2023). Translational vaccinomics and structural filtration algorithm to device multiepitope vaccine for catastrophic monkeypox virus. *Comput. Biol. Med.* 153, 106497. doi: 10.1016/j.compbiomed.2022.106497

Su, C., Li, S., Wen, Y., Geng, X., Yin, Q., Wang, Y., et al. (2024). A Quadrivalent mRNA Immunization Elicits Potent Immune Responses against Multiple Orthopoxviral Antigens and Neutralization of Monkeypox Virus in Rodent Models. *Vaccines* 12 (4), 385. doi: 10.3390/vaccines12040385

Suleman, M., Rashid, F., Ali, S., Sher, H., Luo, S., Xie, L., et al. (2022). Immunoinformatic-based design of immune-boosting multiepitope subunit vaccines against monkeypox virus and validation through molecular dynamics and immune simulation. *Front. Immunol.* 13, 1042997. doi: 10.3389/fimmu.2022.1042997

Swetha, R. G., Basu, S., Ramaiah, S., and Anbarasu, A. (2022). Multi-Epitope Vaccine for Monkeypox Using Pan-Genome and Reverse Vaccinology Approaches. *Viruses* 14 (11), 2504. doi: 10.3390/v14112504

Tan, C., Zhou, J., Wu, A., and Li, C. (2024). In silico development of a novel anti-mutation, multi-epitope mRNA vaccine against MPXV variants of emerging lineage and sub-lineages by using immunoinformatics approaches. *J. Biomol. Struct. Dyn.* doi: 10.1080/07391102.2024.2325109

Tan, C., Zhu, F., Pan, P., Wu, A., and Li, C. (2023). Development of multi-epitope vaccines against the monkeypox virus based on envelope proteins using immunoinformatics approaches. *Front. Immunol.* 14, 1112816. doi: 10.3389/fimmu.2023.1112816

Tang, D., Liu, X., Lu, J., Fan, H., Xu, X., Sun, K., et al. (2023). Recombinant proteins A29L, M1R, A35R, and B6R vaccination protects mice from mpox virus challenge. *Front. Immunol.* 14, 1203410. doi: 10.3389/fimmu.2023.1203410

Tian, Y., Li, M., Yang, Y., Li, C., Peng, Y., Yang, H., et al. (2024). An MPXV mRNA-LNP vaccine candidate elicits protective immune responses against monkeypox virus. *Chinese Chem. Lett.* 35 (8), 109270. doi: 10.1016/j.cclet.2023.109270

Ullah, A., Shahid, F. A., Haq, M. U., Tahir ul Qamar, M., Irfan, M., Shaker, B., et al. (2023). An integrative reverse vaccinology, immunoinformatic, docking and simulation approaches towards designing of multi-epitopes based vaccine against monkeypox virus. *J. Biomol. Struct. Dyn.* 41 (16), 7821–7834. doi: 10.1080/07391102.2022.2125441

Wang, H., Yin, P., Zheng, T., Qin, L., Li, S., Han, P., et al. (2024). Rational design of a ‘two-in-one’ immunogen DAM drives potent immune response against mpox virus. *Nat. Immunol.* 25 (2), 307–315. doi: 10.1038/s41590-023-01715-7

Waqas, M., Aziz, S., Liò, P., Khan, Y., Ali, A., Iqbal, A., et al. (2023). Immunoinformatics design of multivalent epitope vaccine against monkeypox virus and its variants using membrane-bound, enveloped, and extracellular proteins as targets. *Front. Immunol.* 14, 1091941. doi: 10.3389/fimmu.2023.1091941

Xia, H., He, Y. R., Zhan, X. Y., and Zha, G. F. (2023). Mpox virus mRNA-lipid nanoparticle vaccine candidates evoke antibody responses and drive protection against the Vaccinia virus challenge in mice. *Antiviral Res.* 216, 105668. doi: 10.1016/j.antiviral.2023.105668

Yang, X., Hu, C., Yang, X., Yang, X., Hu, X., Wang, X., et al. (2023a). Evaluation and comparison of immune responses induced by two Mpox mRNA vaccine candidates in mice. *J. Med. Virol.* 95 (10), e29140. doi: 10.1002/jmv.29140

Yang, X., Sun, Y., Gu, H., Li, D., Zhang, L., Li, T., et al. (2024). Multi-Component Protein Vaccine Induces a Strong and Long-Term Immune Response Against Monkeypox Virus. *Vaccines* 12 (12), 1410. doi: 10.3390/vaccines12121410

Yang, X., Yang, X., Du, S., Hu, C., Yang, X., Wang, X., et al. (2023b). A Subunit Vaccine Candidate Composed of Mpox Virus A29L, M1R, A35R, and B6R Elicits Robust Immune Response in Mice. *Vaccines* 11 (9), 1420. doi: 10.3390/vaccines11091420

Yaseen, A. R., Suleman, M., Jabeen, A., Nezami, L., Qadri, A. S., Arif, A., et al. (2024). Design and computational evaluation of a novel multi-epitope hybrid vaccine against monkeypox virus: Potential targets and immunogenicity assessment for pandemic preparedness. *Biologicals* 86, 101770. doi: 10.1016/j.biologicals.2024.101770

Ye, Q., Zhang, D., Zhang, R. R., Xu, Q., Huang, X. Y., Huang, B., et al. (2024a). A penta-component mpox mRNA vaccine induces protective immunity in nonhuman primates. *Nat. Commun.* 15 (1), 10611. doi: 10.1038/s41467-024-54909-4

Ye, T., Zhou, J., Guo, C., Zhang, K., Wang, Y., Liu, Y., et al. (2024b). Polyvalent mpox mRNA vaccines elicit robust immune responses and confer potent protection against vaccinia virus. *Cell Rep.* 43 (6), 114269. doi: 10.1016/j.celrep.2024.114269

Yousaf, M., Ismail, S., Ullah, A., and Bibi, S. (2022). Immuno-informatics profiling of monkeypox virus cell surface binding protein for designing a next generation multi-valent peptide-based vaccine. *Front. Immunol.* 13, 1035924. doi: 10.3389/fimmu.2022.1035924

Yu, C., Wu, Q., Xin, J., Yu, Q., Ma, Z., Xue, M., et al. (2024). Designing a smallpox B-cell and T-cell multi-epitope subunit vaccine using a comprehensive immunoinformatics approach. *Microbiol. Spectr.* 12 (6), e00465-24. doi: 10.1128/spectrum.00465-24

Zaib, S., Rana, N., Areeba, Hussain, N., Alrbyawi, H., Dera, A. A., et al. (2023). Designing multi-epitope monkeypox virus-specific vaccine using immunoinformatics approach. *J. Infect. Public Health* 16 (1), 107–116. doi: 10.1016/j.jiph.2022.11.033

Zeng, J., Li, Y., Jiang, L., Luo, L., Wang, Y., Wang, H., et al. (2023). Mpox multi-antigen mRNA vaccine candidates by a simplified manufacturing strategy afford efficient protection against lethal orthopoxvirus challenge. *Emerg. Microbes Infect.* 12 (1). doi: 10.1080/22221751.2023.2204151

Zhang, N., Cheng, X., Zhu, Y., Mo, O., Yu, H., Zhu, L., et al. (2023a). Multi-valent mRNA vaccines against monkeypox enveloped or mature viron surface antigens demonstrate robust immune response and neutralizing activity. *Sci. China Life Sci.* 66 (10), 2329–2341. doi: 10.1007/s11427-023-2378-x

Zhang, R. R., Wang, Z. J., Zhu, Y. L., Tang, W., Zhou, C., Zhao, S. Q., et al. (2023b). Rational development of multicomponent mRNA vaccine candidates against mpox. *Emerg. Microbes Infect.* 12 (1), 2192815. doi: 10.1080/22221751.2023.2192815

Zhao, R., Wu, L., Sun, J., Liu, D., Han, P., Gao, Y., et al. (2024). Two noncompeting human neutralizing antibodies targeting MPXV B6 show protective effects against orthopoxvirus infections. *Nat. Commun.* 15 (1), 4660. doi: 10.1038/s41467-024-48312-2

Zhou, J., Ye, T., Yang, Y., Li, E., Zhang, K., Wang, Y., et al. (2024). Circular RNA vaccines against monkeypox virus provide potent protection against vaccinia virus infection in mice. *Mol. Ther.* 32 (6), 1779–1789. doi: 10.1016/j.ymthe.2024.04.028

Zuiani, A., Dulberger, C. L., De Silva, N. S., Marquette, M., Lu, Y. J., Palowitch, G. M., et al. (2024). A multivalent mRNA monkeypox virus vaccine (BNT166) protects mice and macaques from orthopoxvirus disease. *Cell* 187 (6), 1363-1373.e12. doi: 10.1016/j.cell.2024.01.017
